# Angiomyolipoma rebound tumor growth after discontinuation of everolimus in patients with tuberous sclerosis complex or sporadic lymphangioleiomyomatosis

**DOI:** 10.1371/journal.pone.0201005

**Published:** 2018-09-07

**Authors:** John J. Bissler, Norio Nonomura, Klemens Budde, Bernard A. Zonnenberg, Michael Fischereder, Maurizio Voi, Anne-Laure Louveau, Fabian Herbst, E. Martina Bebin, Paolo Curatolo, Andrea Zonta, Elena Belousova

**Affiliations:** 1 University of Tennessee Health Science Center, St. Jude Children’s Research Hospital and Le Bonheur Children’s Hospital, Memphis, Tennessee, United States of America; 2 Osaka University Hospital, Department of Urology, Yamada-oka, Suita, Osaka, Japan; 3 Charite-Universitatsmedizin, Universitaetsmedizin, Berlin, Germany; 4 University Medical Center Utrecht, Department of Internal Medicine, Utrecht, Netherlands; 5 Division of Nephrology, Medizinische Klinik IV, Klinikum der Ludwig-Maximilian’s Universitaet, Munich, Germany; 6 Novartis Pharma, Oncology, East Hanover, New Jersey, United States of America; 7 Novartis Pharma S.A.S., Rueil-Malmaison Cedex, France; 8 Novartis Pharma AG, Basel, Switzerland; 9 University of Alabama at Birmingham, Birmingham, Alabama, United States of America; 10 Tor Vergata University Hospital, Rome, Italy; 11 Department of Medical Sciences, University of Torino, Torino, Italy; 12 Schlumberger Moscow Research Center, Moscow Research Institute of Pediatrics and Pediatric Surgery, Moscow, Russian Federation; Medizinische Universitat Graz, AUSTRIA

## Abstract

**Introduction:**

The EXIST-2 (NCT00790400) study demonstrated the superiority of everolimus over placebo for the treatment of renal angiomyolipomas associated with tuberous sclerosis complex (TSC) or sporadic lymphangioleiomyomatosis (LAM). This post hoc analysis of EXIST-2 study aimed to assess angiomyolipoma tumor behavior among patients who submitted to continued radiographic examination following discontinuation of everolimus in the noninterventional follow-up phase.

**Methods:**

For patients who discontinued everolimus at the completion of extension phase for reasons other than angiomyolipoma progression, a single CT/MRI scan of the kidney was collected after 1 year of treatment discontinuation. Changes from baseline and from the time of everolimus discontinuation in the sum of volumes of target angiomyolipoma lesions were assessed in the non-interventional follow-up phase (data cutoff date, November 6, 2015).

**Results:**

Of the 112 patients who received ≥1 dose of everolimus and discontinued treatment by the end of extension phase, 34 (30.4%) were eligible for participation in the non-interventional follow-up phase. Sixteen of 34 patients were evaluable for angiomyolipoma tumor behavior as they had at least one valid efficacy assessment (i.e. kidney CT/MRI scan) after everolimus discontinuation. During the non-interventional follow-up phase, compared with baseline, two patients (12.5%) experienced angiomyolipoma progression (angiomyolipoma-related bleeding [n = 1], increased kidney volume [n = 1]). Five patients out of 16 (31.3%) experienced angiomyolipoma progression when compared with the angiomyolipoma tumor assessment at everolimus discontinuation. The median (range) percentage change in angiomyolipoma tumor volume (cm^3^) from baseline was −70.56 (−88.30; −49.64) at time of everolimus discontinuation (n = 11), and −50.55 (−79.40; −23.16) at week 48 (n = 7) after discontinuation of everolimus. One patient death was reported due to angiomyolipoma hemorrhage.

**Conclusions:**

Angiomyolipoma lesions displayed an increase in volume following discontinuation of everolimus in patients with renal angiomyolipoma or sporadic LAM associated with TSC, but there was no evidence of rapid regrowth.

**Trial registration:**

ClinicalTrials.gov NCT00790400

## Introduction

Angiomyolipomata are the most common renal manifestations in patients with tuberous sclerosis complex (TSC) developing during later childhood and adolescence.[1, 2] Renal angiomyolipomata are benign tumors composed of blood vessels, smooth muscle, and adipose tissue.[3] They belong to a family of neoplasms called perivascular epithelioid cell tumors (PEComas) which include pulmonary lymphangioleiomyomatosis (LAM).[3] LAM is a progressive lung disease characterized by infiltration of smooth muscle cells and formation of parenchymal cysts. The cells comprising the LAM lesions and angiomyolipomata appear to arise from a common source.[3, 4] Approximately, 80% of patients with TSC develop renal angiomyolipomata and mostly because of a definitive mutation of the *TSC1* or *TSC2* gene.[4] Renal angiomyolipomata may occur unilaterally or bilaterally and historically were the most common cause of premature mortality in adults with TSC.[5] Angiomyolipomata are mostly asymptomatic and slow growing. Studies that have explored the growth rates of angiomyolipomata showed mean growth rates within a range of ≥0.25 cm/year, 0.37 cm/year, and 0.5 cm/year.[6–8] Angiomyolipomata display linear growth and tend to be significantly more symptomatic as they grow.[6, 9] Large angiomyolipomata measuring >3 to 4 cm in diameter may develop a vascular aneurysm and life-threatening hemorrhage or compress normal renal tissue, potentially leading to renal failure.[5, 10] Treatment with mammalian target of rapamycin (mTOR) inhibitors is recommended for asymptomatic, growing angiomyolipomata measuring larger than 3 cm in diameter.[11] In the phase 3 EXIST-2 trial, the mTOR inhibitor everolimus reduced renal angiomyolipoma volume by ≥50% from baseline to the end of the core phase in 42% of patients after a median treatment duration of 38 weeks. Long-term results from this study demonstrated sustained tumor regression with everolimus. The proportion of patients with a ≥50% reduction in renal angiomyolipoma increased over time to 54% at ~2.5 years, and 58% at ~4 years.[12, 13] Although angiomyolipoma lesions shrink and stabilize with mTOR inhibitor treatment, there is a tendency to increase volume after the therapy is discontinued.[4, 14] Limited evidence is currently available to understand the rebound growth following discontinuation.[14] The aim of this post hoc analysis of the EXIST-2 study was to better characterize tumor response status for up to 1-year after discontinuation of everolimus treatment in patients with renal angiomyolipoma associated with TSC or sporadic LAM who submitted to continued radiographic assessment in the non-interventional follow-up phase.

## Methods

EXIST-2 (NCT00790400) was a prospective, phase 3, multicenter, randomized, double-blind trial with a core phase followed by an extension phase and a non-interventional follow-up phase. A complete description of the study participants, design, and outcomes has been published.[13, 15] In brief, eligible patients were ≥18 years of age with at least one renal angiomyolipoma measuring ≥3 cm in its longest diameter, as measured by computed tomography (CT) or magnetic resonance imaging (MRI), and had a definitive diagnosis of TSC according to the TSC consensus criteria or sporadic LAM as confirmed by biopsy or chest CT scan. Patients were excluded if they had angiomyolipoma-related bleeding or embolization during the 6 months prior to randomization or if their angiomyolipoma required surgery at the time of randomization or had impaired lung function. The primary analysis of the data from core phase (data cutoff June 30, 2011) favored everolimus over placebo in reducing angiomyolipoma volume. The study was then unblinded on September 9, 2011 and all patients (including those who had received placebo during the core phase) received everolimus in the open-label extension phase, until 4 years after the last patient was randomized (last patient, last treatment, January 28, 2015). Everolimus was initiated at oral dose of 10 mg/day and dose adjusted based on tolerability. Patients were treated with everolimus until angiomyolipoma progression, intolerable toxicity or any other reason for discontinuation. The definition of angiomyolipoma progression is described in detail previously.[13, 15]

All patients who had either completed the extension phase or who discontinued everolimus for reasons other than angiomyolipoma progression or due to adverse events (AEs) were allowed to participate in the non-interventional follow-up phase. Patients were excluded to participate in the non-interventional follow-up phase if they started treatment with systemic mTOR inhibitors, or had embolization or angiomyolipoma requiring partial/complete nephrectomy immediately after everolimus discontinuation. A single kidney CT/MRI scan was to be performed approximately one year after the discontinuation of treatment with everolimus in the non-interventional follow-up phase. If a patient needed any intervention following treatment discontinuation, a kidney CT/MRI was to be performed prior to the start of intervention, and then the non-interventional follow-up phase ended. Interventions included commercial everolimus, off label rapamycin, embolization, or nephrectomy. Data collected during the non-interventional follow-up phase included angiomyolipoma-related bleeding events, AEs (only spontaneously reported by the patient were captured), use of concomitant medications and non-drug therapies for the treatment of angiomyolipoma or angiomyolipoma-related disease progression. End-of study assessment was planned after all patients have either completed the one-year non-interventional follow-up phase or have exited the study. In addition to the initial definition based on baseline volume, a sensitivity analysis was performed using a different definition for angiomyolipoma progression during the non-interventional follow-up phase: angiomyolipoma volume increased by ≥25% from end of treatment or ≥20% increase in the volume of either kidney from end of treatment, appearance of new angiomyolipoma ≥1 cm, an increase in the longest diameter of angiomyolipoma lesion that was <1 cm at the end of treatment to ≥1 cm during the non-interventional follow-up phase, or grade ≥2 angiomyolipoma-related bleeding according to the NCI-CTCAE, version 3.0.[16] Angiomyolipoma progression was presented as a percentage rate along with an exact 95% confidence interval (CI) obtained using the Clopper-Pearson method. Baseline data was summarized by descriptive statistics. The assessment of efficacy and safety was restricted to patients without angiomyolipoma progression or anti-TSC therapy (non-study systemic anti-angiomyolipoma therapies or angiomyolipoma-related surgeries) at the time of everolimus discontinuation. Patients with at least one valid efficacy assessment during the non-interventional follow-up period were evaluable for efficacy analysis. Statistical analysis was performed using SAS software (SAS Institute Inc., Cary, NC, USA). Here we present the angiomyolipoma tumor response status among patients who submitted to continued radiographic assessment following discontinuation of everolimus in the non-interventional follow-up phase at the data cut-off date of November 6, 2015.

All patients and or legal representatives provided written informed consent according to local guidelines before enrolment and before participating in the non-interventional follow-up phase. Independent ethics committees and/or local review boards approved the protocol (S1 and S2 Appendices), which was executed according to the International Conference on Harmonization Good Clinical Practice guidelines.

## Results

**Fig 1:**
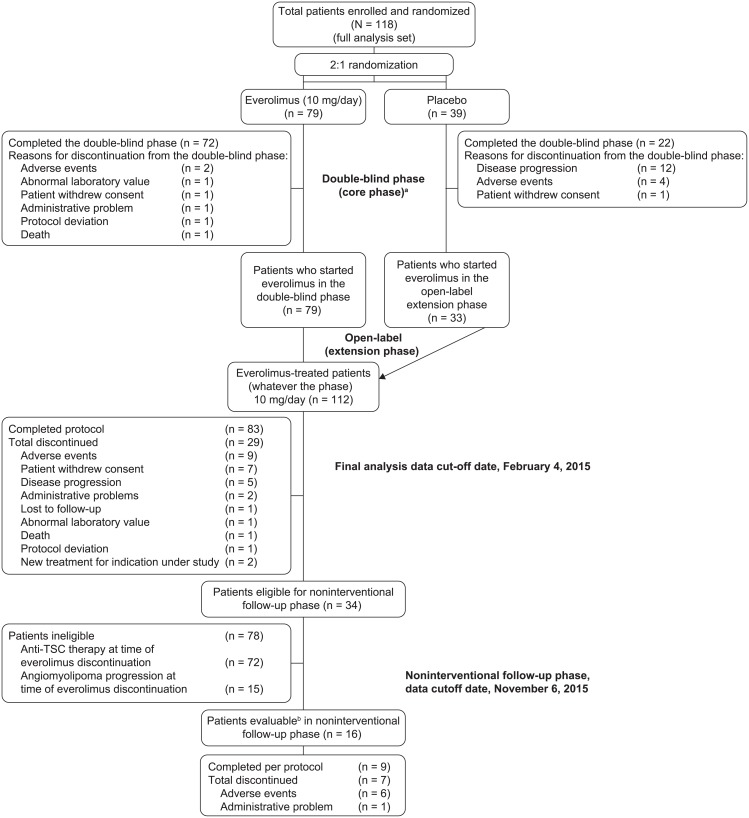
CONSORT flow diagram of patient disposition in the double-blind core phase followed by open-label extension phase and noninterventional follow-up phase. ^a^Patients with angiomyolipoma progression were unblinded at the end of the double-blind phase, and patients who were on placebo were allowed to cross over to open-label everolimus. ^b^Patients were evaluable for noninterventional follow-up phase as they had at least one adequate efficacy assessment (CT/MRI scan).

A total of 118 patients with renal angiomyolipoma associated with TSC or sporadic LAM were enrolled from 24 centers in 11 countries between April 28, 2009 and December 30, 2010. Overall, 112 patients received at least one dose of everolimus during the core phase and/or the extension phase, and had discontinued treatment with everolimus by the end of extension phase. Only 34 patients out of 112 (30.4%) were eligible for assessments in the noninterventional follow-up phase (Fig 1).

The remaining 78 patients (69.6%) were not eligible either because they received commercial everolimus (or another anti-TSC therapy) after everolimus discontinuation (64.3%) or had an angiomyolipoma progression at the time of everolimus discontinuation (13.4%). The most common reason for everolimus discontinuation among the 34 patients was AEs, reported in nine patients (26.5%). Thirteen patients completed the extension phase as per the study protocol, six withdrew consent, one withdrew due to protocol deviation, two withdrew due to administrative problems, one withdrew due to loss to follow-up, and one withdrew due to abnormal laboratory value. One patient (originally randomized to everolimus) with a history of intractable seizures died; this fatal case was reported with the initial study report.[13] Only 16 patients of the 34 were evaluable for efficacy as they had at least one valid efficacy assessment (i.e. kidney CT/MRI scan) after everolimus discontinuation. Eleven patients were randomized to everolimus starting in the core phase while the remaining five patients were randomized to placebo and switched to everolimus starting in the extension phase. The reasons for treatment discontinuation for the 16 patients were treatment completion as per protocol (56.3%), AEs (37.5%), and administrative problems (6.3%).

The median age of the 16 patients evaluable for efficacy in the non-interventional follow-up phase was 34.9 years (range, 20.5–49.4) at baseline and 38.2 years (range, 25.1–52.1) at end of treatment, and 50% were female (Table 1). The median sum of volumes of target lesions at baseline was 154.67 cm^3^ (range, 24.1–861.4). The median duration of study follow-up since discontinuation of everolimus was 11.1 months (range, 3.2–39.1). The median duration of everolimus exposure was 43.7 months (range, 0.46–60.5), and the median dose intensity was 9.89 mg/day (range, 3.9–10).

**Table 1:** Patient demographics and disease characteristics.

Characteristic	Evaluable patients^a^ (N = 16)
**Median age at baseline, years (range)**	34.9 (20.5–49.4)
**Median age at time of treatment discontinuation, years (range)**	38.2 (25.1–52.1)
**Sex, n (%)**	
Male	8 (50.0)
Female	8 (50.0)
**Race, n (%)**	
Caucasian	14 (87.5)
Asian	2 (12.5)
**WHO performance status at baseline, n (%)**	
**0**	11 (68.8)
**1**	4 (25.0)
2	1 (6.3)
**Median body mass index at baseline, kg/m**^**2**^ **(range)**	24.25 (19.8–29.0)
**Median body surface area at baseline, m**^**2**^ **(range)**	1.83 (1.62–2.14)
**Sum of volumes of target renal angiomyolipoma lesions, cm**^**3**^	
Mean (standard deviation)	248.72 (254.9)
Median (range)	154.67 (24.1–861.4)

^a^Patients with at least one valid efficacy assessment during the non-interventional follow-up phase.

### Efficacy outcomes

Angiomyolipoma tumor progression at the time of primary analysis was observed in 3 patients treated with everolimus (3/79, 3.8%) and 8 patients treated with placebo (8/39, 20.5%).[13] At the end of the extension phase, 16 patients reported angiomyolipoma progression (16/112, 14.3%).[17] At the end of the non-interventional follow-up phase, angiomyolipoma progression was observed in 2/16 patients (12.5%) when compared to the baseline. Among the 2 patients, one patient was already considered to have angiomyolipoma progression at the end of the extension phase. Primary reasons for progression in 2 patients were angiomyolipoma-related bleeding of grade ≥2 in one patient (6.3%) and increase in kidney volume only in another patient (6.3%). Further detailed evaluation of the CT scans by the investigator for the patient with increased kidney volume revealed an increase in cyst volume which may have contributed to the overall kidney volume increase as the kidney volume measurements also included cyst volume. However, cyst volume was not centrally collected and assessed separately. Five patients (31.3%) reported angiomyolipoma progression at the end of the non-interventional follow-up phase when compared with the end of treatment, as per the sensitivity analysis. The most common reasons for progression in these 5 patients included increase in both tumor and kidney volume in three patients (18.8%), angiomyolipoma-related bleeding of grade ≥2 in one patient (6.3%), and increase in tumor volume only in one patient (6.3%).

The median percentage change in the sum of volumes of target angiomyolipoma lesions (cm^3^) from baseline (119.31; range, 24.06–861.36) to end of treatment (27.49; range, 4.03–253.61) was −70.56% (range, −88.30 to −49.64; n = 11 evaluable patients), and from baseline (146.79; range, 24.06–861.36) to week 48 of non-interventional follow-up phase (87.32; range, 4.96–312.68) was −50.55% (range, −79.40 to −23.16; n = 7 evaluable patients; Table 2). The median percentage change in the sum of volumes of target angiomyolipoma lesions (cm^3^) from end of treatment (46.63; range, 4.03–253.61) to week 48 of the non-interventional follow-up phase (87.32; range, 4.96–312.68) was 52.53% (range, −23.16 to 87.25; n = 7 evaluable patients; Table 2).

**Table 2:** Median sum of volumes of target angiomyolipoma lesions at baseline, EOT, and week 48 of non-interventional follow-up phase, and percentage change from baseline to EOT/week 48 of non-interventional follow-up phase and from EOT to week 48 of non-interventional follow-up phase.

Median (range)	Evaluable patients at EOT (N = 11)	Evaluable patients at week 48 of non-interventional follow-up phase (N = 7)
**Sum of volumes at baseline, cm**^**3**^	119.31 (24.06–861.36)	146.79 (24.06–861.36)
**Sum of volumes at EOT, cm**^**3**^	27.49 (4.03–253.61)	46.63 (4.03–253.61)
**Sum of volumes at week 48 of non-interventional follow-up phase, cm**^**3**^	NA	87.32 (4.96–312.68)
**Percentage change from baseline, %**	−70.56 (−88.30 to −49.64)	−50.55 (−79.40–23.16)
**Percentage change from EOT, %**	NA	52.53 (−23.16–87.25)

Abbreviations: EOT = end of treatment; NA = not applicable.

### Safety outcomes

Safety results during treatment with everolimus were reported in detail in prior publications.[12, 13, 15] No renal hemorrhages were observed during the cumulative 390.8 patient-years of everolimus treatment.[17] During the non-interventional follow-up phase, one patient died after 5.8 months of treatment discontinuation due to angiomyolipoma hemorrhage. The patient was exposed to everolimus treatment for approximately 4.1 years. Five patients reported AEs after 28-days of treatment discontinuation which included post-procedural complications, procedural pain and arthralgia for the same patient, hypersensitivity, decrease in weight, anemia and hemorrhage reported in one patient each.

## Discussion

This post hoc analysis of EXIST-2 demonstrated tumor growth after discontinuation of everolimus in patients with renal angiomyolipoma associated with TSC or sporadic LAM. However, there was no evidence of rapid regrowth as the increase in angiomyolipoma lesion volume following treatment discontinuation did not exceed the lesion volume at baseline, even among patients who displayed progression. The angiomyolipoma progression rates were comparable between patients of the non-interventional follow-up phase (31.3%) and the patients treated with placebo during the double-blind core phase (20.5%).[13] To the best of our knowledge, this is the first ever study of everolimus reporting the tumor behavior after treatment discontinuation. Previously, an increase in angiomyolipoma volume was reported with discontinuation from sirolimus therapy, but did not return completely to the baseline values. The reason for angiomyolipoma progression could be due to impaired apoptosis or increase in angiomyolipoma size or volume.[4] In EXIST-2, 12 of the 16 evaluable patients experienced an increase in angiomyolipoma volume compared to their most recent tumor volume assessed before treatment discontinuation (EOT). A median angiomyolipoma growth of 52.53% was observed at 48 weeks after everolimus discontinuation.

Long-term safety analysis of EXIST-2 had shown that everolimus was well-tolerated and the AEs remained consistent with the previous reports.[13, 15] Angiomyolipoma-related bleeding event was reported only in one patient during the non-interventional follow-up phase, and during an overall 390.8 patient-years of treatment with everolimus, no bleeding events were reported.[17] Six patients reported angiomyolipoma-related bleeding events in the year before randomization. These observations suggest a considerably lower annual rate of angiomyolipoma-related bleeding for patients receiving everolimus when compared with patients not receiving everolimus either before or after the treatment portion of the study. Previous studies suggested a correlation between angiomyolipoma size and risk of hemorrhage.[18] The aneurysm and tumor sizes are considered as the main predictors of renal hemorrhage.[18] Angiomyolipomata tend to become symptomatic as they grow. Previous studies showed tumor growth in 27% to 53% of lesions measuring <4 cm and in 46% to 75% of lesions measuring >4 cm over a follow-up period of up to 4 years.[7, 8] This suggests that treatment that maintains or reduces angiomyolipoma lesions may reduce the risk of bleeding.

Fewer evaluable patients to assess tumor response after treatment discontinuation is a limitation of this analysis as more robust predictions of tumor behavior after cessation of everolimus treatment are difficult. In summary, angiomyolipoma lesion volume increased for up to one year after discontinuation of everolimus in patients with renal angiomyolipoma associated with TSC or sporadic LAM, compared to their most recent tumor volume assessment performed prior to treatment discontinuation; though the angiomyolipoma volume did not exceed that measured at baseline. The results of our study show that persistence of clinically significant angiomyolipoma volume reduction may require ongoing treatment with everolimus in patients with TSC.

## Supporting information

S1 Appendix List of ethics committees and/or intuitional review boards that approved the EXIST-2 study. (DOCX)

S2 Appendix EXIST-2 study protocol. (PDF)

S3 Appendix CONSORT 2010 checklist of information to include when reporting a randomized trial. (DOC)
