# Supplementary material for: Angiomyolipoma rebound tumor growth after discontinuation of everolimus in patients with tuberous sclerosis complex or sporadic lymphangioleiomyomatosis
Source: PLoS One. 2018 Sep 7;13(9):e0201005. doi: 10.1371/journal.pone.0201005 (PMC6128468; doi:10.1371/journal.pone.0201005)
Supplement: S1 Appendix — (DOCX) [file pone.0201005.s001.docx]

**S1 Table**. List of Independent Ethics Committees (IEC) or Institutional Review Boards (IRB) by study center

| **Center No.** | **Ethics Committee or**  **Institutional Review Board** | **City, State/Province,**  **Postal Code**  **Country** |
| --- | --- | --- |
| 0100 | University Health Network  Research Ethics Board  8th Floor South, Room 8-23,  700 University Ave | Ontario M5G 1Z5  Canada |
| 0154 | CPP "Sud-Est IV" LYON Centre Régional de Lutte  Contre le cancer Léon Bérard  28 rue Laennec  69373 LYON Cedex 08 | Lyon 69373  France |
| 0201 | Landesamt für Gesundheit  und Soziales  Ethik-Kommission des  Landes Berlin  Fehrbelliner Platz 1  10707 Berlin | Berlin 10707  Germany |
| 0202 | Ludwig-Maximilians-  Universität München  Klinikum der Universität  Ethik-Kommission  Pettenkoferstr. 8a  80336 München | Munchen 80336  Germany |
| 0251 | Comitato Indipendente  presso la Fondazione PTV  Policlinico Tor Vergata Di  Roma Viale Oxford, 81  00133 Roma (RM) | Roma 00133  Italy |
| 0253 | Comitato Etico  Interaziendale A.O. Citta'  Della Salute E Della Scienza  Di Torino Corso Bramante,  88/90 10126 Torino (TO) | Torino 10126  Italy |
| 0254 | Comitato Etico Locale Per  La Sperimentazione Clinica  Dei Medicinali Dell’Azienda  Ospedaliera Universitaria  Senese Di Siena c/o UOC  Farmacia AOUS - Viale  Bracci  53100 SIENA (SI) | SIENA 53100  Italy |
| 0300 | METC UMCU  Huispost D 01.343  Heidelberglaan 100  3584 CX Utrecht | 3584  Netherlands |
| 0400 | Moscow Federal State  Budget Scientific Institution  of Pediatrics and Pediatrics  Surgery  2, Taldomskaya  str.,Moscow, 127412 | Moscow 127412  Russia |
| 0476 | The Bioethics Committee at  Instytut, Pomnik- Centrum  Zdrowia Dziecka”  Al. Dzieci Polskich 20; 04-  730 Warszawa-Miedzylesie | Warszawa 04-730  Poland |
| 0477 | The Bioethics Committee at  Instytut, Pomnik- Centrum  Zdrowia Dziecka”  Al. Dzieci Polskich 20; 04-  730 Warszawa-Miedzylesie | Warszawa 04-730  Poland |
| 0550 | Cincinnati Children’s  Hospital Medical Center  Institutional Review Board  3333 Burnet Avenue, MLC  5020  Cincinnati OH 45229 | Cincinnati OH 45229  USA |
| 0551 | Western Institution Review  Board (WIRB)  3535 Seventh Avenue, SW  Olympia, Washington 98502 | Washington 98502  USA |
| 0552 | Shulman Associates IRB | Lake Forest Dr Blue Ash 45242  (513) 761-4100  USA |
| 0553 | Partners Human Research  Office  116 Huntington Avenue,  Suite 1002  Boston, MA 02116 | Boston 02116 |
| 0555 | St. Joseph's Hospital and  Medical Center  Internal Review Board  350 W. Thomas Rd.  Phoenix, AZ 85013 | Phoenix 85013 |
| 0556 | University of Tennessee, Institutional Review Board;  910 Madison Avenue, Suite  600 | Memphis  United States |
| 0601 | Hokkaido University Hospital  Institutional Review Board,  Kita-14，Nishi-5，Kita-ku，  Sapporo, Hokkaido, Japan | Hokkaido  Japan |
| 0602 | Ethics Committee or  Institutional Review Board, Yamagata university hospital | Yamagata  Japan |
| 0603 | Institutional Review Board of  Osaka University, Osaka University Hospital | Osaka 565-0871  Japan |
| 0650 | Comité Etico De  Investigacion Clinica  Fundacio Puigvert  Coneixement  08025 – Barcelona Spain | Barcelona 08025 |
| 0770 | South Central –  Southampton A  South West REC Centre  Maxine Knight  Level 3, Block B  Whitefriars  Lewins Mead  Bristol | Bristol BS1 2NT  UK |
| 0772 | South Central –  Southampton A  South West REC Centre  Maxine Knight  Level 3, Block B  Whitefriars  Lewins Mead  Bristol | Bristol BS1 2NT  UK |
| 0774 | South Central –  Southampton A  South West REC Centre  Maxine Knight  Level 3, Block B  Whitefriars  Lewins Mead  Bristol | Bristol BS1 2NT  UK |
| 0775 | South Central –  Southampton A  South West REC Centre  Maxine Knight  Level 3, Block B  Whitefriars  Lewins Mead  Bristol | Bristol BS1 2NT  UK |
