# Supplementary material for: Angiomyolipoma rebound tumor growth after discontinuation of everolimus in patients with tuberous sclerosis complex or sporadic lymphangioleiomyomatosis
Source: PLoS One. 2018 Sep 7;13(9):e0201005. doi: 10.1371/journal.pone.0201005 (PMC6128468; doi:10.1371/journal.pone.0201005)
Supplement: S2 Appendix — (PDF) [file pone.0201005.s002.pdf]

NOVARTIS

Clinical Development

RAD001 (everolimus)

CRAD001M2302

A randomized, double-blind, placebo-controlled study of RAD001 in the treatment of Angiomyolipoma in patients with either Tuberous Sclerosis Complex (TSC) or Sporadic Lymphangioleiomyomatosis (LAM)

Authors:	
Document type:	Amended Protocol Version
EUDRACT number:	2008-002113-48
Version number:	05 Clean
Development phase:	III
Document status:	Final
Release date:	24-Oct-2013

[redacted]

Table of contents

Table of contents 2
List of figures 5
List of tables 6
List of abbreviations 7
Amendment 5 10
Amendment 4 ..... **Error! Bookmark not defined.**
Oncology clinical study protocol synopsis 11
1 Background 23
1.1 Overview of Tuberous Sclerosis Complex (TSC) and Lymphangioleiomyomatosis (LAM) 23
1.2 Overview of Angiomyolipomata 24
1.2.1 Choice of a minimum angiomyolipoma size 26
1.3 Volumetric assessment of tumor response 26
1.4 Overview of RAD001 27
1.4.1 RAD001 27
1.4.2 mTOR pathway and tumorigenesis 28
1.4.3 Preclinical studies 29
1.4.4 RAD001 pharmacokinetics 29
1.4.5 Safety in oncology studies 32
1.4.6 RAD001 Phase II study in patients with angiomyolipoma associated with TSC and/or LAM 34
1.4.7 Sirolimus Phase II study in patients with angiomyolipoma associated with TSC and/or LAM 35
1.5 History of Amendments 36
1.5.1 Amendment 1 ..... **Error! Bookmark not defined.**
1.5.2 Amendment 2 ..... **Error! Bookmark not defined.**
2 Study rationale/purpose 36
3 Objectives 36
3.1 Primary objective 36
3.2 Secondary objectives 36
3.3 Exploratory objectives 38
4 Study design 38
4.1 Pre-treatment phase (screening/baseline) 38
4.2 Blinded treatment phase 41
4.3 Open label treatment phase 43
4.4 Follow-up phase 44

4.5 Extension phase 44
4.6 Non-interventional follow-up phase 47
4.7 End of Study 48
4.7.1 Patient participation 48
5 Population 48
5.1 Inclusion criteria 49
5.1.1 Non-interventional follow-up phase/Inclusion criteria 50
5.2 Exclusion criteria 50
5.2.1 Non interventional follow-up phase/Exclusion criteria 52
6 Treatment 52
6.1 Investigational and control drugs 52
6.1.1 Known undesirable effects of study drug/treatment 52
6.1.2 Dosing modifications in case of treatment related toxicities 54
6.1.3 Study drug 56
6.2 Treatment arms 57
6.3 Patient numbering 57
6.4 IWRS procedure 58
6.5 Treatment assignment 58
6.6 Treatment blinding 59
6.7 Treating the patient 59
6.7.1 Study drug administration 59
6.7.2 Permitted study drug adjustments 60
6.7.3 Other concomitant medications 61
6.7.4 Study drug interruption or discontinuation 63
6.7.5 Withdrawal from the study and study evaluation completion 65
6.7.6 Emergency unblinding of treatment assignment 65
6.7.7 Treatment compliance 66
6.7.8 Non interventional follow-up phase after the end of the extension phase 66
7 Visit schedule and assessments 66
7.1 Information to be collected on screening failures 78
7.2 Inclusion/exclusion criteria 78
7.3 Patient demographics/other baseline assessments 78
7.3.1 Special laboratory tests 78
7.4 Treatments 80
7.5 Efficacy 80
7.5.1 Radiological evaluation 80

7.5.2 Angiomyolipoma response evaluation 81
7.5.3 SEGA response evaluation.... **Error! Bookmark not defined.**
7.5.4 Skin lesions 83
7.6 Safety 85
7.6.1 Adverse events 85
7.6.2 Physical examination 87
7.6.3 Vital signs....87
7.6.4 WHO performance status and scale 87
7.6.5 Laboratory evaluations....88
7.6.6 Pulmonary function tests.... **Error! Bookmark not defined.**
7.6.7 Electrocardiogram (ECG) 90
7.7 Pharmacokinetics....91
7.7.1 Pharmacokinetics blood sample collection and handling 91
7.7.2 Pharmacokinetic sample shipment....92
7.7.3 Analytical method .... **Error! Bookmark not defined.**
7.8 Biomarkers....93
7.9 Exploratory assessments....93
7.9.1 Neuropsychological assessments .... **Error! Bookmark not defined.**
7.9.2 Seizure severity questionnaire.... **Error! Bookmark not defined.**
7.9.3 Pharmacogenetics.... **Error! Bookmark not defined.**
7.9.4 Optional biomarker studies on additional or remaining samples**Error! Bookmark not d**
8 Safety monitoring 93
8.1 Serious adverse event reporting....93
8.2 Pregnancies....94
8.3 Data Monitoring Committee....95
8.4 Steering Committee 95
9 Data review and data management....96
9.1 Site monitoring 96
9.2 Data collection....96
9.3 Database management and quality control 97
10 Statistical methods and data analysis 98
10.1 Populations for analysis....98
10.2 Patient demographics/other baseline characteristics 98
10.3 Treatments (study drug, concomitant therapies, compliance)....98
10.3.1 Study medication....98
10.3.2 Concomitant therapies....98
10.4 Primary objective....99

10.4.1 Variable 99
10.4.2 Statistical hypothesis, model, and method of analysis 99
10.4.3 Handling of missing values/censoring/discontinuations 99
10.4.4 Supportive analyses 100
10.5 Secondary objectives 100
10.5.1 Time to angiomyolipoma progression 100
10.5.2 Skin lesion response rate 101
10.5.3 Multiplicity adjustment for analysis of main secondary endpoints 101
10.5.4 Additional secondary efficacy analyses 102
10.5.5 Safety 103
10.5.6 Tolerability 104
10.5.7 Resource utilization 104
10.5.8 Patient-reported outcomes 104
10.5.9 Pharmacokinetics 104
10.5.10 Biomarkers 104
10.5.11 Evaluation after everolimus discontinuation 105
10.6 Interim analysis 106
10.7 Sample size calculation 106
11 Administrative procedures 108
12 Protocol adherence 110
13 References (available upon request) 111

List of figures

Figure 4-1 Study flowchart 46
Figure 4-2 Non-intervention follow-up phase schema 47

List of tables

Table 1-1 Drug substance 29
Table 1-2 Steady-state RAD001 pharmacokinetics (daily dosing) 31
Table 1-3 Adverse events suspected to be drug related in greater or equal to 10% of patients with adverse cancer reported in Phase I RAD001 monotherapy [Studies C2101, C2102, C2107] 33
Table 1-4 Key laboratory abnormalities reported at a higher rate in the Afinitor arm than in the placebo arm 34
Table 4-1 Action to be taken for positive baseline hepatitis B results for patients that are active prior to Amendment 1 approval 39
Table 4-2 Guidelines for management of hepatitis B for patients that are active prior to Amendment 1 approval 40
Table 4-3 Guidelines for management of hepatitis C for patients that are active prior to Amendment 1 approval 40
Table 5-1 Diagnostic criteria for Tuberous Sclerosis Complex 50
Table 6-1 Everolimus dose modification guidelines for non-hematologic toxicities 54
Table 6-2 Dose modification guidelines for hematologic toxicities 55
Table 6-3 Management of non-infectious pneumonitis 56
Table 6-4 RAD001 dose levels for dose adjustment 61
Table 6-5 Clinically relevant drug interaction: inducers and inhibitors of isoenzyme CYP3A 63
Table 6-6 Clinically relevant drug interactions mediated by PgP 63
Table 7-1 Blinded phase visit evaluation schedule 67
Table 7-2 Open label and extension phase visit evaluation schedule 72
Table 7-3 Non-interventional follow-up phase visit evaluation schedule 77
Table 7-4 Physician’s Global Assessment of Clinical Condition (PGA) 85
Table 7-5 PK sample log table 91
Table 10-1 Definition of potential prognostic factors 100
Table 10-2 Sensitivity of study power to treatment by stratum interaction assuming 3:3:2 ratio of patients across strata 1-3 107
Table 10-3 Sensitivity of study power to size of treatment effect 108

List of abbreviations

AACR	American Association for Cancer Research
Ab	Antibodies
ADR	Adverse Drug Reaction
AE	Adverse Event
ALT	Alanine Aminotransferase/Glutamic Pyruvic Transaminase/GPT
ANC	Absolute Neutrophil Count
ASCO	American Society of Clinical Oncology
AST	Aspartate Aminotransferase/Glutamic Oxaloacetic Transaminase/GOT
ATC	Anatomical Therapeutic Chemical
ATS	American Thoracic Society
AUC	Area Under the Curve
BAL	Bronchoalveolar Lavage
BUN	Blood Urea Nitrogen
C_{2h}	Concentration 2 hours Post-dose
CCR	Complete Clinical Response
C_{max}	Maximum Concentration
CMH	Cochran-Mantel-Haenszel
C_{min}	Minimum (trough) Concentration
CoA	Coenzyme A
CrCl	Creatinine Clearance
CRF	Case Report/Record Form
CRO	Contract Research Organization
CSF	Cerebrospinal Fluid
CT	Computed Tomography
CTCAE	Common Toxicity Criteria for Adverse Events
CTH	Clinical Trial Head
CV	Coefficient of Variation
CYP3A	Cytochrome P450 3A4
DL_{CO}	Carbon Monoxide Diffusion Capacity
DLT	Dose-limiting Toxicity
DMC	Data Monitoring Committee
DNA	Deoxyribonucleic Acid
DS&E	Drug Safety & Epidemiology
ECG	Electrocardiogram
EDTA	Ethylenediaminetetraacetic acid
EIAED	Enzyme-inducing Antiepileptic Drug
ERS	European Respiratory Society
ESRD	End-stage Renal Disease
FAS	Full Analysis Set
FDA	US Food and Drug Administration
FEV_1	Forced Expiratory Volume in One Second
FGF	Fibroblast Growth Factor
FPFV	First Patient First Visit
FSH	Follicle-stimulating Hormone
FVC	Forced Vital Capacity

GCP	Good Clinical Practice
GIST	Gastrointestinal Stromal Tumor
HA	Health Authority
HBcAb	Hepatitis B Core Antibodies
HBs Ab	Hepatitis B Surface Antibodies
HBsAg	Hepatitis B Surface Antigen
HBV	Hepatitis B virus
HCV	Hepatitis C Virus
HDL	High-density Lipoprotein
HIV	Human Immunodeficiency Virus
HMG	3 hydroxy-3 methyl -glutaryl
HUVEC	Human Umbilical Vein Endothelial Cells
IB	Investigator's Brochure
ICH	International Conference on Harmonization
IEC	Independent Ethics Committee
IN	Investigator Notification
IND	Investigational New Drug Application
INR	International Normalized Ratio
IRB	Institutional Review Board
IWRS	Interactive Web Response System
LAM	Lymphangioleiomyomatosis
LDH	Lactate Dehydrogenase
LDL	Low-density Lipoprotein
LFTs	Liver Function Tests
LLOQ	Lower Limit of Quantification
LPLV	Last Patient Last Visit
MedDRA	Medical Dictionary for Regulatory Activities
Mg/d	Milligrams per day
MRI	Magnetic Resonance Imaging
MSD	Meso Scale Discovery
mTOR	Mammalian Target of Rapamycin
NCI	National Cancer Institute
O_2	Oxygen
p.o.	per os/by mouth/orally
PD	Progressive Disease
PFT	Pulmonary Function Test
PGA	Physician's Global Assessment of Clinical Condition
PgP	P-glycoprotein
PI3K	Phosphatidylinositol 3-kinase
PK	Pharmacokinetic
PLGF	Placental Growth Factor
PPS	Per Protocol Set
PR	Partial Response
PT	Prothrombin Time
PTEN	Phosphatase and Tensin Homolog
PTT	Partial Thromboplastin Time
RBC	Red Blood Cells
RCC	Renal Cell Carcinoma

REB	Research Ethics Board
RECIST	Response Evaluation Criteria in Solid Tumors
RFS	Recurrence-free Survival
RNA	Ribonucleic acid
s.c.	Subcutaneous
SAE	Serious Adverse Event
SD	Stable Disease
SEGA	Subependymal Giant Cell Astrocytoma
SEN	Subependymal Nodule
SGOT	Serum Glutamic Oxaloacetic Transaminase
SGPT	Serum Glutamic Pyruvic Transaminase
SI	Système International
SSC	Study Steering Committee
t_{max}	Time to Maximum Drug Concentration
TSC	Tuberous Sclerosis Complex
TTAP	Time to Angiomyolipoma Progression
ULN	Upper Limit of Normal
URTI	Upper Respiratory Tract Infection
VABS	Vineland Adaptive Behavioral Scale
VEGF	Vascular Endothelial Growth Factor
WASI	Wechsler Abbreviated Scale of Intelligence
WBC	White Blood Cells
WHO	World Health Organization

Amendment 5

IRB/IEC

A copy of this amended protocol will be sent to the Institutional Review Board (IRBs)/Independent Ethics Committee (IECs) and Health Authorities.

The changes described in this amended protocol require IRB/EC approval prior to implementation. In addition, if the changes herein affect the Informed Consent, sites are required to update and submit for approval a revised Informed Consent that takes into account the changes described in this amended protocol.

Refer to Section 1.5 in protocol for summary of previous amendments.

Oncology clinical study protocol synopsis

Investigational drug	
Protocol no.	CRAD001M2302
Study phase	III
Study title	A randomized, double-blind, placebo-controlled study of RAD001 in the treatment of angiomyolipoma in patients with either tuberous sclerosis complex (TSC) or sporadic lymphangioleiomyomatosis (LAM)
Background	Tuberous sclerosis complex (TSC) is a genetic disorder with a childhood prevalence range from 1 in 6,800 to 1 in 17,300 but an accurate assessment is difficult to achieve (Yates 2006). There are two genetic loci linked to disease: the TSC1 gene on chromosome 9, and the TSC2 gene on chromosome 16. The TSC1 gene produces the protein hamartin and the TSC2 gene produces tuberin. Hamartin and tuberin form a complex that integrates signals and functions to regulate cell growth. Patients with TSC develop a variety of abnormal growths, called hamartomas, throughout their body, including the brain, kidneys, lungs, heart, and skin. Brain lesions are the primary cause of morbidity and mortality in this disorder in childhood. The next most common cause of morbidity is from renal angiomyolipomas.<br>Angiomyolipomata are composed of abnormal blood vessels, smooth muscle, and fat cells and these lesions may be solitary or multiple, or may be so numerous as to fuse and form diffuse lesions throughout the kidney. Angiomyolipomata contain tortuous, dysmorphic blood vessels, and thus have a tendency to develop aneurysms that spontaneously rupture resulting in hemorrhage, sometimes with life threatening consequences. This risk of bleeding is roughly correlated with the size of the lesions, though there is substantial variation. In order to prevent hemorrhage, large lesions are either surgically reduced or resected, or semi-invasively managed by a procedure called embolization that deprives the angiomyolipoma of its blood supply. Vascular issues often complicate surgical interventions, and can necessitate the removal of the entire kidney to control intraoperative bleeding.<br>In women with tuberous sclerosis, angiomyolipomata are frequently associated with lymphangioleiomyomatosis (LAM). LAM can also exist in individuals without the TSC diagnosis, termed sporadic LAM, and arises due to mutations in tuberous sclerosis genes. LAM is a progressive pulmonary disorder characterized by smooth muscle infiltration and cystic destruction of the lung. Affected individuals develop progressive dyspnea on exertion, often punctuated by pneumothorax and chylothorax. One theory of LAM pathogenesis is that angiomyolipoma smooth muscle cells metastasize to the lung, where they proliferate and progressively replace the normal lung tissue. Through elaboration of matrix degrading enzymes or other mechanisms, progressive cystic degeneration and disruption of pulmonary lymphatics occurs. Consistent with the metastatic theory, LAM has been reported to recur following lung transplantation. Recent studies from the University of Cincinnati and elsewhere have revealed that pulmonary cystic changes consistent with LAM are present in up to 40% of women with TSC. LAM cells isolated from the lung share many phenotypic characteristics with angiomyolipoma cells, including staining with the melanocyte-derived monoclonal antibody HMB-45 and with antibodies to smooth muscle actin **and** genetic mutations in tuberous sclerosis genes. Owing to their biological similarities and growing evidence that the lung lesion in TSC may arise from angiomyolipomata, we postulate that strategies that effectively control the growth of angiomyolipoma cells may also control the growth of LAM cells.<br><br>The TSC1/TSC2 protein complex is a negative regulator of the mammalian target of rapamycin (mTOR) pathway. Hence, mutation or loss of function of either of these gene products in preclinical models is associated with increased mTOR pathway activation and heightened sensitivity to mTOR inhibitors (Astrinidis 2005, Inoki 2005, Kwiatkowski 2005). mTOR inhibition is associated with a dramatic inhibition of the phosphorylation of the ribosomal S6 protein in treated kidney lesions (S6 phosphorylation is an established pharmacodynamic marker of mTOR pathway activation status). Immunoblotting and immunohistochemical analysis revealed phosphorylation of p70S6 kinase, and the ribosomal S6 protein in angiomyolipomas

	occurring in tuberous sclerosis, indicating activation of the mTOR metabolic pathway (El-Hashemite et al 2003). Furthermore, a dramatic improvement in survival has been observed in a mouse brain model of TSC (genotype: *Tsc1^{cc} syn-cre^{+}*), with a highly statistically significant improvement in survival (p < 0.0001) associated with RAD001 treatment. The investigator also noted an improvement in behavior, weight gain and neurological phenotype. Assessment of brain pathology is currently ongoing. Finally, both estrogen and vascular endothelial growth factor (VEGF) signaling have been implicated in the pathogenesis and vascularization of TSC lesions (Astrinidis 2005, Kwiatkowski 2005). In this respect, RAD001 has been shown to inhibit both estrogen and VEGF-dependent signaling events (Boulay 2005, O'Reilly 2005). Taking all these data into account there is a strong rationale for using RAD001 for the treatment of patients with tuberous sclerosis.
Purpose/rationale	This study will evaluate the antitumor activity of RAD001 versus placebo in patients with angiomyolipoma associated with either TSC or sporadic LAM.
Objectives	**Primary Objective**: To compare the angiomyolipoma response rate on RAD001 versus placebo in patients with angiomyolipoma, associated with either TSC or sporadic LAM.<br>**Secondary objectives**:<br>To compare RAD001 versus placebo with respect to:<br>1. Time to angiomyolipoma progression.<br>2. Skin lesion response rate.<br>3. Change from baseline in plasma angiogenic molecules, e.g. VEGF, basic FGF, PLGF, soluble VEGF receptor1, and soluble VEGF receptor2.<br>4. Renal function assessed using calculated creatinine clearance.<br>5. Safety as assessed by the National Cancer Institute's (NCI) Common Toxicity Criteria for Adverse Events (CTCAE), version 3.0<br>In RAD001 treatment arm to:<br>1. Characterize the pharmacokinetics of RAD001 in this patient population, specifically in terms of exposure.<br>2. Describe the time to angiomyolipoma response, duration of angiomyolipoma response, and the duration of skin lesion response.<br>**Exploratory objectives**:

Endpoints (efficacy, safety)	**Efficacy endpoints**<br>**Primary endpoint**:<br>Angiomyolipoma response rate is defined as the proportion of patients with a reduction in angiomyolipoma volume of at least 50% relative to baseline, where angiomyolipoma volume is the sum of the volumes of all target angiomyolipomata identified at baseline, and confirmed with a second scan approximately 12 weeks later (and no sooner than 8 weeks later).<br>In addition, response requires that:<br>• no new angiomyolipomata ≥ 1.0 cm in longest diameter are identified<br>• neither kidney increases in volume by more than 20% from nadir (where nadir is the lowest kidney volume obtained for the patient, separately for each kidney, previously during the trial [including baseline])<br>• the patient does not have any angiomyolipoma-related bleeding of grade ≥ 2 (as defined by the NCICTCAE, version 3.0 (http://ctep.cancer.gov/forms/CTCAEv3.pdf).<br>Angiomyolipoma response rate will be determined in the Full Analysis Set (FAS), defined as all randomized patients analyzed according to the treatment they were assigned to at randomization.<br>**Secondary endpoints**:<br>1. Time to angiomyolipoma progression, i.e., time from randomization until the first angiomyolipoma progression.<br>Angiomyolipoma progression is defined as one or more of the following:<br>• an increase from nadir of 25% or more in angiomyolipoma volume to a value greater than baseline (where angiomyolipoma volume is the sum of the volumes of all target angiomyolipomata identified at baseline andwhere nadir is the lowest angiomyolipoma volume achieved by the patient previously in the trial (including baseline))<br>• the appearance of a new angiomyolipoma ≥ 1.0 cm in longest diameter<br>• an increase from nadir of 20% or more in the volume of either kidney to a value greater than baseline, where nadir is the lowest kidney volume obtained for the patient, separately for each kidney, previously in the trial (including baseline) - angiomyolipoma-related bleeding of grade ≥ 2 as defined by NCI CTCAE, version 3.0.<br>2. Skin lesion response rate using the Physician's Global Assessment of Clinical Condition (PGA), where response is defined as complete clinical response or partial response, and where the denominator only includes patients with at least one skin lesion at baseline.<br>3. Change from baseline in angiogenesis markers.<br>**In RAD001 treatment arm**:<br>1. Exposure of RAD001 in treated patients.<br>2. Duration of angiomyolipoma response, defined as the time from the first angiomyolipoma response until angiomyolipoma progression; time to angiomyolipoma response is the time from randomization until the first angiomyolipoma response. Duration of response of skin lesions, defined as the time from the first skin lesion response until skin lesion progression, where response and progression are based on the Physician's Global Assessment of Clinical Condition (PGA).<br>**Safety endpoints**<br>Safety will be assessed by the National Cancer Institute's (NCI) Common Terminology Criteria for Adverse Events (CTCAE), version 3.0. (http://ctep.cancer.gov/protocolDevelopment/electronic_applications/docs/ctcaev3.pdf).<br>• Safety assessments will consist of monitoring and recording all adverse events, including serious adverse events and the regular monitoring of vital signs, physical condition, hematology and blood chemistry, and body weight.<br>• Renal function will be assessed using creatinine clearance calculated from the formula of Cockcroft and Gault (Cockcroft et al 1976).

	• Pneumonitis is a known side-effect of mTOR inhibitors. To document lung function at baseline, pulmonary function tests (PFTs) will be performed at screening in all patients with LAM. For these patients, PFTs will be repeated at 6, 12, 18 and 24 weeks and, then every 12 weeks throughout the study. For patients without LAM, PFTs will not be performed at baseline or routinely repeated, but if there is clinical suspicion of pneumonitis, PFTs should be performed and a consultation with a pulmonologist arranged.
Study design	This is a prospective, double-blind, randomized, parallel group, placebo-controlled, multi-center, phase III study evaluating treatment with RAD001 versus placebo in 99 patients with angiomyolipoma associated with either TSC or sporadic LAM. This study will use an Interactive Web Response System (IWRS) for patient randomization and medication management. The randomization ratio is 2:1 in favor of RAD001. Randomization will be stratified by one factor with three categories: (1) TSC as underlying disease and patient using enzyme-inducing anti-epileptic drugs (EIAED) at randomization, (2) TSC as underlying disease and patient not using EIAED at randomization, and (3) sporadic LAM as underlying disease. Note that patients with sporadic LAM are not expected to be using EIAED at randomization. The following drugs qualify as EIAED: phenytoin (Dylantin®, Dilantin Kapseals®, Dilantin Infatabs®, Eptoin®, Epanutin®, Diphenin®, Dipheninum®, Phenytek®), mephenytoin (Mesantoin®), carbamazepine (Tegretol®, Biston®, Calepsin®, Carbatrol®, Epitol®, Equetro®, Finlepsin®, Sirtal®, Stazepine®, Telesmin®, Teril®, Timonil®, Trimonil®, Epimaz®, and Degranol®), phenobarbital (Luminal®), pentobarbital (Nembutal®), primidone (Mysoline®), and oxcarbazepine (Trileptal®).<br>**Screening/Baseline phase**: Evaluations will be performed within 21 days prior to Treatment Day 1. If safety laboratory collections are collected more than 14 days prior to Treatment Day 1, they will need to be repeated prior to the patient's first dose of study drug. These laboratory collections that must be repeated are: hematology, biochemistry and lipid panel, coagulation, urinalysis and pregnancy testing. A CT or MRI of the kidneys should be performed for the baseline tumor assessment and all angiomyolipomata should be identified as either target (longest diameter of at least 1 cm) or non-target (longest diameter less than 1 cm). NOTE: If local or country requirements prohibit the use of CT, then only an MRI of the abdomen may be completed. In addition, polycystic kidney disease will be documented if present at baseline. The baseline kidney CT/MRI will be used to assess patient eligibility. Although the study will use an Independent Central Radiology Review, the decision to randomize the patient will be made based on the judgment of the investigator and local radiologist. Once the patient is randomized, the baseline CT/MRI of the kidneys should be sent as soon as possible by the site to the Independent Central Radiology Reviewer. There is no plan to collect radiological measurements made by the local radiologist, partly because not all sites will have the same software for measuring lesion volume, but also because all data analysis will be based on the measurements obtained from the central radiological review. All of the above assessments/procedures must be conducted prior to randomization.<br>**Blinded treatment phase/Duration of treatment**: Patients will be randomized to receive either RAD001 or matching placebo. Patients will be treated with blinded study treatment until angiomyolipoma progression (as defined in Section 7.5.2), unacceptable toxicity or discontinuation for any other reason. Dose adjustment (reduction, interruption or possible dose re-escalation to starting dose) according to safety findings will be allowed. Regular safety reviews by a Data Monitoring Committee (DMC) will be performed.<br>During the blinded treatment phase, kidney tumor assessments using CT/MRI will be performed at 12, 24 and 48 weeks after start of study treatment, and annually thereafter, until angiomyolipoma progression. The same imaging modality must be used throughout the trial. An additional CT/MRI should be performed to confirm angiomyolipoma response approximately 12 weeks (and no sooner than 8 weeks) after it was first observed. All kidney imaging conducted during the blinded phase of the trial should be sent in for central radiological review within 2 days of the scan.<br>**Open label treatment phase**: If angiomyolipoma progression (as defined in Section 7.5.2) is documented by central radiology review during the blinded treatment phase, then the treating physician may proceed to unblind the patient. CT/MRIs must be

digitized and sent to Central Radiology within 2 days of the scan date. Central radiology review of the CT/MRIs will be completed no later than 3 weeks after its receipt by the central reviewer, and so the unblinding may not be able to take place until up to 3 weeks after the progression was actually observed. However, if progression was **unequivocal** according to the local radiologist, then the investigator should contact the Novartis Clinical Trial Head (CTH) or designee to proceed with unblinding without waiting for confirmation from central radiology review.

Following unblinding, patients who had been receiving placebo may be offered open-label treatment with RAD001 if the treating physician believes the patient could benefit from this therapy.

For the open-label phase of the study, the most recent kidney CT/MRI from the blinded treatment phase of the study will be used as the baseline, and further kidney CT/MRIs will be conducted at 12, 24 and 48 weeks after start of open-label RAD001 and annually thereafter, until angiomyolipoma progression, unacceptable toxicity or discontinuation for any other reason. For each patient, the same imaging modality must continue in the open label phase of the trial as in the blinded phase. All kidney imaging obtained in the open-label phase of the trial will be sent in for central radiological review. Patients receiving open-label treatment with RAD001 will continue having safety and efficacy assessments as in the blinded treatment phase, with the exception of biomarker and pharmacokinetic assessments which will not be done.

Follow-up (for patients who discontinue study treatment): Patients who have not had angiomyolipoma progression at the time of study treatment discontinuation will be followed up with kidney CT/MRIs annually, until eventual angiomyolipoma progression, or until start of any non-study systemic anti- angiomyolipoma therapy, whichever occurs first. For each patient, the same imaging modality must be used throughout the trial. During this follow-up period, the site will continue to send imaging for central review, and use of non-study systemic anti-angiomyolipoma therapies (does not include anti-epileptic medications) will be recorded. In addition, patients will be followed for safety until 4 weeks (28 days) after study treatment discontinuation and will come in for a final follow-up visit 28 days after study treatment discontinuation. Beyond these 28 days, any serious adverse events that are suspected to be related to the study drug and occur within the next 8 weeks (56 days) as well as concomitant medications related to these events will also be collected.

Extension phase: The data cutoff date for the final analysis will be 6 months after the last patient is randomized. Once the final trial results are known, and if these results favor RAD001, then an extension phase will be launched. All patients still receiving study treatment at this time, as well as those being followed for post-treatment evaluation, will be given the option of starting open-label RAD001, which will be provided free of charge by Novartis. Those patients entering the extension phase who had previously only been receiving placebo, will have scheduled assessments beginning at Treatment Day 1 according to Table 7-2. Patients that had been on active RAD001 prior to beginning the extension phase will simply continue their sequence of assessments. The extension phase will run until 4 years after the last patient was randomized, ensuring all patients will be followed up between 4 and 5 years (assuming patient accrual over a 12-month period). Patients will be treated with open-label RAD001until the end of the extension phase, angiomyolipoma progression, unacceptable toxicity or any other reason for discontinuation. For all patients receiving RAD001 at the end of the extension phase, RAD001 will continue to be provided free of charge for as long as the medication is not commercialized and added to the list of reimbursed medication for patients with TSC.

Non-interventional follow-up phase: This phase will begin at the completion of the Extension Phase. It will continue for approximately 12 months. Patients who have not had angiomyolipoma progression at the time of Extension Phase completion and have discontinued use of everolimus will be monitored with kidney CT/MRIs in approximately 12 months, unless angiomyolipoma progresses, or the patient begins treatment with systemic anti-angiomyolipoma therapy, or undergoes embolization or nephrectomy, at which point, kidney CT/MRIs will be performed prior to the intervention and the patient will exit the study. Patients who did not have angiomyolipoma progression at the time treatment discontinuation from the extension phase and were being followed with CT/MRI tumor assessments will exit the study,

	unless the patient qualifies and elects to participate in the non-interventional follow-up phase. Patients who previously discontinued treatment with everolimus during the extension phase and have not yet taken their first annual CT/MRI may also be included in this non-interventional follow-up phase, if otherwise eligible.<br>Patients must report any angiomyolipoma-related bleeding events. Diagnostic procedure(s) pertaining to angiomyolipoma-related bleeding should also be reported. Concomitant medications and non-drug therapies given in treatment of angiomyolipoma or angiomyolipoma-related disease progression must be reported.<br>All other adverse events occuring within this one-year period will only be collected as reported by the patient for the DS&E database.All other concomitant medications and non-drug therapies will only be collected as reported by the patient for the DS&E database.<br>**End of Study:** The End of Study will take place after all patients participating in the non-interventional follow-up phase have either completed the one-year of non-interventional follow-up or have exited the study.<br>Any patient who has already completed 4 years of treatment with study medication may exit the study at any time. If a patient has already already completed 4-years of study treatment, and does not intend to continue treatment with commercially-available everolimus, the patient may begin participation in the non-interventional follow-up phase (Section 4.6). Patients who discontinue early from the extension phase and will not be treated for their angiomyolipomas may also be included in the non-interventional follow-up phase as long as their regular annual CT/MRI scan is during the non-interventional phase.
Population	Male or female patients aged 18 years or older who are diagnosed with angiomyolipoma associated with either TSC or sporadic LAM.
Inclusion/exclusion criteria	1. **Inclusion criteria**:<br>Male or female ≥ 18 years of age.<br>2. Clinically definite diagnosis of tuberous sclerosis according to the modified Gomez criteria (Roach 1998, Hyman 2000), Table 5-1 **or** sporadic LAM (biopsy-proven or compatible chest CT scan).<br>Clinically definite diagnosis of tuberous sclerosis according to the modified Gomez criteria is defined as either of the following:<br>• Two Major Features from Table 5-1.<br>• One Major Feature plus two Minor Features from Table 5-1.<br>3. Clinically definite diagnosis of renal angiomyolipoma.<br>4. Presence of at least one angiomyolipoma ≥ 3 cm in its longest diameter using CT or MRI.<br>5. If female and of child-bearing potential, documentation of negative pregnancy test prior to enrollment. Sexually active pre-menopausal female patients (and female partners of male patients) must use highly effective contraceptive measures, while on study and for 8 weeks after ending treatment.<br>6. Written informed consent according to local guidelines.<br>**Non-interventional follow-up phase/ Inclusion criteria**<br>7. Patients who do not have angiomyolipoma progression at the time of study treatment discontinuation and do not plan to continue treatment of their angiomyolipoma(s) with systemic therapy<br>8. Completion of the non-interventional follow-up phase informed consent.<br>**Exclusion criteria**:<br>1. Patients with angiomyolipomas which, in the opinion of the investigator, requires surgery at the time of randomization.<br>2. Angiomyolipoma-related bleeding or embolization during the 6 months prior to randomization.<br>3. History of myocardial infarction, angina or stroke related to atherosclerosis.<br>4. Impaired lung function, defined as any of the following:<br>For patients without lymphangioleiomyomatosis (LAM)<br>• Known impaired lung function (e.g. FEV_1 or DL_{co} ≤ 70% of predicted)

	Note: pulmonary function testing at baseline is not required for patients without LAM<br>For patients with lymphangioleiomyomatosis (LAM):<br>• DL_{CO} ≤35%, or<br>• O_2 saturations below normal at rest, or<br>• O_2 saturation ≤88% on 6 minute walking test with up to 6 liter O_2/minute nasal oxygen<br>5. Chylous ascites sufficient to affect diaphragmatic function or pulmonary function testing.<br>6. Significant hematological or hepatic abnormality (i.e., transaminase levels > 2.5 × the upper limit of normal (ULN), serum bilirubin > 1.5 × ULN, hemoglobin < 9g/dL, platelets < 80,000/mm^3, or absolute neutrophil count (ANC) < 1,000/mm^3).<br>7. Pregnancy or breast feeding.<br>8. Intercurrent infection at date of randomization.<br>9. Prior history of organ transplantation.<br>10. Recent surgery (involving entry into a body cavity or requiring sutures) within the 2 months prior to randomization.<br>11. Prior therapy with mTOR inhibitors (sirolimus, temsirolimus, everolimus).<br>12. Use of an investigational drug within the 30 days prior to randomization.<br>13. Uncontrolled hyperlipidemia: Fasting serum cholesterol > 300 mg/dL (or > 7.75 mmol/L) AND fasting triglycerides > 2.5 × ULN.<br>14. Uncontrolled diabetes mellitus as defined by fasting serum glucose > 1.5 × ULN.<br>15. Patients with bleeding diathesis or on oral anti-vitamin K medication (except low dose warfarin).<br>16. Patients with a known history of HIV seropositivity.<br>17. Inability to attend scheduled clinic visits.<br>18. For the purpose of MRI assessments:<br>• Ferromagnetic metal implants other than those approved as safe for use in MRI scanner (e.g., braces, some types of aneurysm clips, shrapnel).<br>• Patients suffering from uncontrollable claustrophobia or physically unable to fit into the machine (e.g., obesity, etc).<br>**NOTE**: patients with vagal nerve stimulators are permitted to have CT assessments of angiomyolipoma unless local or national regulations do not permit this.<br>19. Serum creatinine > 1.5 × ULN.<br>20. History of malignancy in the past two years, other than squamous or basal cell skin cancer.<br>21. Any severe and/or uncontrolled medical conditions which could cause unacceptable safety risks or compromise compliance with the protocol, such as:<br>• ≥ Grade 3 hypercholesterolemia/hypertriglyceridemia or ≥ Grade 2 hypercholesterolemia/hypertriglyceridemia with history of coronary artery disease (despite lipid-lowering treatment if given)<br>• Impairment of gastrointestinal function or gastrointestinal disease that may significantly alter the absorption of study drug (e.g., ulcerative disease, uncontrolled nausea, vomiting, diarrhea, malabsorption syndrome)<br>• Active skin, mucosa, ocular or GI disorders of Grade > 1<br>**Non interventional follow-up phase/ Exclusion criteria**<br>22. Patients who initiate treatments with systemic mTOR inhibitors such as everolimus or rapamycin<br>23. Patients who have had an embolization immediately after discontinuing study drug<br>24. Patients who have had a surgical resection of their angiomyolipoma, such as partial/complete nephrectomy immediately after discontinuing study drug.<br>25. Patients who have previously had a kidney CT/MRI performed one-year after everolimus discontinuation (during the follow-up period)
Investigational and	RAD001 or Matching Placebo (during blinded phase only)

control drugs	
Dose, regimen, treatment cycle	In both arms (RAD001 and placebo), the starting dose will be 10 mg/day.
Supply, preparation, and administration	RAD001 is formulated as 5 and 10mg tablets. For this study, tablets of 5.0 mg strength will be dosed on a daily basis. Placebo will be formulated to be indistinguishable from the RAD001 tablets. The blisters for RAD001 / placebo tablets should be opened only at the time of administration as the active drug is both hygroscopic and light-sensitive. Both RAD001 and matching placebo will be provided by Novartis and dispensed by the study center personnel. Patients who enter the open-label phase of the study will be provided with an adequate supply of RAD001.<br>Patients will be instructed to take two 5.0 mg tablets of RAD001 or matching placebo orally with a glass of water once daily at the same time each day. RAD001 or matching placebo should be administered immediately after a meal. Any dietary habits around the time of RAD001 or matching placebo intake should be as consistent as possible throughout the study, and in particular, during those periods when samples are being taken for pharmacokinetic analyses. If vomiting occurs, no attempt should be made to replace the vomited dose.<br>At visits when blood will be drawn, patients should **not** take the daily study drug dose **until after** blood is drawn so that an accurate trough level of RAD001 can be obtained. At these visits, patients should bring their daily dose of medication to the clinic with them for administration by investigator (or designee) after the blood work is completed.<br>Patients will receive treatment with study drug until angiomyolipoma progression, unacceptable toxicity, or until the investigator or patient decides that continuation is not in the best interest of the patient. For patients who are unable to tolerate the protocol-specified dosing schedule, dose adjustments are permitted in order to keep the patient on study drug. The guidelines set forth in Table 6-1, Table 6-2 and Table 6-3 should be followed.
Visit schedule and assessments	Refer to Table 7-1,Table 7-2 and Table 7-3 Non-interventional follow-up phase visit evaluation schedule
Efficacy assessment(s)	A CT/MRI of the kidneys will be performed at screening/baseline, at 12, 24 and 48 weeks after start of study treatment, and annually thereafter. The same imaging modality should be used throughout the trial. All imaging scans of the kidney will be submitted for an Independent Central Radiology Review.<br>Investigators should ensure the safety of patients with vagal nerve stimulators by following applicable guidance (e.g. http://.fda.gov/MedicalDevices/Safety/AlertsandNotices/PublicHealthNotifications/ucm062125.htm).<br>For patients without LAM, no PFTs will need to be performed at screening/baseline. For patients with LAM, PFT data will be available from routine testing at baseline, 6, 12, 18 and 24 weeks and then every 12 weeks thereafter on study.<br>Plasma angiogenesis markers (e.g., VEGF, basic FGF, PLGF, soluble VEGF receptor1 and soluble VEGF receptor2) will be measured at screening/baseline, at 4, 12, 24, 36 and 48 weeks after start of treatment, and at end of treatment to measure changes while on treatment and capture any change in angiogenic markers at end of treatment.<br>Skin lesions, for patients with documented skin lesions at baseline, will be photographed using a digital camera at baseline and every 12 weeks thereafter, and assessed using the Physician's Global Assessment of Clinical Condition after every 12 weeks of treatment.
Safety assessments	Toxicity will be assessed by the NCI CTCAE v3.0 (http://ctep.cancer.gov/forms/CTCAEv3.pdf). The NCI CTCAE has been chosen in order to be consistent with other RAD001 trials. Safety assessments will include monitoring and recording of all AEs and SAEs at every visit, and monitoring of vital signs, physical condition and body weight. Laboratory assessments including hematology and blood chemistry (including serum creatinine and calculated creatinine clearance) will be done every two weeks for the first 8 weeks, at 12, 18 and 24 weeks and every 12 weeks thereafter.<br>Patient should report any angiomyolipoma-related bleeding events to the site.

	Diagnostic procedure(s) pertaining to angiomyolipoma-related bleeding should also be reported. Concomitant medications and non-drug therapies given in treatment of angiomyolipoma or angiomyolipoma-related disease progression must be reported.<br>All other adverse events occurring during the non-interventional follow-up period will be collected, as reported by the patient for the DS&E database. All other concomitant medications and non-drug therapies will only be collected as reported by the patient for the DS&E database.<br>**Hepatitis Screening**<br>Prior to randomization, the following three categories of patients should be tested for hepatitis B viral load and serologic markers, that is, HBV-DNA, HBsAg, HBs Ab, and HBc Ab:<br>All patients who currently live in (or have lived in) Asia, Africa, Central and South America, Eastern Europe, Spain, Portugal, and Greece. [http://nc.cdc.gov/travel/yellowbook/2010/chapter-2/hepatitis-b.aspx#849]<br>Patients with any of the following risk factors:<br>• known or suspected past hepatitis B infection,<br>• blood transfusion(s) prior to 1990,<br>• current or prior IV drug users,<br>• current or prior dialysis,<br>• household contact with hepatitis B infected patient(s),<br>• current or prior high-risk sexual activity,<br>• body piercing or tattoos,<br>• mother known to have hepatitis B<br>• history suggestive of hepatitis B infection, e.g., dark urine, jaundice, right upper quadrant pain.<br>Additional patients at the discretion of the investigator<br>If a patient tests positive, he/she will be considered ineligible for the study according to Exclusion Criterion 8. Please note that patients who test negative for HBV-DNA, HBsAg, and HBc Ab but positive for HBs Ab with prior history of vaccination against Hepatitis B will be eligible. The fact that the patient had been vaccinated should be entered into the patient’s Medical History CRF.<br>Patients with any of the following risk factors for hepatitis C should be tested using quantitative RNA-PCR<br>• known or suspected past hepatitis C infection (including patients with past interferon ‘curative’ treatment),<br>• blood transfusions prior to 1990,<br>• current or prior IV drug users,<br>• current or prior dialysis,<br>• household contact of hepatitis C infected patient(s),<br>• current or prior high-risk sexual activity,<br>• body piercing or tattoos<br>At the discretion of the investigator, additional patients may also be tested for hepatitis C.<br>If a patient tests positive, he/she will be considered ineligible for the study according to Exclusion Criterion 8.<br>For patients who have already been randomized and received study drug prior to the approval of Amendment 1, the same screening process should be followed at the patient's next visit.<br>If the patient tests positive for Hepatitis B, the investigator should follow the guidelines according to Table 4-1 and Table 4-2.<br>Please refer to Table 7-1 and Table 7-2 for HCV RNA-PCR monitoring schedule for those patients with positive HCV RNA-PCR baseline tests who do not meet the reactivation criteria outlined in Table 4-3. If the patient tests positive for hepatitis C, and the criteria for reactivation according to Table 4-3 are observed, trial therapy should be discontinued and further treatment is up to the investigators discretion.

	All patients will have additional blood samples collected during screening and every 12 weeks for endocrine assessment. These tests include:<br>• Follicle Stimulating Hormone (FSH)<br>• Luteinizing Hormone (LH)<br>• Testosterone<br>• Estradiol (female patients)<br>Patients will be followed for safety until 4 weeks (28 days) after study treatment discontinuation. Beyond these 28 days, any serious adverse events that are suspected to be related to the study drug and occur within the next 8 weeks (56 days) will also be collected.<br>Pneumonitis is a known side-effect of rapamycin analogues including RAD001. Clinically significant pneumonitis is typically accompanied by non-specific symptoms including dyspnea, nonproductive cough, fatigue, and fever. Diagnosis is generally suspected in individuals receiving mTOR inhibitors who develop these symptoms or in asymptomatic individuals in whom a routine chest CT scan reveals a new ground glass or alveolar infiltrate. The frequency of symptomatic pulmonary toxicity (all grades) was approximately 14% in a phase III study of RAD001 in patients with metastatic renal cell carcinoma ([CRAD001C2240]). Severe (CTC grade 3) pneumonitis occurred in 4% of patients, and an occasional fatality was reported. The lung toxicity was partly or completely reversible in the majority of cases with interventions including drug interruption, discontinuation and the use of corticosteroids.<br>Baseline lung function will be assessed via pulmonary function testing in all LAM patients. Individuals participating in this trial will be questioned at each visit as to the presence of new or changed pulmonary symptoms consistent with drug-induced lung toxicity. If an investigator suspects a patient may be developing pneumonitis investigations such as pulmonary function tests, chest CT and referral to a pulmonologist should be considered. In addition, individuals with LAM will undergo routine pulmonary function testing at 6, 12, 18 and 24 weeks and every 12 weeks thereafter during their study visits.<br>Pulmonary function studies will be performed and interpreted using the ATS/ERS guidelines (Brusasco et al 2005).
Potential drug interactions	Patients must be instructed not to take any additional medications (over-the-counter or other products) during the study without prior consultation with the investigator. All medications taken within 30 days of starting study treatment should be reported on the Concomitant Medication/Significant Non-drug Therapies CRF. **With the exception of enzyme-inducing anti-epileptic drugs, which are allowed on this study, the following guidelines must be adhered to during the study**:<br>Investigational or commercial anti-proliferative agents other than study drug (including other mTOR inhibitors, e.g., sirolimus, temsirolimus) are prohibited.<br>**Inhibitors of CYP3A4 and/or PgP**<br>• Co-administration with strong inhibitors of CYP3A4 (e.g., ketoconazole, itraconazole, ritonavir) or P-glycoprotein (PgP) should be avoided.<br>• Co-administration with moderate CYP3A4 inhibitors (e.g., erythromycin, fluconazole) or PgP inhibitors should be used with caution. If patient requires co-administration of moderate CYP3A4 inhibitors or PgP inhibitors, reduce the dose of everolimus to half the currently used dose. Additional dose reductions to every other day may be required to manage toxicities. If the inhibitor is discontinued the everolimus dose should be returned to the dose used prior to initiation of the moderate CYP3A4/PgP inhibitor.<br>• Seville orange, star fruit, grapefruit and their juices affect P450 and PgP activity. Concomitant use should be avoided.<br>**Inducers of CYP3A4 and/or PgP**<br>• Avoid the use of strong CYP3A4 inducers. If patient requires co-administration of strong CYP3A4 inducers (i.e., phenytoin, carbamazepine, rifampin, rifabutin, phenobarbital, St. John's wort), an increase in the dose of everolimus up to twice the currently used daily dose should be considered, using 5mg increments. Enzyme induction usually occurs within 7-10 days, therefore everolimus dose should be increased by one increment 7 days after the start of the inducer

	therapy. If no safety concerns are seen within the next 7 days, the dose can be increased again one additional increment up to a maximum of twice the daily dose used prior to initiation of the strong CYP3A4 inducer.<br>• This dose adjustment of everolimus is intended to achieve similar AUC to the range observed without inducers. However, there are no clinical data with this dose adjustment in patients receiving strong CYP3A4 inducers. If the strong inducer is discontinued the everolimus dose should be returned to the dose used prior to initiation of the strong CYP3A4/PgP inducer.<br>A list of medications with known effects on CYP3A and PgP are included in Table 6-5.<br>RAD001 may affect the response to vaccinations making the response to the vaccination less effective. Live vaccines and close contact with those who have received live vaccines should be avoided while a patient is treated with RAD001.<br>Otherwise, the use of other concomitant medication/therapy deemed necessary for the care of the patient is allowed. The investigator should instruct the patient to notify the study site about any new medications he/she takes after the start of the study drug. All medications (other than study drug) and significant non-drug therapies (including physical therapy and blood transfusions) administered after the patient starts trial therapy must be listed on the Concomitant Medications/Significant Non-drug Therapies CRF.
Pharmacokinetics	A 2-mL pre-dose blood sample will be collected for determination of RAD001 trough blood levels (C_{min}) at weeks 2, 4, 12, 24 and 48. In addition, at each of these visits, an additional 2-mL blood sample will be collected for determination of PK 2.0 hours (±30 mins, C_{2h}) after trial therapy dose administration.
Biomarker/genetic assessments	Plasma collection for biomarker assessments should occur at the following time points: at screening/baseline, at 4, 12, 24, 36 and 48 weeks and at end of treatment. Plasma samples (3 mL of blood) will be examined for RAD001 effects on angiogenesis markers (e.g., basic FGF, VEGF, PLGF, soluble VEGF receptor1, and soluble VEGF receptor2). At screening/baseline, a 3-mL pre-dose sample of blood will be collected for determination of mutations associated with TSC1 and TSC2 genes.
Optional Biomarker studies on additional or remaining samples	Retention samples will be used only to investigate new genes or proteins that are discovered to be associated with Tuberous Sclerosis disease or Lymphangioleiomyomatosis.
DMC/Study Steering Committee	A Data Monitoring Committee (DMC) will be constituted to oversee the safety of patients on this trial; the same DMC will be used in any other Novartis-sponsored trials evaluating RAD001 in patients with TSC-related diseases. The DMC will consist of at least two clinicians with expertise in TSC and one statistician. The DMC will be constituted prior to the randomization of the first patient in any of the Novartis-sponsored trials of RAD001 in TSC. The first safety review will be performed by the DMC approximately six months after the first patient in any of the Novartis-sponsored RAD001 trials in TSC has been randomized, and every six months thereafter, unless otherwise requested by the Chairman of the DMC. No interim analysis is planned. Recruitment will not be interrupted unless otherwise requested by the Chairman of the DMC. In addition, the DMC will receive safety updates on a regular basis.<br>A Study Steering Committee (SSC) will also be constituted to oversee the conduct of the study and make any necessary recommendations as appropriate. The committee will also develop study-related publications in accordance with the Novartis publication and authorship policy. The committee will be appointed by Novartis and will include two principal investigators from this trial, Novartis staff and possibly other clinical experts (after consultation with the SSC members). The committee will be chaired by one of the two Principal Investigators.
Statistical methods and data analysis	**Analysis Populations**<br>• The Full Analysis Set (FAS) will consist of all randomized patients. The patients in the FAS will be analyzed in the treatment group they were assigned to at randomization.<br>• The Per Protocol Set (PPS) will consist of all patients from the FAS without any major protocol deviation, who are evaluable for efficacy and who have completed a minimum exposure requirement. However, if a patient progressed, discontinued due to an adverse event or died before the minimum exposure requirement could

	be met, or before he/she could become evaluable for efficacy, that patient will still be included in the PPS. Patients will be evaluable for efficacy if they have a known angiomyolipoma response status. The minimum exposure requirement is defined as having a relativedose intensity over the first 12 weeks of treatment of at least 50%. The PPS will be used in a supportive analysis of the primary endpoint.<br>• The safety population will consist of all patients that received at least one dose of the study medication and had at least one post-baseline safety assessment. Patients will be analyzed according to treatment received. Note: The statement that a patient had no adverse events (on the Adverse Events CRF) constitutes a safety assessment.<br>**Primary analysis**<br>The primary analysis will be a comparison of the angiomyolipoma response rates in the RAD001 and placebo arms using an exact Cochran-Mantel-Haenszel (CMH) test at the one-sided 2.5% level, stratified by the protocol stratification factor, analyzed in the FAS. The analysis will be performed using a data cut-off defined as 6 months after the last patient is randomized in the trial.<br>**Interim analysis**<br>Not planned.<br>**Statistical considerations**<br>Angiomyolipomata are slow growing lesions. Spontaneous regression without surgical or drug intervention has not been reported. Reduction in volume, relative to baseline assessment, will be considered treatment-related. As there is no approved treatment for angiomyolipoma, a placebo-controlled randomized design is a logical choice for evaluating a new treatment.<br>Because of their slow progressing nature, response rate is an ideal outcome parameter. CT/MRI will be used to evaluate changes in tumor dimensions because of the high accuracy of measurement and spatial resolution and the absence of radiation associated with the technique.<br>**Statistical power and rationale for sample size**<br>The primary analysis compares angiomyolipoma response rate between the two treatment arms using an exact CMH test. The randomization is unbalanced, with two patients allocated to RAD001 for every one patient allocated to placebo. As there are no reported cases of tumor regression in patients with angiomyolipoma, the response rate on placebo is expected to be close to 0%. The angiomyolipoma response rate on RAD001 is anticipated to be at least 20%. It is planned to use a one-sided test and a 2.5% significance level. Simulation was used to obtain a sample size of 99 patients (66 randomized to RAD001 and 33 randomized to placebo), which will provide 93% power to detect a treatment difference from 0% on placebo to 20% on RAD001.<br>**Pharmacokinetic analyses**<br>RAD001 trough (C_{min}) and 2.0 hour (±30 mins, C_{2h}) levels will be summarized by means of descriptive statistics and used in future analyses along with RAD001 trough levels and steady-state profiles from other studies, with the goal to compare the results from this protocol with an appropriate reference population (e.g., PK data from other RAD001 monotherapy protocols in patients with TSC-related conditions).

1 Background

1.1 Overview of Tuberous Sclerosis Complex (TSC) and Lymphangioleiomyomatosis (LAM)

Tuberous Sclerosis Complex (TSC) is an autosomal dominant genetic disorder caused by inactivating mutations in the tuberous sclerosis complex tumor suppressor genes, *TSC1* or *TSC2*, affecting tuberin and hamartin respectively. The results of second somatic mutation in the heterozygous background include benign, highly vascular, hamartomatous growths. Lesions occur in the brain, kidneys, heart, liver, lungs and skin, and phenotypically can manifest with renal and/or pulmonary complications, autism, mental retardation and epilepsy (Gomez 1999, Astrinidis 2005, Inoki 2005, Kwiatkowski 2005).

Lymphangioleiomyomatosis (LAM) is a rare disorder related to abnormal function of the tuberous sclerosis genes 1 and 2 (TSC1 and TSC2). Mutations in the *TSC2* gene generally are thought to be the cause of sporadic LAM (that is, in patients with no evidence of TSC) (Smolarek 1998, Carsillo 2000). Although LAM has been recognized more as a sporadic, noninheritable pulmonary disorder, it also occurs in about one third of women with TSC (Costello 2000, Franz 2001, Moss 2001). In patients with TSC, LAM predominantly affects premenopausal women and is very rare in men (Hancock et al 2002).

Normal lung is replaced by numerous cysts and histologically there is diffuse proliferation of smooth muscle-like cells in the remaining alveolar septa. Sporadic LAM is a relatively uncommon disease with a prevalence that has been estimated at 2.6 per 1 million women (Urban et al 1999). However, the disease progression in TSC-associated LAM may be different from the sporadic form as only approximately 1% of women with TSC develop clinical symptoms (Hancock et al 2002).

Data suggest that LAM cells can metastasize; identical mutations in *TSC2* were found in lung lesions and angiomyolipomata from the same patient with sporadic LAM, and recurrent LAM cells of recipient origin were detected in the donor lung of a transplanted patient (Maruyama 2001, Bittmann 2003, Karbowniczek 2003). Although the “primary” source of LAM cells in the lungs is unknown, the potential sources include angiomyolipomata and the lymphatic system (Henske 2003).

Approximately 60% of patients with sporadic LAM also have an angiomyolipoma (Bernstein and Robbins 1991). The angiomyolipoma histopathologic features seen in sporadic LAM are identical with those found in TSC (Chan et al 2004). Both TSC associated and sporadic LAM are characterized by smooth muscle infiltration into the walls of the alveoli and small airways. This can lead to cystic degeneration of lung tissue, impaired gas exchange, respiratory failure, and death. Progressive symptomatic lung disease may occur in patients diagnosed with sporadic LAM and such progression likewise would be predicted to occur in women with TSC-associated LAM (Bissler and Kingswood 2004). Eventual respiratory failure due to destruction of lung tissue may require lung transplantation, which remains the most viable option for end-stage disease.

A compelling argument can be made for treatment with pharmacological mTOR inhibitors such as everolimus (RAD001). Loss of TSC1and TSC2 leads to increased mTOR activity and

downstream activation of S6-kinase-dependent gene expression, including the lymphangiogenic factor VEGF-D. A study of sirolimus in individuals with angiomyolipomata and LAM demonstrated a remarkable increase in airflow in women treated with sirolimus for up to 12 months (Bissler et al 2008).

1.2 Overview of Angiomyolipomata

Angiomyolipomata are the most common renal manifestation of TSC (Gomez et al 1999) developing during later childhood and adolescence. As the name implies, they are composed of blood vessels, smooth muscle and adipose tissue. The majority of adults affected by the disease have multiple bilateral lesions which are usually asymptomatic but can cause life-threatening hemorrhage or impaired renal function. Angiomyolipomata can occur in the liver but these are rarely of clinical significance (Yates 2006). Renal cysts are also a common finding (Gomez et al 1999). They are usually asymptomatic except in the rare case of patients with both TSC and polycystic kidney disease owing to contiguous deletions of the *TSC2* and PKD1 genes who usually present with severe early onset renal cystic disease and progress to end-stage renal failure by early adult life (Sampson et al 1997). Patients with TSC appear to be at increased risk of renal cell carcinoma, estimated at 1-3% (Nelson and Sanda 2002).

There are two morbidities associated with renal angiomyolipomata. The first and more dramatic is Wunderlich syndrome (Chesa Ponce et al 1995), a retro-peritoneal hemorrhage originating in the angiomyolipoma. As they enlarge, angiomyolipomata frequently develop both micro- and macro-aneurysms which can rupture (Adler 1984, Bissler 2002). Patients with this sudden, painful, and often life-threatening event are most often first seen in the emergency department (Bissler and Kingswood 2004). It was estimated that up to 20% of patients with such hemorrhages present in shock (Pode et al 1985). With such a presentation, the treatment may be a total nephrectomy, and this approach complicates the patient's long-term care. Angiomyolipomata associated with TSC are usually bilateral and a nephrectomy would result in a significant loss of functional renal tissue, thus hastening the need for renal replacement therapy (Bissler and Kingswood 2004). A population study suggests that the cumulative risk of a hemorrhage is 18% for females and 8% for males (Webb et al 1994). However, among a clinic population of approximately 310 adult and pediatric TSC patients in Cincinnati, only nine cases of hemorrhage (3%) were observed (Bissler and Kingswood 2004). This difference may be, at least in part, due to an aggressive embolization program, population age differences or length of follow-up. Angiomyolipomata appear to grow over time, and there is an association between lesion size and hemorrhage (Steiner 1993, Dickinson 1998).

The second morbidity of renal angiomyolipomata is the insidious encroachment of the angiomyolipoma on normal renal tissue, which may lead to renal failure (Schillinger 1996, Clarke 1999). The precise incidence of end-stage renal disease (ESRD) in the tuberous sclerosis population has not been well defined. However, European surveys suggest that approximately 1% of the TSC patient population with normal intellect is receiving dialytic renal replacement therapy (Schillinger 1996, Clarke 1999), leading to an estimate that over 30,000 TSC patients are on dialysis worldwide. Shepherd et al examined death certificates ofpatients in their TSC clinic and found that of 355 patients, 40 died as a result of their TSC, most commonly due to renal failure (Shepherd et al 1991). The percentage of TSC patients developing ESRD because of polycystic kidney disease, multiple interventions for

hemorrhage, or replacement of renal tissue by angiomyolipomata is unclear (Bissler and Kingswood 2004).

Approximately 35% of patients with TSC will have renal cysts (Cook et al 1996). Although these lesions may be large, they usually are not numerous and rarely cause problems (Bissler and Kingswood 2004).

The primary reason to intervene in patients with renal angiomyolipomata has been to alleviate symptoms such as pain or hemorrhage. The recent urological literature has embraced a renal sparing approach for angiomyolipomata (Nelson and Sanda 2002). Key to the long-term outcome of patients with multiple renal angiomyolipomata is the preservation of renal function. Indications for a total nephrectomy are limited and include a non-functioning kidney resulting in uncontrolled hypertension, local tissue invasion, tumor in the renal vein, or very strong evidence of malignancy (Bissler and Kingswood 2004). Partial or nephron-sparing nephrectomies run the risk of significant hemorrhage, and therefore should be undertaken only if unequivocally indicated (Bissler and Kingswood 2004). Embolization is currently regarded as a suitable alternative to such invasive procedures. The procedure obliterates the blood supply to the angiomyolipoma and thus reduces the risk of hemorrhage. Nevertheless, embolization also has significant side effects. In a review of published series, it was estimated that 85% of patients develop post-embolization syndrome, including significant fever and pain (Bissler and Kingswood 2004). In addition, although embolization and surgical therapies can successfully treat solitary lesions, the much more vexing clinical problem of coalescent renal angiomyolipomata that replace renal parenchyma has remained largely unaddressed. When bleeding occurs in this circumstance, it can be impossible to identify which lesion is the source.

Finally, (Bissler et al 2008) reported on a 24-month, phase II, non-randomized trial that evaluated the efficacy and safety of sirolimus in a similar patient population. All patients had at least one angiomyolipoma 1 cm or more in the largest dimension. Sirolimus was administered for the first 12 months only. Serial MRI of angiomyolipomata and brain lesions, CT scans of lung cysts, and pulmonary-function tests (PFTs) were performed. Of the 25 patients enrolled, 20 completed the 12-month evaluation, and 18 completed the 24-month evaluation. The mean (±SD) angiomyolipoma volume at 12 months was 53.2±26.6% of the baseline value (P<0.001) and at 24 months was 85.9±28.5% of the baseline value (P = 0.005). At 24 months, five patients had a persistent reduction in the angiomyolipoma volume of 30% or more. During the period of sirolimus therapy, among patients with LAM, the mean forced expiratory volume in 1 second (FEV1) increased by 118±330 ml (P = 0.06), the forced vital capacity (FVC) increased by 390±570 ml (P<0.001), and the residual volume decreased by 439±493 ml (P = 0.02), as compared with baseline values. One year after sirolimus was discontinued, the FEV_1 was 62±411 ml above the baseline value, the FVC was 346±712 ml above the baseline value, and the residual volume was 333±570 ml below the baseline value; cortical tubers and subependymal nodules were unchanged. None of the patients had SEGAs. Five patients had six serious adverse events while receiving sirolimus, including diarrhea, pyelonephritis, stomatitis, and respiratory infections. The authors concluded that suppression of mTOR signaling might constitute an ameliorative treatment in patients with TSC or sporadic LAM (Bissler et al 2008).

1.2.1 Choice of a minimum angiomyolipoma size

Angiomyolipomata increase in both number and size with age. One single institutional study of 60 patients showed that at an average age of 6.9 years, approximately 40% of children had angiomyolipomata; by an average age of 10.5 years, the figure had increased to 60% (Ewalt et al 1998). This study concluded that the average age at which renal abnormalities first occur was 7.2 years. Other studies suggest that the average age of first detectable renal abnormality is slightly higher at 9.2 or 11.1 years (Casper 2002, Rakowski 2006). The literature also suggests a positive correlation between patient age and tumor size. A study with an average patient age of 11.4 years had a mean lesion size of 2.1 cm (Casper et al 2002), whereas a study whose population was 32 years old on average, had a larger mean tumor size of 8.2 cm (Steiner et al 1993). One study reported that only post-pubertal patients had lesions of greater than 4 cm (Ewalt et al 1998).

Angiomyolipomata appear to grow over time, and there is an association between lesion size and hemorrhage (Steiner 1993, Van Baal 1994, Oesterling 1986, Dickinson 1998).

Current clinical practice varies between a watchful waiting policy to an aggressive embolization program. Patients eligible for the current trial should have at least one angiomyolipoma 3 cm or more in the largest dimension. The choice of 3 cm, made after a wide consultation process, reflects the vague limits between a justified wait and watch policy and the more proactive surgical intervention approaches.

1.3 Volumetric assessment of tumor response

Assessment of tumor response to therapy is a critical component for drug development and for the clinical management of patients. Current approaches for classifying tumor response are based on anatomical measurements in either one dimension (Response Evaluation Criteria in Solid Tumors [RECIST] (Therasse et al 2000)) or two dimensions (World Health Organization [WHO] (Miller et al 1981)). However, response rates as determined from these criteria may not always be sufficiently accurate.

As tumors grow in three dimensions, shrinkage can thus be accurately defined as a decrease in tumor volume. RECIST and WHO measurements are essentially surrogates for volume. With developing state-of-the-art imaging techniques providing a 3-D information set and computer algorithm development, it is now possible to obtain accurate and true tumor measurements using volume (Twombly 2006), rather than only one or two dimensional measurements. Also, for the changes in uni- and bi-dimensional measurements to be a good surrogate for changes in volume, one should assume that target lesions are spherical in shape, which may not be true for all tumor types.

Recently, Mayr et al (2006) used serial MRI examinations of 60 patients with cervical cancer to evaluate tumor shape and temporal change during radiation therapy and analyze the effect of tumor conformational changes on the correlation between region of interest-based and diameter-based MRI tumor measurement (Mayr et al 2006). The researchers reported that most cervical cancers (70%) are not oval in shape and they become increasingly irregular during and after therapy because of nonconcentric tumor shrinkage. The authors concluded that three-dimensional volumetry, which can optimally measure irregular volumes, may provide better response assessment during treatment than diameter-based measurement.

A recent breast cancer study assessed the value of MRI measurements of breast tumor volume for predicting recurrence-free survival (RFS) in patients undergoing neoadjuvant chemotherapy and compared the predictive value of MRI assessments with that of established prognostic indicators (Partridge et al 2005). This study included 62 patients and the longest diameter and volume of each tumor were measured on MRI before and after each cycle of chemotherapy. Cox model analysis showed initial MRI volume was the strongest predictor of RFS (p = 0.002). Final change in MRI volume (p = 0.015) was more predictive than change in diameter on MRI (p = 0.077) or clinical examination (p = 0.27). Multivariate analysis showed initial MRI volume (p = 0.005) and final change in MRI volume (p = 0.003) were significant independent predictors. The authors concluded that MRI tumor volume was more predictive of RFS than tumor diameter, suggesting that volumetric changes measured using MRI may provide a more sensitive assessment of treatment efficacy.

Finally, in a public workshop on brain tumor clinical trial endpoints organized by the FDA, AACR and ASCO on January 20, 2006, it was recognized that measuring tumor diameter is probably an outdated methodology, as small percentage changes in diameter can reflect much larger changes in tumor volume. It was also mentioned that both manual and automated segmentation techniques provide more accurate measurements of tumor volume than diameter measurement. In addition, the regional distribution of the lesion was considered an important issue; a 1 mm^3 reduction in tumor volume in a certain part of the brain might have a dramatic clinical effect whereas a larger volume reduction elsewhere in the brain might be clinically meaningless.

1.4 Overview of RAD001

1.4.1 RAD001

RAD001 (everolimus) is a novel derivative of rapamycin. RAD001 has been in clinical development since 1996 as an immunosuppressant in solid organ transplantation. Since 2003, RAD001 is approved in Europe (trade name: Certican®), via the Mutual Recognition Procedure for the prevention of organ rejection in patients with renal and cardiac transplantation. Approval has been obtained in > 65 countries worldwide since 2003. In the United States, Certican® remains an investigational new drug (IND No. 52,003) and approval is pending.

RAD001 is being investigated as an anti-cancer agent based on its potential to act:

- directly on the tumor cells by inhibiting tumor cell growth and proliferation
- indirectly by inhibiting angiogenesis leading to reduced tumor vascularity (via potent inhibition of tumor cell VEGF production and VEGF-induced proliferation of endothelial cells)

Everolimus (Afinitor®) entered clinical development for numerous oncology indications in 2002. Afinitor was granted approval in the United States on 30-Mar-2009 for the treatment of patients with advanced renal cell carcinoma (RCC) after failure of treatment with sunitinib or sorafenib. Afinitor was approved by the European Commission on 03-Aug-2009. As of 11-Dec-2009, Afinitor is also approved in Argentina, Australia, Brazil, Chile, Costa Rica, Hungary, Iceland, India, Malaysia, New Zealand, Norway, South Korea, Switzerland, and Venezuela. Applications are pending in various other countries worldwide.

1.4.2 mTOR pathway and tumorigenesis

An important aspect of the antitumor effect of RAD001 is its potential to act on both tumor cells directly (by inhibiting growth) and indirectly (by inhibiting angiogenesis). The observation of *in vivo* sensitivity of xenografts comprised of cells demonstrating resistance to RAD001 *in vitro* is attributed to the drug's potential to act on the vascular component of the supporting peritumoral stroma. The antiangiogenic property of RAD001 has been confirmed through experiments demonstrating the effect of RAD001 in countering VEGF-induced proliferation of human umbilical vein endothelial cells (HUVECs) *in vitro*, VEGF-driven angiogenesis in a chamber implant murine model and neovascularization in a murine orthotopic melanoma model.

At the cellular and molecular level, RAD001 acts as a signal transduction inhibitor. RAD001 selectively inhibits mTOR which regulates cell growth, proliferation and survival. The mTOR kinase is mainly activated via the phosphatidylinositol 3-kinase (PI3K) pathway through AKT/PKB and the tuberous sclerosis complex (TSC1and TSC2). Mutations in these components or in PTEN, a negative regulator of PI3 kinase, may result in their dysregulation. Abnormal functioning of various components of the signaling pathways contributes to the pathophysiology of numerous human cancers. Various preclinical models have confirmed the role of this pathway in tumor development (Cohen et al 2005).

The main known functions of mTOR include (Bjornsti and Houghton 2004):

- mTOR functions as a sensor of mitogens, growth factors, energy and nutrient levels, facilitating cell-cycle progression from G1 - S phase in appropriate growth conditions.
- The PI3K (mTOR) pathway itself is frequently deregulated in many human cancers, and oncogenic transformation may sensitize tumor cells to mTOR inhibitors.
- The mTOR pathway is involved in the production of pro-angiogenic factors (e.g., VEGF) and endothelial cell growth and proliferation.
- Through inactivating eukaryotic initiation factor 4E binding proteins and activating the 40S ribosomal S6 kinases (e.g., p70S6K1), mTOR regulates protein translation.

The regulation of mTOR signaling is complex and involves positive regulators, such as AKT, that phosphorylate and inactivate negative regulators such as the Tuberous Sclerosis Complex (*TSC1/TSC2*).

The PI3K/AKT/mTOR pathway is known to be dysregulated in numerous proliferative disorders including cancer. Molecular epidemiological studies have also shown that activation of the PI3K/AKT/mTOR pathway is frequently associated with worsening prognosis through resistance to treatment, disease extension and disease progression. A variety of preclinical models have confirmed the role of this pathway in tumor development. It has also been demonstrated that constitutional activation of kinases such as AKT can lead to inexorable development of cancers resembling those characterized by frequent activation of the same kinases. This is complemented by the demonstration of the antitumor activity of kinase inhibitors acting on the pathway in *in vitro* and *in vivo* preclinical models.

Table 1-1 Drug substance

Chemical name:	(1R,9S,12S,15R,16E,18R,19R,21R,23S,24E,26E,28E,30S,32S,35R)-1,18-dihydroxy-12-(1R)-2-[(1S,3R,4R)-4-(2-hydroxyethoxy)-3-methoxycyclohexyl]-1-methylethyl}-19,30-dimethoxy-15,17,21,23,29,35-hexamethyl-11,36-dioxa-4-aza-tricyclo[30.3.1.0^{4,9}]hexatriaconta-16,24, 26,28-tetraene-2,3,10,14,20-pentaone
International non-proprietary name	Everolimus

1.4.3 Preclinical studies

RAD001 inhibits the proliferation of a range of human tumor cell lines *in vitro* including lines originating from lung, breast, prostate, colon, melanoma and glioblastoma. RAD001 also inhibits the proliferation of HUVECs *in vitro*, with particular potency against VEGF-induced proliferation suggesting that RAD001 may also act as an anti-angiogenic agent. The anti-angiogenic activity of RAD001 was confirmed *in vivo*. RAD001 selectively inhibited VEGF-dependent angiogenic response at well tolerated doses. Mice with primary and metastatic tumors treated with RAD001 showed a significant reduction in blood vessel density when compared to controls.

RAD001 administered daily p.o. was a potent inhibitor of tumor growth, at well tolerated doses, in 11 different mouse xenograft models (including pancreatic, colon, epidermoid, lung and melanoma) and two syngeneic models (rat pancreatic and mouse orthotopic melanoma). These models included tumor lines considered sensitive and “relatively resistant” *in vitro*. In general, RAD001 was better tolerated in mouse xenograft models than standard cytotoxic agents (i.e., doxorubicin and 5-fluorouracil), while possessing similar anti-tumor activity. Additionally, activity in a VEGF-impregnated s.c. implant model of angiogenesis and reduced vascularity (vessel density) of RAD001-treated tumors (murine melanoma) provided evidence of *in vivo* effects of angiogenesis.

In vivo studies investigating the antitumor activity of RAD001 against experimental animal tumor models showed that RAD001 monotherapy typically reduced tumor cell growth rates rather than producing regressions or stable disease. These effects occurred within the dose range of 2.5 to 10 mg/kg p.o. daily.

All significant adverse events observed in toxicology studies with RAD001 in mice, rats, monkeys and mini-pigs were consistent with its anticipated pharmacological action as an anti-proliferative and immunosuppressant and at least in part reversible after a 2- or 4-week recovery period with the exception of the changes in male reproductive organs, most notably testes.

1.4.4 RAD001 pharmacokinetics

The pharmacokinetic characteristics of RAD001 have been extensively investigated in the context of the drug’s development as an immunosuppressant in solid organ transplantation where RAD001 was administered twice daily as a part of an immunosuppressant, multi-drug regimen consistently including cyclosporine A and glucocorticoids. Recent phase I studies provide steady-state pharmacokinetics for both the weekly and daily schedules at varying dose levels in patients with advanced cancers.

RAD001 is rapidly absorbed after oral administration, with a median time to peak blood levels (t_{max}) of 1-2 hours postdose. The extent of absorption is estimated at above 11%. The area under the blood concentration-time curve (AUC) is dose-proportional over the dose range tested while maximum blood concentration (C_{max}) appears to plateau at dose levels higher than 20 mg. The terminal half-life in cancer patients averaged 30 hours, similar to that in healthy subjects. Inter-patient variability is moderate with a coefficient of variation (CV) of approximately 50%. In healthy subjects, high fat meals reduced systemic exposure to Afinitor 10 mg (as measured by AUC) by 22% and the peak plasma concentration C_{max} by 54%. Light fat meals reduced AUC by 32% and C_{max} by 42%. Food, however, had no apparent effect on the post absorption phase concentration-time profile. A high-fat meal altered the absorption of RAD001 with a 1.3-hour delay in t_{max}, a 60% reduction in C_{max} and a 16% reduction in AUC. In whole blood, approximately 80% of RAD001 is contained in red blood cells. Of the fraction of drug contained in plasma, 74% is protein-bound. The apparent distribution volume after a single dose was 4.7 L/kg. RAD001 is eliminated by metabolism, mainly by hydroxylation, then excreted into the feces at >80%.

RAD001 is mainly metabolized by CYP3A4 in the liver and to some extent in the intestinal wall. RAD001 is also a substrate of P-glycoprotein (PgP). Therefore, absorption and subsequent elimination of systematically absorbed RAD001 may be influenced by medicinal products that interact with CYP3A4 and/or P-glycoprotein. *In vitro* studies showed that RAD001 is a competitive inhibitor of CYP3A4 and of CYP2D6 substrates, potentially increasing the concentrations of medicinal products eliminated by these enzymes. In two phase III clinical trials in patients following kidney transplantation, strong inhibitors of CYP3A4 (azoles, antifungals, cyclosporine, erythromycin) have been shown to reduce the clearance of RAD001 therapy thereby increasing RAD001 blood levels. Conversely, Rifampin, a strong inducer of CYP3A4, increases the clearance of RAD001 thereby reducing RAD001 blood levels. Caution should be exercised when co-administering RAD001 with CYP3A4 inhibitors or inducers.

Pharmacokinetic drug to drug interactions with cancer agents have been evaluated in phase Ib studies. Based on currently available results, gemcitabine ([Study 2101] part 2) and paclitaxel ([Study 2104]) did not alter RAD001 pharmacokinetics to a clinically relevant extent whereas imatinib notably increased RAD001 exposure with a mean increase in AUC by a multiple of 3.7 for RAD001 administered weekly and two-fold for RAD001 administered daily [Study 2206]. Exposure to RAD001 in the presence of letrozole did not exceed that in monotherapy [Study 2108]. Co-administration of RAD001 did not influence pharmacokinetics of gemcitabine, imatinib or letrozole. Exposure to paclitaxel in the presence of RAD001 was slightly decreased (average by 23%). RAD001 pharmacokinetics in transplant patients was investigated in special populations such as subjects with hepatic or renal impairment, various ethnic groups and pediatric renal transplant patients. In subjects with mild-moderate hepatic impairment, mean AUC of RAD001 is increased by 2-fold while renal impairment does not affect the pharmacokinetics of RAD001. Age, weight (both over the adult range) and gender do not affect the pharmacokinetics of RAD001 to any clinically relevant extent. Also, pharmacokinetics does not alter in Asian patients whereas black patients have a 21% higher clearance compared to non-blacks. A single, escalating-dose study in Japanese subjects did not show a significant difference in dose-normalized systemic exposure.

The pharmacokinetic parameters derived for RAD001 given daily are summarized in Table 1-2.

Table 1-2 Steady-state RAD001 pharmacokinetics (daily dosing)

Parameter	5 mg	10 mg
N	4	6
t_{max} (h)	1 (1)	1 (1-6)
C_{min}^{ss} (ng/mL)	5.4 ± 1.8	13.2 ± 7.9
C_{max}^{ss} (ng/mL)	32 ± 9	61 ± 17
C_{max}^{ss}/Dose (ng/mL/mg)	6.4 ± 1.8	6.1 ± 1.7
$AUC\tau^{ss}$ (ng·h/mL)	238 ± 77	514 ± 231
$AUC\tau^{ss}$/Dose (ng·h/mL/mg)	48 ± 15	51 ± 23
C_{avg}^{ss} (ng/mL)	9.9 ± 3.2	21.4 ± 9.6

Values are median (range) for t_{max} and mean ± standard deviation for all others.
Dose-normalized parameters are per mg. τ is 24 h

Pharmacodynamic studies

Pharmacokinetic/pharmacodynamic modeling based on inhibition in a peripheral biomarker (S6 kinase inhibition in peripheral blood mononuclear cells) suggests that 5-10 mg daily should be an adequate dose to produce a high-degree of sustained target inhibition. Furthermore, molecular pharmacodynamic studies using immunohistochemistry in biopsied tumor tissue assessed the degree of inhibition and its duration (for p-S6, p-4E-BP1 and p-Akt expression) with the daily and weekly dosing. The pathologist was blinded for the biopsy sequence and found there was almost complete inhibition of p-S6 at all doses and schedules studied (p=0.001). Preliminary results suggest a dose-related decrease in p-4E-BP1 and increase in p-Akt expression with maximal effect at 10 mg daily and ≥ 50 mg weekly.

In [Study C2107], molecular changes were subsequently investigated through serial biopsying of tumors before and while on treatment (Tabernero et al 2005). Biopsying of tumors took place at week 4 of treatment (pharmacokinetics steady-state). All patients underwent a 24-hr post-dose biopsy. Patients following the weekly regimen had a further biopsy on Day 4-5 during the same week.

Molecular activity was measured by immunohistochemistry. In the absence of a reliable technique for measuring mTOR phosphorylation itself, the phosphorylation status of downstream markers S6 and eIF4G, for which reliable antibodies exist, was selected as reflecting the immediate pharmacodynamic effect of RAD001. Also measured were changes in the phosphorylation status of upstream AKT and the proliferation index Ki67. The daily regimen was associated with a high inhibition of p-S6 and p-eIF4G at 5 mg/d which was complete at 10 mg/d. In patients on the weekly regimen, p-S6 inhibition was complete and sustained at all dose levels while that of p-eIF4G was complete and sustained at 50 mg/d but not at 20 mg/wk. On both regimens numerous patients demonstrated apparent up-regulation of AKT which tended, however, not to persevere in patients at 50 mg/wk. The proliferation index was reduced in most patients, recovering in some of those on the 50 mg/wk regimen.

1.4.5 Safety in oncology studies

The largest completed phase III study of RAD001 to date was in adult patients with metastatic renal cell carcinoma. The data described below reflect exposure to RAD001 (n=274) and placebo (n=137) in a randomised phase III study. In total, 165 patients were exposed to RAD001 10 mg/day for ≥4 months. The median age of patients was 61 years (range 27 to 85).

The most common adverse reactions (incidence ≥10%) were stomatitis, rash, fatigue, asthenia, diarrhoea, anorexia, nausea, mucosal inflammation, vomiting, cough, peripheral oedema, infections, dry skin, epistaxis, pruritus, and dyspnoea. The most common grade 3-4 adverse reactions (incidence ≥2%) were infections, stomatitis, fatigue, and pneumonitis. The median duration of blinded study treatment was 141 days (range 19 to 451) for patients receiving RAD001 and 60 days (range 21 to 295) for those receiving placebo. The rates of treatment-emergent adverse reactions resulting in permanent discontinuation were 7% and 0% for the RAD001 and placebo treatment groups, respectively. Most treatment-emergent adverse reactions were grade 1 or 2 in severity. Grade 3 or 4 treatment-emergent adverse reactions were reported in 39% versus 7% of patients receiving RAD001 and placebo, respectively. Table 1-3 compares the incidence of treatment-emergent adverse reactions reported with an incidence of ≥5% for patients receiving RAD001 10 mg/day versus placebo. Adverse reactions in Table 1-3 are listed according to MedDRA system organ class. Within each system organ class, the adverse reactions are ranked by frequency, with the most frequent reactions first. Within each frequency grouping, adverse reactions are presented in order of decreasing seriousness. In addition, the corresponding frequency category using the following convention (CIOMS III) is also provided for each adverse reaction: very common (≥1/10); common (≥1/100 to <1/10); uncommon (≥1/1,000 to <1/100); rare (≥1/10,000 to <1/1,000); very rare (<1/10,000), including isolated reports.

Table 1-3 Adverse events suspected to be drug related in greater or equal to 10% of patients with adverse cancer reported in Phase I RAD001 monotherapy [Studies C2101, C2102, C2107]

	Frequency	Afinitor 10 mg/day N=274			Placebo N=137		
		All grades %	Grade 3 %	Grade 4 %	All grades %	Grade 3 %	Grade 4 %
**Any adverse reaction**		**89**	**35**	**3.3**	**58**	**6.6**	**0**
**Infections and infestations**							
Infections[a]	Very common	13	2.2	2.2	2.2	0	0
**Metabolism and nutrition disorders**							
Anorexia	Very common	19	<1	0	5.8	0	0
**Nervous system disorders**							
Dysgeusia	Very common	9.9	0	0	1.5	0	0
Headache	Common	8.8	0	0	5.1	0	0
**Respiratory, thoracic and mediastinal disorders**							
Cough	Very common	14	0	0	4.4	0	0
Pneumonitis [b]	Very common	12	3.3	0	0	0	0
Dyspnoea	Very common	10	1.8	0	2.9	0	0
Epistaxis	Very common	12	0	0	0	0	0
**Gastrointestinal disorders**							
Stomatitis [c]	Very common	42	3.3	0	8.0	0	0
Diarrhoea	Very common	21	1.5	0	3.6	0	0
Nausea	Very common	18	<1	0	8.0	0	0
Vomiting	Very common	15	<1	0	3.6	0	0
Dry mouth	Common	6.2	0	0	4.4	0	0
**Skin and subcutaneous tissue disorders**							
Rash	Very common	28	1.1	0	5.1	0	0
Dry skin	Very common	12	<1	0	4.4	0	0
Pruritus	Very common	12	<1	0	2.9	0	0
**General disorders and administration site conditions**							
Mucosal inflammation	Very common	17	1.1	0	1.5	0	0
Oedema peripheral	Very common	13	<1	0	3.6	0	0
Asthenia	Very common	22	1.8	0	9.5	<1	0
Fatigue	Very common	23	3.3	0	17	<1	0
Pyrexia	Common	5.5	0	0	2.2	0	0
**Investigations**							
Weight decreased	Common	5.5	0	0	<1	0	0
**Median Duration of Treatment (d)**			**141**			**60**	

CTCAE Version 3.0
[a] All infections reported including pneumonia, aspergillosis, candidiasis and sepsis.
[b] Includes alveolitis, interstitial lung disease, lung infiltration, pneumonitis, pulmonary alveolar haemorrhage, and pulmonary toxicity
[c] Stomatitis (including aphthous stomatitis) and mouth and tongue ulceration

Key treatment emergent laboratory abnormalities are presented in Table 1-4. It should be noted that the background frequency of laboratory abnormalities in the placebo arm was considerable in this advanced cancer population.

Table 1-4 Key laboratory abnormalities reported at a higher rate in the Afinitor arm than in the placebo arm

Table 2 Key laboratory abnormalities reported at a higher rate in the Afinitor arm than in the placebo arm

Laboratory parameter	Afinitor 10 mg/day N=274			Placebo N=137		
	All grades %	Grade 3 %	Grade 4 %	All grades %	Grade 3 %	Grade 4 %
**Haematology**[a)]						
Haemoglobin decreased	92	12	1.1	79	5.1	<1
Lymphocytes decreased	51	16	2.2	28	5.1	0
Platelets decreased	23	1.1	0	2.2	0	<1
Neutrophils decreased	14	0	<1	3.6	0	0
**Clinical chemistry**						
Cholesterol increased	77	4.4	0	35	0	0
Triglycerides increased	73	<1	0	34	0	0
Glucose increased	57	15	<1	25	1.5	0
Creatinine increased	50	1.5	0	34	0	0
Phosphate decreased	37	6.2	0	8.0	0	0
Aspartate transaminase (AST) increased	25	<1	<1	6.6	0	0
Alanine transaminase (ALT) increased	21	1.1	0	3.6	0	0
Bilirubin increased	2.9	<1	<1	2.2	0	0

CTCAE Version 3.0

[a] Includes reports of anaemia, leucopenia, lymphopenia, neutropenia, pancytopenia, thrombocytopenia

For further information about side effects and their management, see Section 6.1.1 and Section 6.1.2.

1.4.6 RAD001 Phase II study in patients with angiomyolipoma associated with TSC and/or LAM

Between July 13, 2005 and March 18, 2008, a total of 17 patients with angiomyolipoma associated with either TSC or LAM have been treated for up to 12 months with RAD001 in an investigator-initiated study (ClinicalTrials.gov Identifier NCT00457964) conducted at the Cincinnati Children's Hospital Medical Center (Principal Investigator: John J. Bissler, M.D., IND # 70,895). At this time, 12 month efficacy data is available for a total of 9 patients, of whom 4 received RAD001 5 mg daily and 5 received RAD001 10 mg daily. The percentage reductions in the sum of the volumes of target angiomyolipoma lesions (defined as lesions with a maximum diameter of at least 1 cm) were 67.5%, 64.4%, 39.4% and 18.4% for the 4 patients on RAD001 5-mg daily (mean reduction of 47.4%), and 61.7%, 60.2%, 60.1%, 27.4% and 12.8% for the 5 patients on RAD001 10 mg daily (mean reduction of 44.8%). While being numerically similar, the small number of patients involved prevents any firm conclusions from being made about the efficacy benefit of the 10 mg dose.

In the 5-mg cohort, the following adverse events possibly related to trial therapy were observed: rash (1 patient, grade 2), joint pain (1 patient, grade 2), pancreatitis (1 patient, grade 3, resolved after 27 days), bruises (1 patient, grade 1), bronchitis (1 patient, grade 2), sinusitis (2 patients, grade 2), cough (1 patient, grade 1), upper respiratory tract infection (1 patient, grade 2) and oral mucositis (2 patients, grade 1). The adverse events reported in the 10-mg

cohort were: oral mucositis (1 patient, grade 3), ear pain (1 patient, grade 1), pneumonia (1 patient, grade 2, resolved after 4 days), pneumonitis (1 patient, grade 3, resolved after 17 days), cholecystitis (1 patient, grade 3, resolved after 5 days), upper respiratory tract infection (1 patient, grade 2), nose bleed (1 patient grade 2), sinusitis (1 patient, grade 2), oral mucositis (1 patient, grade 1; and 2 patients, grade 2). This data is consistent with the known safety profile of RAD001 (daily dose of 10 mg) in cancer patients, and no new safety issues have been identified. (Data kindly provided by John Bissler, MD, on March 18, 2008.)

1.4.7 Sirolimus Phase II study in patients with angiomyolipoma associated with TSC and/or LAM

Another study evaluated the efficacy of rapamycin in spontaneous and TSC-associated pulmonary angiomyolipomas (Bissler et al 2008). Sirolimus was administered for 12 months and patients were followed for an additional 12 months. The primary endpoint was the reduction in angiomyolipoma volume at 1 year, and secondary endpoints included angiomyolipoma volume at 2 years, and spirometric measurements, lung volumes, diffusing capacity, results of the 6-minute walk test, and the percentage of the cyst volume at 1 and 2 years. Serial MRI of angiomyolipomas and brain lesions, CT scans of lung cysts, and PFTs were performed.

The initial sirolimus dose was 0.25 mg/m² of body-surface area. Sirolimus levels were measured at 2 weeks, and the dosage was adjusted to achieve a blood sirolimus level between 1 and 5 ng/mL. If the target angiomyolipoma lesions had not decreased by 10% of the baseline value in the longest coronal-plane dimension at the 2-month visit, the dose was increased to achieve a blood sirolimus level of 5 to 10 ng/mL. At the 4-month visit, if the threshold of a 10% reduction from the baseline value had not been reached the dose was increased to achieve a blood sirolimus level of 10 to 15 ng/mL. The dose chosen at the 4-month visit was continued through 12 months. Although the actual administered doses were not reported, 19 of the 20 patients had the dose of sirolimus increased to achieve blood levels of 10 to 15 ng/mL on the basis of imaging results at 2 months and 4 months.

The mean (±SD) angiomyolipoma volume at 12 months was 53.2±26.6% of the baseline value (P<0.001) and at 24 months was 85.9±28.5% of the baseline value (P = 0.005). At 24 months, five patients had a persistent reduction in the angiomyolipoma volume of 30% or more. During the period of sirolimus therapy, among patients with LAM, the mean FEV_1 increased by 118±330 ml (P = 0.06), FVC increased by 390±570 ml (P<0.001), and the residual volume decreased by 439±493 ml (P = 0.02), as compared with baseline values. One year after sirolimus was discontinued, the FEV_1 was 62±411 ml above the baseline value, the FVC was 346±712 ml above the baseline value, and the residual volume was 333±570 ml below the baseline value; cerebral lesions were unchanged. Five patients had six serious adverse events while receiving sirolimus, including diarrhea, pyelonephritis, stomatitis, and respiratory infections.

The authors noted that although angiomyolipoma regressed during sirolimus therapy, they tended to increase in volume after the therapy was stopped. Some patients with LAM had improvement in spirometric measurements and gas trapping that persisted after treatment. Lung function in patients who did not have LAM was not reported. The authors concluded

that suppression of mTOR signaling might constitute an ameliorative treatment in patients with the TSC or sporadic LAM.

1.5 History of Amendments

2 Study rationale/purpose

This study will evaluate the anti-tumor activity of RAD001 (10 mg/day) *versus* matching placebo in patients with angiomyolipomata associated with TSC or sporadic LAM.

Patients eligible for the current trial should have at least one angiomyolipoma of 3 cm or more in the largest dimension.

We believe that volumetric assessment of target lesions using MRI will allow a more accurate assessment of lesion response to treatment and will provide a better approximation of actual tumor volume. The techniques proposed will also allow comparisons between three-dimensional assessments and the unidimensional ones.

Early investigators recognized a striking similarity between the pulmonary lesions seen in otherwise healthy women with LAM, and those seen in patients with TSC and lung involvement (Cornog 1966, Capron 1983). This led to the hypothesis that TSC-LAM and sporadic LAM might share common pathogenetic mechanisms. This observation and the discovery of *TSC1* and *TSC2* in the 1990s and extensive genetic research in the last few years helped establish that both TSC and sporadic LAM are linked to mutations in the *TSC1* and *TSC2* genes (Costello 2000, Strizhefa 2001).

It is now established that germline mutations in *TSC1* and *TSC2* are not present in patients with sporadic LAM; in contrast, TSC-LAM is characterized by germline mutations in *TSC2* (Astrinidis 2000, Sato 2002). However, LAM cells in both TSC-LAM and sporadic LAM carry mutations. Patients with sporadic LAM have two acquired mutations (typically in *TSC2*) whereas patients with TSC-LAM have one germline and one acquired mutation, typically in *TSC2* (Juvet et al 2007).

In summary, the hamartin-tuberin signaling via mTOR is a valid and appropriate therapeutic target both in patients with TSC-LAM as well as in patients with sporadic LAM (Johnson 2006).

3 Objectives

3.1 Primary objective

To compare the angiomyolipoma response rate on RAD001 versus placebo in patients with angiomyolipomata associated with either tuberous sclerosis complex or sporadic lymphangioleiomyomatosis.

3.2 Secondary objectives

To compare RAD001 versus placebo with respect to:

1. Time to angiomyolipoma progression.

2. Skin lesion response rate.
3. Change from baseline in plasma angiogenic molecules, e.g., VEGF, basic FGF, PLGF, soluble VEGF receptor1, and soluble VEGF receptor2.
4. Renal function assessed using calculated creatinine clearance
5. Safety as assessed by the National Cancer Institute's (NCI) Common Terminology Criteria for Adverse Events (CTCAE), version 3.0.

In RAD001 treatment arm to:

1. Characterize the pharmacokinetics of RAD001 in this patient population, specifically in terms of exposure.
2. Describe the time to angiomyolipoma response, the duration of angiomyolipoma response, and the duration of skin lesion response.

3.3 Exploratory objectives

4 Study design

This is a double-blind, randomized, parallel group, placebo-controlled, multi-center phase III study of treatment with once a day oral dose of RAD001 10 mg versus matching placebo in patients with angiomyolipoma associated with either TSC or sporadic LAM.

There are six separate phases in this study: pre-treatment (screening/baseline), blinded treatment, open-label, extension, follow-up, and non-interventional follow-up. Each of these phases is described in detail below.

4.1 Pre-treatment phase (screening/baseline)

At screening/baseline, the investigator or his/her designee will assign a unique number (refer to Section 6.3) to patients being considered for the study. Patients are required to sign an Informed Consent Form prior to any study screening evaluations. Once the patient provides a **signed informed consent form and eligibility is confirmed (i.e., all inclusion/exclusion criteria have been verified)**, the investigator or his/her designee should register the patient using an Interactive Web Response System (IWRS), a central patient screening/randomization system. Refer to Section 6.3 and Section 6.4 for complete details.

During the 21-day screening period, all patients will have a CT/MRI of the kidneys performed for identification of angiomyolipomata and SEGA lesions; the kidney CT/MRI will be used to assess patient eligibility. Although the study will use an Independent Central Radiology Review, the decision to randomize the patient will be made based on the judgment of the investigator and local radiologist, as the central review requires a turn-around time of up to 3 weeks. Once the patient is randomized, the baseline kidney CT/MRI should be sent as soon as possible by the study site to the Independent Central Radiology Reviewer. There is no plan to collect radiological measurements made by the local radiologist, partly because not all sites will have the same methods of measurement, but also because all data analysis will be based on the measurements obtained from the central radiological review.

Hepatitis screening

Prior to randomization, the following three categories of patients should be tested for hepatitis B viral load and serologic markers, that is, HBV-DNA, HBsAg, HBs Ab, and HBc Ab:

1. All patients who currently live in (or have lived in) Asia, Africa, Central and South America, Eastern Europe, Spain, Portugal, and Greece. [http://wwwnc.cdc.gov/travel/yellowbook/2010/chapter-2/hepatitis-b.aspx#849]
2. Patients with any of the following risk factors:
    - known or suspected past hepatitis B infection,
    - blood transfusion(s) prior to 1990,
    - current or prior IV drug users,
    - current or prior dialysis,
    - household contact with hepatitis B infected patient(s),

- current or prior high-risk sexual activity,
- body piercing or tattoos,
- mother known to have hepatitis B
- history suggestive of hepatitis B infection, e.g., dark urine, jaundice, right upper quadrant pain.

3. Additional patients at the discretion of the investigator

If a patient tests positive, he/she will be considered ineligible for the study according to Exclusion Criterion 8. Please note that patients who test negative for HBV-DNA, HBsAg, and HBc Ab but positive for HBs Ab, due to prior history of vaccination against Hepatitis B will be eligible. The fact that the patient had been vaccinated should be entered into the patient's Medical History CRF.

Screening for hepatitis C

Patients with any of the following risk factors for hepatitis C should be tested using quantitative RNA-PCR:

- known or suspected past hepatitis C infection (including patients with past interferon 'curative' treatment),
- blood transfusions prior to 1990,
- current or prior IV drug users,
- current or prior dialysis,
- household contact of hepatitis C infected patient(s),
- current or prior high-risk sexual activity,
- body piercing or tattoos

At the discretion of the investigator, additional patients may also be tested for hepatitis C.

If a patient tests positive, they will be considered ineligible for the study according to Exclusion Criterion 8.

For patients who have already been randomized and received study drug prior to the approval of Amendment 1, the same screening process should be followed at the patient's next visit.

If the patient tests positive for Hepatitis B, the investigator should follow the guidelines according to Table 4-1 and Table 4-2.

Please refer to Table 7-1 and Table 7-2 for HCV RNA-PCR monitoring schedule for those patients with positive HCV RNA-PCR baseline tests who do not meet the reactivation criteria outlined in Table 4-3. If the patient tests positive for hepatitis C, and the criteria for reactivation according to Table 4-3 are observed, trial therapy should be discontinued and further treatment is up to the investigators discretion.

Table 4-1 Action to be taken for positive baseline hepatitis B results for patients that are active prior to Amendment 1 approval

Test	Result	Result	Result	Result	Result
HBV-DNA	+	+ or -	-	-	-
HBsAg	+ or -	+	-	-	-

Test	Result	Result	Result	Result	Result
HBs Ab	+ or -	+ or -	+<br>and no prior HBV vaccination	+ or -	-<br>or + with prior HBV vaccination
HBc Ab	+ or -	+ or -	+ or -	+	-
Recommendation	Prophylaxis treatment should be started and study drug dose interruption is recommended for 14 days<br>Monitor HBV-DNA approximately every 4-8 weeks		No prophylaxis<br>Monitor HBV-DNA approximately every 3-4 weeks		No specific action

Table 4-2 Guidelines for management of hepatitis B for patients that are active prior to Amendment 1 approval

**HBV reactivation (with or without clinical signs and symptoms)***	
For patients with baseline results:<br>Positive HBV-DNA<br>OR<br>positive HBsAg<br>-----------------------------------<br>reactivation is defined as:<br>[Increase of 1 log in HBV-DNA relative to baseline HBV-DNA value **OR** new appearance of measurable HBV-DNA]<br>AND<br>ALT elevation x 5 ULN	**Treat**: Start a second antiviral<br>AND<br>Interrupt study drug administration until resolution:<br>≤ grade 1 ALT (or baseline ALT, if > grade 1) and<br>≤ baseline HBV-DNA levels<br>**If resolution occurs within ≤ 28 days** study drug should be re-started at one dose lower, if available. (see Table 6-4 - Dose levels for dose adjustments) If the patient is already receiving the lowest dose of study drug according to the protocol, the patient should restart at the same dose after resolution. Both antiviral therapies should continue at least 4 weeks after last dose of study drug.<br>**If resolution occurs > 28 days** Patients should discontinue study drug but continue both antiviral therapies at least 4 weeks after last dose of study drug.
For patients with baseline results:<br>Negative HBV-DNA **and** HBsAg<br>AND<br>[Positive HBs Ab (with no prior history of vaccination against HBV), **OR** positive HBc Ab]<br>-----------------------------------<br>reactivation is defined as:<br>New appearance of measurable HBV-DNA	**Treat** : Start first antiviral medication<br>AND<br>**Interrupt** study drug administration until resolution:<br>≤ baseline HBV-DNA levels<br>**If resolution occurs within ≤ 28 days** study drug should be re-started at one dose lower, if available. (see Table 6-4 - Dose levels for dose adjustments) If the patient is already receiving the lowest dose of study drug according to the protocol, the patient should restart at the same dose after resolution. Antiviral therapy should continue at least 4 weeks after last dose of study drug.<br>**If resolution occurs > 28 days** Patients should discontinue study drug but continue antiviral therapy at least 4 weeks after last dose of study drug.
* All reactivations of hepatitis B are to be recorded as grade 3 (CTCAE v 3.0 Metabolic Laboratory/Other: Viral Re-activation), unless considered life threatening by the investigator; in which case they should be recorded as grade 4 (CTCAE v 3.0 Metabolic Laboratory/Other: Viral Re-activation). Date of viral reactivation is the date on which **both** DNA and ALT criteria were met (e.g. for a patient who was HBV-DNA positive on 01-JAN-10 and whose ALT reached ≥ 5 × ULN on 01-APR-10, the date of viral reactivation is 01-APR-10).	

Table 4-3 Guidelines for management of hepatitis C for patients that are active prior to Amendment 1 approval

**HCV reactivation***	
For patients with baseline results:<br>Detectable HCV-RNA<br><br>reactivation is defined as:<br>ALT elevation x 5 ULN	Discontinue study drug

For patients with baseline results: Knowledge of past hepatitis C infection with no detectable HCV-RNA<br><br>reactivation is defined as: New appearance of detectable HCV-RNA	Discontinue study drug
* All reactivations of hepatitis C are to be recorded as grade 3 (CTCAE v 3.0 Metabolic Laboratory/Other: Viral Re-activation), unless considered life threatening by the investigator; in which case they should be recorded as grade 4 (CTCAE v 3.0 Metabolic Laboratory/Other: Viral Re-activation).	

Other screening tests include blood sampling, urinalysis, WHO performance status, ECG, and biomarker evaluations. If safety laboratory collections are collected more than 14 days prior to Treatment Day 1 (i.e., day of first dose of study drug), they will need to be repeated prior to the patient's first dose of study drug. The laboratory collections that must be repeated are: hematology, biochemistry and lipid profile, coagulation, urinalysis and pregnancy testing.

Patients with skin lesions at baseline will have digital photos of these lesions taken during screening. Screening evaluations will also include administration of informed consent, demography, inclusion/exclusion criteria, relevant medical history/current medical conditions, a physical examination (including a neurological examination), vital signs and other additional study entry evaluations. All screening/baseline evaluations should be completed in the 21 days prior to Treatment Day 1. A complete list of screening evaluations is provided in the schedule of evaluations (Table 7-1). All of the above assessments/procedures must be conducted prior to randomization.

4.2 Blinded treatment phase

Patients who meet the study eligibility criteria will be randomized to receive RAD001 or matching placebo. The randomization ratio is 2:1, with two patients being randomly assigned to RAD001 for every one patient randomly assigned to matching placebo. Randomization will be stratified by one factor with three categories: (1) TSC as underlying disease and patient using enzyme-inducing anti-epileptic drugs (EIAED) at randomization, (2) TSC as underlying disease and patient not using EIAED at randomization, and (3) sporadic LAM as underlying disease. Note that patients with sporadic LAM are not expected to be using EIAED at randomization. The following drugs qualify as EIAED: phenytoin (Dylantin®, Dilantin Kapseals®, Dilantin Infatabs®, Eptoin®, Epanutin®, Diphenin®, Dipheninum®, Phenytek®), mephenytoin (Mesantoin®), carbamazepine (Tegretol®, Biston®, Calepsin®, Carbatrol®, Epitol®, Equetro®, Finlepsin®, Sirtal®, Stazepine®, Telesmin®, Teril®, Timonil®, Trimonil®, Epimaz®, and Degranol®), phenobarbital (Luminal®), pentobarbital (Nembutal®), primidone (Mysoline®), and oxcarbazepine (Trileptal®). Randomization and study medication management will be done through IWRS.

This study does not have a fixed treatment duration. Patients will have their first daily dose of RAD001 or matching placebo at Visit 2 (Treatment Day 1) and will continue on treatment until angiomyolipoma progression (as defined in Section 7.5.2), an unacceptable toxicity, withdrawal of consent or investigator decision to discontinue the patient from study treatment.

Each patient will start with a dose of 10-mg/day. Dose adjustments (reduction, interruption or possible dose re-escalation to starting dose) will occur based on safety findings. All doses

taken by the patient and all dose changes during the study must be recorded on the Dosage Administration Record CRF.

Safety evaluations are routinely performed at each visit according to the visit schedule (Table 7-1). Patient must be in a fasting state (at least 12 hours) at the time of blood sampling for all laboratory evaluations including the lipid profile. Hematology and biochemistry evaluations will be performed according to the visit schedule (Table 7-1). Note that hematology and biochemistry assessments are performed at screening and do not need to be repeated on Treatment Day 1 if Treatment Day 1 occurs within 14 days of the screening visit. Otherwise, the patient will need to repeat the screening visit blood collections. All blood samples obtained at each visit will be sent to a Central Laboratory for analysis. If laboratory results are requested on an urgent basis, the attending physician will use the local laboratory results for treatment decisions. Complete details regarding all study required safety assessments are provided in Section 7.6.

During this phase of the study, all patients will have a CT/MRI of the kidney performed at 12, 24 and 48 weeks after the start of study treatment, and annually thereafter until angiomyolipoma progression. A CT/MRI of the kidneys should be repeated at the End of Treatment (EOT) visit if the patient has discontinued for any reason other than angiomyolipoma progression, and if it has been more than 8 weeks since their most recent kidney CT/MRI during the first year of treatment, or more than 6 months since their most recent kidney CT/MRI thereafter.

All kidney CT/MRIs obtained during the blinded treatment phase will be sent to the central radiologist within 2 days of the scan for an independent centralized radiology review. The central radiologist will determine the angiomyolipoma volume and verify whether the radiological criteria for angiomyolipoma response have been met (see Section 7.5.2). Since not all study centers will have the technical capability to measure angiomyolipoma volume, there is no plan to collect data from the local radiologist at each site. MRI and CT scans must be digitized and sent to Central Radiology within 2 days of the scan date. The central review of the CT/MRIs will be available no more than 3 weeks after its receipt by the central reviewer, and once available the results will be sent immediately to the study site. **If the patient is assessed as having a response, a repeat radiological confirmation should be performed approximately 12 weeks from the initial observation,(and no sooner than 8 weeks).** Clinical suspicion of angiomyolipoma progression at any time requires a physical examination and radiological confirmation to be performed promptly rather than waiting for the next scheduled radiological assessment. If angiomyolipoma progression was **unequivocal** according to the local radiologist, then the investigator should contact the Novartis Clinical Trial Head (CTH) or designee for review and for unblinding prior to receiving confirmation from central radiology review.

Other tests that will be conducted routinely during the blinded treatment phase include laboratory tests for safety, physical examinations (including a neurological assessment), vital signs, WHO performance status, ECG, PK. In addition, for LAM patients, PFTs will be routinely carried out at 6, 12, 18, 24 weeks and every 12 weeks thereafter. In addition to these tests, patients with skin lesions at baseline will have digital photos of their skin lesions taken every 12 weeks. A confirmation of skin lesion response should be performed approximately 12 weeks (and no sooner than 8 weeks) after the first assessment of response. A complete list

of evaluations can be found in Table 7-1. If unforeseen circumstances (i.e., unexpected personal reasons) prevent the patient from complying with the established visit schedule, the site can re-schedule the visit within the prescribed visit window as noted in Table 7-1. The reason(s) for any visit or treatment delays will be documented in the Comment section of the CRF for the appropriate visit.

Pharmacokinetic assessments will be performed according to the plan provided in Section 7.7. Trough pharmacokinetic assessments (pre-dose, C_{min}) are planned for all patients at weeks 2, 4, 12, 24 and 48. An additional pharmacokinetic assessment will be done at each of these visits 2.0 hours (± 30 mins, C_{2h}) after study drug administration.

Biomarker research studies will be performed according to the blood collection plan provided in Section 7.8. During the blinded treatment phase of the study, 3 mL of blood will be collected at screening/baseline, at 4, 12, 24, 36 and 48 weeks and at end of treatment. On-treatment samples will be compared to baseline samples for RAD001 effects on plasma angiogenic molecules, e.g., VEGF, basic FGF, PLGF, soluble VEGF receptor1, and soluble VEGF receptor2.

Patients will donate 3 mL of whole blood at screening/baseline for genetic mutational analyses of *TSC1* and *TSC2* genes. No tumor material will be investigated as part of this protocol.

All patients taking antiepileptic medications and who completed the questionnaire at baseline, will complete the SSQ at 24 weeks, and every 24 weeks thereafter per the visit schedule (Table 7-1). Patients who are ongoing at the time Amendment 1 is approved should have the endocrine blood test at their next scheduled visit.

4.3 Open label treatment phase

If angiomyolipoma progression (as defined in Section 7.5.2) is documented by central radiology review or an angiomyolipoma-related bleeding of grade 2 or worse (NCI CTCAE, version 3.0) during the blinded treatment phase, then the treating physician may proceed to unblind the patient. Central radiology review of the CT/MRIs will be completed no later than 3 weeks after receipt by the central reviewer, so the unblinding may not be able to take place until up to 3 weeks after the progression was actually observed. However, if progression was unequivocal according to the local radiologist, then the investigator should contact the Novartis Clinical Trial Head (CTH) or designee to proceed with unblinding without waiting for confirmation from central radiology review.

Following unblinding, patients who had been receiving placebo may be offered open-label treatment with RAD001 if the treating physician believes the patient could benefit from this therapy.

For the open-label phase of the study, the most recent kidney CT/MRI from the blinded treatment phase of the study will be used as the baseline, and further kidney CT/MRIs will be conducted at 12, 24 and 48 weeks after the start of open-label RAD001, and annually thereafter, until angiomyolipoma progression, unacceptable toxicity or discontinuation for any other reason. An additional CT/MRI of the kidney will be obtained at End of Treatment in the open-label RAD001 phase, provided discontinuation was not for angiomyolipoma progression and if it had been more than 8 weeks since the most recent CT/MRI of the kidney during the

first year of open-label treatment or more than 6 months since the most recent CT/MRI thereafter. For each patient, the same imaging modality should be used throughout the trial. All kidney imaging scans obtained in the open-label phase of the trial will be sent in for central radiological review within 2 days of the scan.

Patients receiving open-label treatment with RAD001 will continue having safety and efficacy assessments as in the blinded treatment phase, as described in the Visit Evaluation Schedule provided in Table 7-2, with the exception of biomarker and pharmacokinetic assessments which will not be performed in the open-label phase.

Open-label treatment with RAD001 will continue until the patient again presents with angiomyolipoma progression (second occurrence) which is either radiologically documented or is an angiomyolipoma-related bleeding of grade 2 or worse (NCI CTCAE, version 3.0). At this point, the patient will be discontinued from the study and will enter the follow-up phase.

The investigator or his/her designee will **not** disclose patient unblinding information to the central radiology reviewers. Due to the unblinding of a subset of patients uponangiomyolipoma progression, members of the Novartis clinical trial team may become unblinded to an individual patient's treatment during the conduct of the trial including team members involved in source data verification. The blinding of the central radiology review, the basis for the primary analysis of the primary endpoint of the trial, will be maintained despite the planned unblinding.

4.4 Follow-up phase

All patients will have a follow-up visit scheduled 4 weeks (28 days) after the last dose of study treatment to capture AEs and SAEs that may have occurred after discontinuation from the study treatment. Beyond these 28 days, any serious adverse events that are suspected to be related to the study drug and occur within the next 8 weeks (56 days) will also be collected. Medications/therapies given to the patient in treatment of SAEs during the 8 weeks after study treatment discontinuation must be recorded on the Concomitant Medications/Significant Non-Drug Therapies CRF, unless the patient begins a non-study anti-angiomyolipoma therapy, at which time collection of SAEs, AEs and concomitant medications will cease. Patients without angiomyolipoma progression at the time of discontinuation of study treatment will be followed with CT/MRI tumor assessments annually until eventual angiomyolipoma progression, the start of any non-study systemic anti-angiomyolipoma therapy, withdrawal of consent, or until end of the study, whichever occurs first. During this follow up period, the site will continue to send CT/MRIs for central review, and use of non-study systemic anti-angiomyolipoma therapies will be recorded.

4.5 Extension phase

There are two distinct parts of this trial, the core and extension phase. The core phase is from the start of the trial up to the time when the results of the final primary analysis are known. This primary analysis will be performed using all data up to the data cutoff date, which is defined as 6 months after the last patient is randomized. Following a database freeze, the statistical analysis will be performed. During this time, patients will continue on the blinded treatment phase or open-label phase as before, until the time when the results of the final data analysis are known and the core phase of the trial is terminated.

If this final data analysis fails to show superiority of RAD001 over placebo, all patients will be discontinued from the study and will have end of treatment evaluations as indicated in Table 7-1 and Table 7-2. In this case there will be no extension phase.

If the final trial results favor RAD001, then an extension phase will be launched. All patients still receiving study treatment at this time, as well as those being followed for post-treatment evaluation, will be given the option of starting open-label RAD001. Similarly, patients receiving open-label RAD001 at the end of the core phase will be offered to continue into the extension period. The extension phase will run until 4 years after the last patient was randomized, ensuring patient follow-up of 4-5 years (assuming patient accrual over a period of approximately 12 months).

Those patients entering the extension phase, who had previously only been receiving placebo, will have scheduled assessments beginning at Treatment Day 1 according to Table 7-2. Patients that had been on active RAD001 prior to beginning the extension phase will simply continue their sequence of assessments, according to Table 7-1 and Table 7-2. Accordingly, patients that had been on active RAD001 in the Open-label phase prior to beginning the extension phase will simply continue their sequence of assessments according to Table 7-2. Patients in the extension phase will continue to have the same safety and efficacy assessments as in the core phase until angiomyolipoma progression, with the exception of biomarker assessments and pharmacokinetic assessments which will not be performed in the extension phase.

RAD001 will be provided free of charge by Novartis during the extension phase and until such time when the patient has to stop treatment with RAD001 because of adverse event(s), abnormal laboratory value(s), angiomyolipoma progression, the patient’s condition no longer requires RAD001 therapy, withdrawal of consent, lost to follow-up, death, Novartis discontinues development of RAD001 for this indication, or end of the extension phase, whichever comes first. For all patients receiving RAD001 at the end of the extension phase, RAD001 will continue to be provided free of charge for as long as the medication is not commercialized and added to the list of reimbursed medications for patients with TSC.

Figure 4-1 Study flowchart

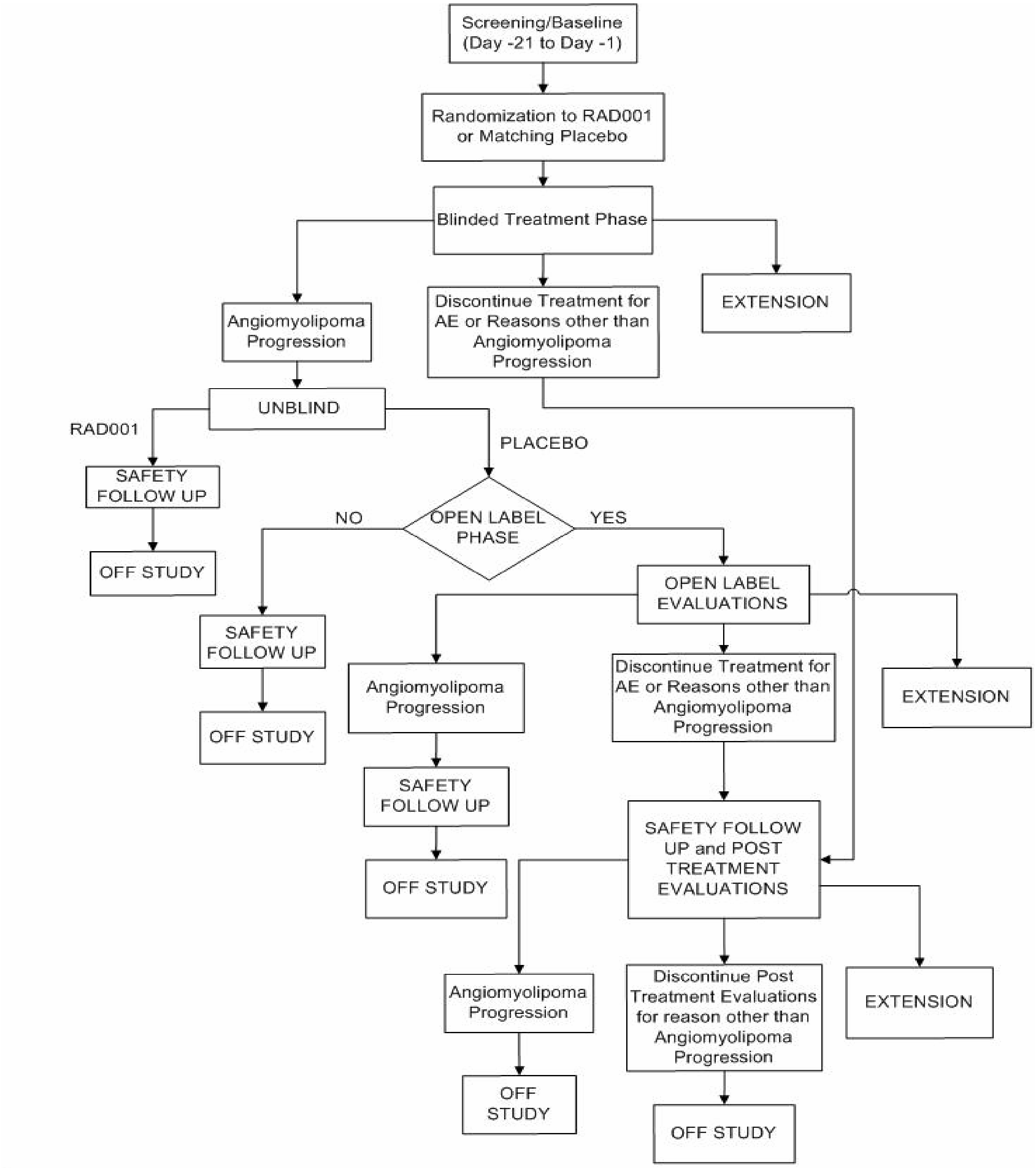

__Safety Follow Up:__ *Collection of AEs and SAEs that occur within 28 days following treatment discontinuation. SAEs suspected to be related to study drug will be collected for an additional 8 weeks (56 days).*
__Post Treatment Evaluations:__ *CT or MRI of the kidneys (and MRIs of the brain, if applicable) annually.*
__Open Label Evaluations:__ *CT or MRI of the kidneys (and MRIs of the brain, if applicable) to be done 12, 24 and 48 weeks after the start of open label RAD001 and annually thereafter. Safety and efficacy assessments to be carried out as in the blinded treatment phase (with the exception of biomarker and pharmacokinetic assessments, which will not be done in the open label phase).*

4.6 Non-interventional follow-up phase

For patients who permanently discontinue everolimus (off-study) at the completion of the extension phase for reasons other than angiomyolipoma progression, a single CT/MRI scan of the kidney will be collected one-year after the last study treatment date. If angiomyolipoma bleeding (≤ grade 2) occurs, or if intervention is needed at any time during this one-year non-non-interventional period, a CT/MRI will be completed prior to starting any intervention and the patient will discontinue this non-interventional follow-up phase. Interventions include treatment with systemic mTOR inhibitors such as everolimus or rapamycin, embolization, or (partial) nephrectomy. These additional CT/MRIs will be sent for central review. Any use of non-study systemic anti-angiomyolipoma therapies will be recorded during this supplementary one-year period.

Patients who do not have angiomyolipoma progression at the time treatment discontinuation from the extension phase and are being followed with CT/MRI tumor assessments will exit the study, unless the patient qualifies and elects to participate in the non-interventional follow-up phase. Patients who previously discontinued treatment with everolimus during the extension phase and have not yet taken their first annual CT/MRI may also be included in this non-interventional follow-up phase, if otherwise eligible.

Patients must report to the site any angiomyolipoma-related bleeding events. Diagnostic procedure(s) pertaining to angiomyolipoma-related bleeding should also be reported. All other adverse events that may occur within the one-year period will be collected, as reported by the patient for the DS&E database.

Concomitant medications and non-drug therapies given in treatment of angiomyolipoma or angiomyolipoma-related disease progression must be reported. All other concomitant medications and non-drug therapies will only be collected, as reported by the patient for the DS&E database.

Figure 4-2 Non-intervention follow-up phase schema

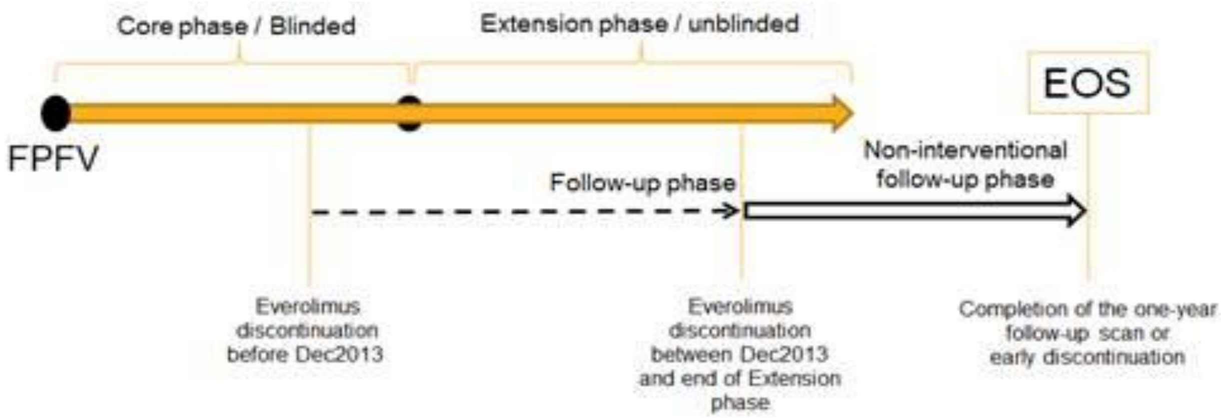

4.7 End of Study

The End of Study will take place after all patients participating in the non-interventional follow-up phase have either completed the one-year of non-interventional follow-up or have exited the study.

4.7.1 Patient participation

Any individual patient who has already completed at least 4 years of treatment with study medication may exit the study at any time. If a patient has already completed 4-years of study treatment, and does not intend to continue treatment with commercially-available everolimus, the patient may then begin participation in the non-interventional follow-up phase (Section 4.6). Patients who discontinue early from the extension phase and will not be treated for their angiomyolipomas may also be included in the non-interventional follow-up phase as long as their regular annual CT/MRI scan is during the non-interventional phase.

5 Population

The target population is comprised of patients aged 18 years or older with angiomyolipomata associated with either TSC or sporadic LAM. It is anticipated that approximately 125 patients will need to be screened to enroll at least 99 patients. Subjects will be recruited from approximately 25 sites worldwide.

Inclusion/exclusion criteria

The investigator or his/her designee must ensure that all patients who meet the inclusion and exclusion criteria during screening are offered enrollment in the study.

No additional exclusions can be applied by the investigator, in order that the study population will be representative of all eligible patients.

Patients must have screening evaluations performed to ensure potential patients being considered by the investigator meet all inclusion and exclusion criteria. The investigator or his/her designee must review the results of all screening evaluations, to ensure that all inclusion and exclusion criteria have been satisfied prior to randomization of that patient into the study. Only laboratory results from the Central Laboratory will be used to determine patient eligibility for the study.

All study patients must be thoroughly informed about all aspects of the study, including the study visit schedule and required evaluations and all regulatory requirements for informed consent. The written informed consent must be obtained prior to the performance of any screening evaluations. If the patient is unable to read, an impartial witness and/or the patient’s parent or legal guardian must be present during the entire informed consent discussion. The following criteria apply to all patients enrolled into the study.

5.1 Inclusion criteria

1. Male or female ≥ 18 years of age.
2. Clinically definite diagnosis of tuberous sclerosis according to the modified Gomez criteria (Roach 1998, Hyman 2000, Table 5-1) **or** sporadic LAM (biopsy-proven or compatible chest CT scan). Clinically definite diagnosis of tuberous sclerosis according to the modified Gomez criteria is defined as either of the following:
   - Two Major Features from Table 5-1.
   - One Major Feature plus two Minor Features from Table 5-1.
3. Clinically definite diagnosis of renal angiomyolipoma.
4. Presence of at least one angiomyolipoma ≥ 3 cm in its longest diameter using CT/MRI.
5. If female and of child-bearing potential, documentation of negative pregnancy test prior to enrollment. Sexually active pre-menopausal female patients (and female partners of male patients) must use highly effective contraceptive measures, while on study and for 8 weeks after ending treatment.
6. Written informed consent according to local guidelines.

Table 5-1 Diagnostic criteria for Tuberous Sclerosis Complex

**Major Features**
1. Facial angiofibromas or forehead plaque<br>2. Non-traumatic ungual or periungual fibroma<br>3. Hypomelanotic macules (three or more)<br>4. Shagreen patch (connective tissue nevus)<br>5. Multiple retinal nodular hamartomas<br>6. Cortical tuber[a]<br>7. Subependymal nodule<br>8. Subependymal giant cell astrocytoma<br>9. Cardiac rhabdomyoma, single or multiple<br>10. Lymphangioleiomyomatosis[b]<br>11. Renal angiomyolipoma[b]
**Minor Features**
1. Multiple, randomly distributed pits in dental enamel<br>2. Hamartomatous rectal polyps[c]<br>3. Bone cysts[d]<br>4. Cerebral white matter radial migration lines[a,d]<br>5. Gingival fibromas<br>6. Non-renal hamartoma[c]<br>7. Retinal achromic patch<br>8. 'Confetti' skin lesions<br>9. Multiple renal cysts[c]
Definite Tuberous Sclerosis Complex:<br>Either two Major Features or one Major Feature plus two Minor Features.
[a] The co-occurrence of cerebral cortical dysplasia and cerebral white matter radial migration lines should be considered as one major feature of TSC.<br>[b] In patients with both lymphangioleiomyomatosis and renal angiomyolipoma, another feature of TSC must be identified before a definite diagnosis is assigned.<br>[c] Histologic confirmation of these features is suggested.<br>[d] Radiographic confirmation of these features is sufficient.

5.1.1 Non-interventional follow-up phase/Inclusion criteria

7. Patients who do not have angiomyolipoma progression at the time of study treatment discontinuation and do not plan to continue treatment of their angiomyolipoma(s) with systemic therapy
8. Completion of the non-interventional follow-up phase informed consent

5.2 Exclusion criteria

1. Patients with angiomyolipoma which, in the opinion of the investigator, requires surgery at the time of randomization.
2. Angiomyolipoma-related bleeding or embolization during the 6 months prior to randomization.
3. History of myocardial infarction, angina or stroke related to atherosclerosis.
4. Impaired lung function defined as following:

For patients without lymphangioleiomyomatosis (LAM)

- Known impaired lung function (e.g. FEV_1 or $DL_{co} \leq 70\%$ of predicted)

Note: pulmonary function testing at baseline is not required for patients without LAM

For patients with LAM

- $DL_{co} \leq 35\%$, or
- O_2 saturations below normal at rest, or
- O_2 saturation ≤ 88% on 6 minute walking test with up to 6 liters O_2/minute nasal oxygen

5. Chylous ascites sufficient to affect diaphragmatic function or pulmonary function testing.
6. Significant hematological or hepatic abnormality (i.e., transaminase levels > 2.5 × upper limit of normal (ULN), serum bilirubin > 1.5 × ULN, hemoglobin < 9g/dL, platelets < 80,000/mm^3, or absolute neutrophil count < 1,000/mm^3).
7. Pregnancy or breast feeding.
8. Intercurrent infection at date of randomization.
9. Prior history of organ transplantation.
10. Recent surgery (involving entry into a body cavity or requiring sutures) within the 2 months prior to randomization.
11. Prior therapy with mTOR inhibitors (e.g., sirolimus, temsirolimus, everolimus).
12. Use of an investigational drug within the 30 days prior to randomization.
13. Uncontrolled hyperlipidemia: Fasting serum cholesterol > 300 mg/dL (or > 7.75 mmol/L), AND fasting triglycerides > 2.5 × ULN.
14. Uncontrolled diabetes mellitus as defined by fasting serum glucose > 1.5 × ULN.
15. Patients with bleeding diathesis or on oral anti-vitamin K medication (except low dose warfarin).
16. Patients with a known history of HIV seropositivity.
17. Inability to attend scheduled clinic visits.
18. For the purpose of MRI assessments:
    - Ferromagnetic metal implants other than those approved as safe for use in MR scanner (e.g., braces, some types of aneurysm clips, shrapnel)
    - Patients suffering from uncontrollable claustrophobia or physically unable to fit into the machine (e.g., obesity, etc).

Note: patients with vagal nerve stimulators are permitted to have CT assessments of angiomyolipoma unless local or national regulations do not permit this.

19. Serum creatinine > 1.5 × ULN.
20. History of malignancy in the past two years, other than squamous or basal cell skin cancer.
21. Any severe and/or uncontrolled medical conditions which could cause unacceptable safety risks or compromise compliance with the protocol, such as:
    - ≥ Grade 3 hypercholesterolemia/hypertriglyceridemia or ≥ Grade 2 hypercholesterolemia/hypertriglyceridemia with history of coronary artery disease (despite lipid-lowering treatment if given)
    - Impairment of gastrointestinal function or gastrointestinal disease that may significantly alter the absorption of study drug (e.g., ulcerative disease, uncontrolled nausea, vomiting, diarrhea, malabsorption syndrome).
    - Active skin, mucosa, ocular or GI disorders of Grade > 1

5.2.1 Non interventional follow-up phase/Exclusion criteria

22. Patients who initiate treatment with systemic mTOR inhibitors such as everolimus or rapamycin
23. Patients who have had an embolization immediately after discontinuing study drug
24. Patients who have had a surgical resection of their angiomyolipoma, such as partial/complete nephrectomy immediately after discontinuing study drug
25. Patients who have previously had a kidney CT/MRI performed one-year after everolimus discontinuation (during the follow-up period)

6 Treatment

6.1 Investigational and control drugs

The investigational drug used in the course of this trial is RAD001 (everolimus); the control drug used in this trial is matching placebo.

Definition of terms:

- Study treatment / Study drug = RAD001 or Matching Placebo

In both treatment arms, the study drug will be given by continuous oral daily dosing of two 5-mg tablets.

Medication labels for study drug will comply with the legal requirements of each country and be printed in local language. They will supply no information about the patient. The storage conditions for study drug will be described on the medication label.

6.1.1 Known undesirable effects of study drug/treatment

Adverse events (AEs) most frequently observed with RAD001 are rash, stomatitis/oral mucositis, non-infectious pneumonitis, fatigue, headache, anorexia, nausea, vomiting, diarrhea, asthenia, cough and infections. Overall, the most frequently observed laboratory abnormalities include neutropenia, thrombocytopenia, hypercholesterolemia, anemia, hyperglycemia, lymphopenia, increased creatinine and/or hypertriglyceridemia. The majority of these AEs have been of mild to moderate severity (NCI CTC grade 1-2). Recommendations for dose adjustments, should any of these treatment-related AEs occur, are given in Table 6-1.

Management of infections

RAD001 is an immunosuppressant. Patients taking RAD001 are therefore at an increased risk of infection. In oncology patients, some infections have been severe, and rarely have had a fatal outcome. Physicians should be aware of the increased risk of infection, and should warn patients and their caregivers to be vigilant for signs and symptoms of infection, and to seek medical attention immediately should such signs or symptoms occur. Should an infection occur, anti-infectives should be prescribed as clinically appropriate, and in the case of clinically significant infection, consideration should be given to withholding study medication until resolution of the infection.

Management of stomatitis/oral mucositis/mouth ulcers

Stomatitis/oral mucositis/mouth ulcers due to RAD001 should be treated using appropriate locally available supportive care. Please note that investigators in earlier trials have described the oral toxicities associated with RAD001 as mouth ulcers, rather than mucositis or stomatitis. If your examination reveals mouth ulcers rather than a more general inflammation of the mouth, please classify the adverse event as such. Please follow the paradigm below for treatment of stomatitis/oral mucositis/mouth ulcers:

1. For mild toxicity (grade 1), use conservative measures such as **non-alcoholic mouth wash or salt water (0.9%) mouth wash** several times a day until resolution.
2. For more severe toxicity (grade 2 in which case patients have pain but are able to maintain adequate oral alimentation, or grade 3 in which case patients cannot maintain adequate oral alimentation), the suggested treatments are **topical analgesic mouth treatments (i.e., local anesthetics such as benzocaine, butyl aminobenzoate, tetracaine hydrochloride, menthol, or phenol)** with or without **topical corticosteroids**, such as triamcinolone oral paste 0.1% (i.e., Kenalog in Orabase®).
3. Agents containing hydrogen peroxide, iodine, and thyme derivatives may tend to worsen mouth ulcers. It is preferable to avoid these agents.
4. Antifungal agents must be avoided unless a fungal infection is diagnosed. In particular, systemic imidazole antifungal agents (ketoconazole, fluconazole, itraconazole, etc.) should be avoided in all patients due to their strong inhibition of RAD001 metabolism, therefore leading to higher RAD001 exposures. Therefore, topical antifungal agents are preferred if an infection is diagnosed. Similarly, antiviral agents such as acyclovir should be avoided unless a viral infection is diagnosed.

Management of hyperlipidemia and hyperglycemia

Management of hyperlipidemia should take into account the pre-treatment status and dietary habits of the patient. Blood tests to monitor hyperlipidemia must be taken in the fasting state. Hyperlipidemia and hypertriglyceridemia should be treated according to local best clinical practice. Grade 3 hypercholesterolemia (> 400 mg/dL or 10.34 mmol/L) or grade 3 hypertriglyceridemia (>5 × ULN) should be treated with a 3-hydroxy-3-methyl-glutaryl (HMG)-CoA reductase inhibitor (e.g., atorvastatin, pravastatin) or appropriate lipid-lowering medication, in addition to diet. Patients should be monitored clinically and through serum biochemistry for the development of rhabdomyolysis and other adverse events as required in the product label/data sheets for HMG-CoA reductase inhibitors.

Note: Concomitant therapy with fibrates and an HMG-CoA reductase inhibitor is associated with an increased risk of a rare but serious skeletal muscle toxicity manifested by rhabdomyolysis, markedly elevated creatine phosphokinase levels and myoglobinuria, acute renal failure and sometimes death. The risk versus benefit of using this therapy should be determined for individual patients based on their risk of cardiovascular complications of hyperlipidemia.

Hyperglycemia has been reported in clinical trials. Monitoring of fasting serum glucose is recommended prior to the start of everolimus therapy and periodically thereafter. Optimal glycemic control should be achieved before starting trial therapy.

Management of diarrhea

Diarrhea attributed to RAD001 toxicity may be treated with loperamide. Other medications for diarrhea may be used as needed.

Management of amenorrhea

Investigators should be aware of the identified risk of secondary amenorrhea in post-adolescent females. No changes in study drug treatment or treatment with concomitant medications was implemented in prior cases in this study. Nearly all previous cases of amenorrhea for patients on this study resolved spontaneously. Amenorrhea did not result in any treatment discontinuations. If amenorrhea event of 6 months or more is seen, consultations with an endocrinologist, gynecologist, or other appropriate health care personnel are recommended.

6.1.2 Dosing modifications in case of treatment related toxicities

Table 6-1 and Table 6-2 provide the procedures to be followed for dose modification and re-initiation of study treatment in the event of toxicities suspected to be related to the study treatment.

Table 6-1 Everolimus dose modification guidelines for non-hematologic toxicities

Toxicity	Actions
Pneumonitis	See Table 6-3
Hyperlipidemia and/or hypertriglycerdemia	Any grade:<br>Treat according to best clinical practice. No specific dose reductions are needed
Hyperglycemia	Any grade:<br>Treat according to best clinical practice. No specific dose reductions are needed
Stomatitis	Grade 2:<br>Interrupt study drug until resolution to ≤ grade 1.<br>Restart at the same dose<br>Grade 3:Interrupt study drug until recovery to grade ≤1.<br>Reintroduce study drug at the next lower dose level*<br>Discontinue study drug if stomatitis doesn't recover to ≤ grade 1 within 4 weeks<br>Grade 4:<br>Discontinue study drug.
Other Toxicities	Grade 2 and 3<br>Interrupt administration until resolution to ≤ grade 1.<br>Restart at the same dose.<br>Grade 4<br>Hold study drug until recovery to ≤ grade 1.
Toxicity requiring interruption for ≥6 weeks	Permanently discontinue treatment.
* No specific dose adjustments are recommended for Grade 1 toxicity. However, physicians should always manage patients according to their medical judgment based on the particular clinical circumstances. For dose adjustments for Grade 3, refer to Table 6-4 for next lowest dose level.	

Table 6-2 Dose modification guidelines for hematologic toxicities

Toxicity	Actions
Thrombocytopenia<br>Platelet count	≥ 75000/mm^3: No change<br>50000/mm^3 to 75000/mm^3: Hold study drug until recovery to **≥** 75000/mm^3: Reintroduce study drug at the same dose level<br>< 50000/mm^3<br>Hold study drug until recovery to **≥** 75000/mm^3: Reintroduce everolimus at the next lower dose level**, if available.
Absolute Neutrophil Count (ANC)	≥ 1000/mm^3: No change<br>500/mm^3 to 1000/mm^3: Hold study drug until recovery to ≥ 1000/mm^3: Reintroduce study drug at the same dose level<br>< 500/mm^3: Hold until recovery to ≥ 1000/mm^3. Reintroduce study drug at the next lowest dose level**, if available.
Febrile neutropenia	Hold further dosing until ANC ≥ 1250/mm^3 and no fever.<br>Then resume dosing at the next lower dose level** if available.
Toxicity requiring interruption for ≥ 6 weeks	Discontinue study treatment.
**Physicians should always manage patients according to their medical judgment based on the particular clinical circumstances.	

Management of non-infectious pneumonitis

Non-infectious pneumonitis is a known side effect of rapamycin analogues including RAD001. Clinically significant pneumonitis is typically accompanied by non-specific symptoms including dyspnea, nonproductive cough, fatigue, and fever. Diagnosis is generally suspected in individuals receiving mTOR inhibitors who develop these symptoms or in asymptomatic individuals in whom a routine chest CT scan reveals a new ground glass or alveolar infiltrate. The frequency of symptomatic pulmonary toxicity (all grades) was 14% in a phase III study of RAD001 in patients with metastatic renal cell carcinoma ([CRAD001C2240]). Severe (CTC grade 3) pneumonitis occurred in 4% of patients, and an occasional fatality was reported. The lung toxicity was partly or completely reversible in the majority of cases with interventions including drug interruption, discontinuation and the use of corticosteroids.

Patients with LAM participating in this trial will have pulmonary function tests at baseline. All patients will be routinely questioned throughout the trial as to the presence of new or changed pulmonary symptoms consistent with drug-induced lung toxicity. In addition, individuals with LAM will undergo routine pulmonary function testing at baseline and at 6, 12, 18 and 24 weeks and every 12 weeks thereafter during their study visits. Pulmonary function tests will be performed and interpreted using the ATS/ERS guidelines (Brusasco et al 2005). If non-infectious pneumonitis develops, the guidelines in Table 6-3 should be followed. Dose modification instructions are provided in Table 6-4. Consultation with a pulmonologist is recommended for any case of pneumonitis that develops during the study. CT scans should be performed, as clinically indicated, if there are symptoms that indicate that the patient has developed non-infectious pneumonitis.

Table 6-3 Management of non-infectious pneumonitis

Worst Grade Pneumonitis	Required Investigations	Management of Pneumonitis	Study Treatment Dose Adjustment
Grade 1	CT scans with lung windows. Repeat CT scan at least every 12 weeks until return to within normal limits.	No specific therapy is required	Administer 100% of study treatment dose.
Grade 2	CT scan with lung windows. Consider pulmonary function testing including spirometry, DL_{CO}, and room air O_2 saturation at rest. Repeat CT scan at least every12 weeks until return to within normal limits. Consider bronchoscopy with biopsy and/or BAL	Symptomatic only. Consider corticosteroids if symptoms are troublesome.	Reduce study treatment dose by 1 dose level (see Table 6-4) until recovery to ≤ Grade 1. Study treatment may also be interrupted if symptoms are troublesome. Patients will discontinue study treatment if they fail to recover to ≤ Grade 1 within 3 weeks.
Grade 3	CT scan with lung windows and pulmonary function testing including spirometry, DL_{CO}, and room air O_2 saturation at rest. Repeat at least every 8 weeks until return to within normal limits. Bronchoscopy with biopsy and/or BAL is recommended.	Consider corticosteroids if infective origin is ruled out. Taper as medically indicated.	Hold treatment until recovery to ≤ Grade 1. May restart study treatment within 3 weeks at a reduced dose (by one level) if evidence of clinical benefit.
Grade 4	CT scan with lung windows and required pulmonary function testing including spirometry, DL_{CO}, and room air O_2 saturation at rest. Repeat at least every 8 weeks until return to within normal limits. Bronchoscopy with biopsy and/or BAL is recommended if possible.	Consider corticosteroids if infective origin is ruled out. Taper as medically indicated.	Discontinue treatment.

Follow-up for toxicities

Patients whose treatment is interrupted or permanently discontinued due to an adverse event or abnormal laboratory value suspected to be related to study treatment must be followed at least weekly until the adverse event or abnormal laboratory value resolves or returns to grade 1. If a patient requires a dose delay of ≥ 6 weeks from the intended day of the next scheduled dose, then the patient must be discontinued from the study.

All patients will be followed for adverse events and serious adverse events for 4 weeks (28 days) following the last dose of study drug. Beyond these 28 days, any serious adverse events that are suspected to be related to the study drug and occur within the next 8 weeks (56 days) will also be collected. Any medication/therapy given during these 12 weeks will be recorded on the CRF.

6.1.3 Study drug

The study drug is RAD001 or matching placebo (during blinded phase only).

6.1.3.1 How supplied

Each study site will be supplied by Novartis with study drug in identically-appearing packaging. One component of the packaging has a 2-part label. Each part of this label contains a medication number corresponding to one of the 2 treatment groups. Investigator staff will identify the study drug package to dispense to the patient by logging onto IWRS and obtaining the medication number. Immediately before dispensing the package to the patient, investigator staff will detach the outer part of the label from the packaging and affix it to the source document (Drug Label Form) containing that patient's unique patient number.

For the duration of the trial, RAD001 or matching placebo will be supplied by Novartis at no charge to each study site. In the extension phase of the study, open label drug will be supplied.

6.1.3.2 Preparation and storage

RAD001 is formulated as 5 and 10mg tablets. In this study, it is formulated in 5mg strength tablets, blister-packed under aluminum foil in units of 10 tablets and dosed on a daily basis. Matching placebo will be provided as a matching tablet and will also be blister packed under aluminum foil in units of 10. RAD001 or matching placebo tablets should be opened only at the time of administration as active drug is both hygroscopic and light-sensitive.

6.1.3.3 Active control

Not applicable for this study.

6.2 Treatment arms

There are two treatment arms in this study: RAD001 or matching placebo. Each patient will be randomized into one treatment arm at the start of study.

6.3 Patient numbering

Each patient in the study is uniquely identified by a **9-digit patient number** which is a combination of his/her **4-digit center number** and **5-digit subject number**. The center number is assigned by Novartis to the investigative site. The Novartis assigned 4 digit center number should be entered on the CRF in the field labeled “Center No.” and the investigator assigned 5-digit subject number alone (excluding the leading zeros) should be entered on the CRF in the field labeled “Subject No.”

When the patient has signed the informed consent form, the investigator or his/her staff will log onto IWRS and provide the requested patient identifying information including the patient number. Once assigned to a patient, the patient number will not be reused. If the patient fails to be randomized, a reason why the patient was not randomized should be entered into IWRS as soon as possible. In addition, the Screening Log should be completed for these patients.

Patients who screen fail and are subsequently eligible for trial participation (i.e. now eligible due to a protocol amendment) should be rescreened under the same 9-digit patient number.

Informed consent must be obtained before any testing is performed to determine a patient's eligibility.

6.4 IWRS procedure

User Acceptance Testing of the IWRS based on test data will be performed by the project team prior to its implementation. IWRS will provide contact information and detailed instructions on registration and randomization procedures to each study site. **During the study, a fax confirmation of every transaction of the web-based system will be issued as documentation.**

- At visit 1 (screening/baseline) the investigator will log onto IWRS to register the patient.
- If the patient is eligible to be randomized, just prior to visit 2 (Treatment Day 1), the investigator will log onto IWRS to perform the randomization. **The investigator or his/her designee will log onto IWRS as close as possible to the initiation of therapy (Treatment Day 1, Visit 2).** The investigator will indicate whether the underlying disease is TSC or sporadic LAM and if the patient is taking EIAED then selecting one of the protocol-specified EIAED (see Section 6.5). This information will be used to determine which stratum the patient belongs to in order to assign the correct randomization number.
- If a screened patient fails to be randomized, the IWRS must be updated with this patient's status as soon as possible.
- The investigator or his/her designee will update IWRS immediately if a patient discontinues from the study.
- The investigator or his/her designee will update IWRS for patient unblinding (with respect to emergency unblinding as well as for unblinding following angiomyolipoma progression [i.e., patients who are candidates for open-label RAD001 therapy]).
- No study medication should be dispensed without logging onto IWRS.
- Re-supply of trial therapy to the study sites will occur as needed according to the supply strategy defined in the IWRS (User Requirement Specification).
- During the trial, IWRS will immediately notify the Clinical Trial Head (CTH) or designee and the clinical monitor assigned to the site of any occurrence of emergency code breaks.
- Unblinding may occur after documented angiomyolipoma progression during the blinded treatment phase - this is to enable patients randomized to placebo to switch to open-label RAD001. Unblinding may also occur in the case of medical emergencies when the treating physician believes that the knowledge of the blinded treatment is essential. Complete details are provided in Section 6.7.6 for Emergency unblinding of treatment assignment.

6.5 Treatment assignment

At or prior to Visit 2 (Treatment Day 1), the investigator or his/her designee will log onto IWRS (after verifying that the patient fulfills all eligibility criteria) to randomize the patient. The IWRS will assign a randomization number to the patient, which will be used to link the patient to one of the two treatments, and will specify a unique medication number for the first package of study drug to be dispensed to the patient. The medication number appears on the study medication pack that will be dispensed to the patient. The randomization number will not be communicated to the user.

The randomization will be stratified by one factor with three categories: (1) TSC as underlying disease and patient using enzyme-inducing anti-epileptic drugs (EIAED) at

randomization, (2) TSC as underlying disease and patient not using EIAED at randomization, and (3) sporadic LAM as underlying disease. The following drugs qualify as EIAED(considered strong inducers of CYP3A4):

1. Phenytoin (Dylantin®, Dilantin Kapseals®, Dilantin® Infatabs®, Eptoin®, Epanutin®, Diphenin®, Dipheninum®, Phenytek®)
2. Mephenytoin (Mesantoin®)
3. Carbamazepine (Tegretol®, Biston®, Calepsin®, Carbatrol®, Epitol®, Equetro®, Finlepsin®, Sirtal®, Stazepine®, Telesmin®,Teril®, Timonil®, Trimonil®, Epimaz®, and Degranol®)
4. Phenobarbital (Luminal®)
5. Pentobarbital (Nembutal®)
6. Primidone (Mysoline®)
7. Oxcarbazepine (Trileptal®)

6.6 Treatment blinding

This is a double-blind study. The study design allows for patient unblinding only in very precise circumstances. The clinical personnel at the Central Laboratory and at the Central Radiology will remain blinded to the identity of the treatment from the time of randomization until final database lock.

Randomization will be performed using the following procedures to ensure that treatment assignment is unbiased and concealed as best as possible from all individuals involved in the study: 1) randomization data are kept strictly confidential until the time of unblinding at progression of disease or at time of final analysis, and will not be accessible to anyone involved in the conduct of the study with the exception of the DMC who will review safety data 6 months after the randomization of the first patient and every 6 months after that and, 2) the identity of the treatments will be concealed by the use of study drugs (RAD001 and matching placebo) that are identical in packaging, labeling, schedule of administration and in appearance.

The randomization list will be generated by the IWRS provider using a validated system that automates the random assignment of patient numbers to randomization numbers. The randomization numbers are linked to the two different treatment arms, which in turn are linked to medication numbers. The randomization scheme for patients will be reviewed and approved by a member of the Biostatistics Quality Assurance Group and will be locked and kept by them after its approval.

At the conclusion of the study, when the study data have been verified, the protocol deviations have been determined and the database locked, the assigned blinded drug codes can be broken and made available to the sponsor for the final analysis of the study data.

6.7 Treating the patient

6.7.1 Study drug administration

RAD001 or matching placebo will be dispensed by the study center personnel on an outpatient basis. Patients will be provided with 5 weeks worth of study drug on Treatment Day 1, week 4 and week 8 for self administration at home. At later visits, trial therapy will be

provided as needed. Patients who enter the open label phase of the study will be provided with RAD001, according to the dispensing schedule above, for self-administration at home.

The investigator should instruct the patient to take the study drug exactly as prescribed (promote compliance). All dosages prescribed and dispensed to the patient and all dose changes during the study must be recorded on the Dosage Administration Record CRF.

RAD001 or matching placebo will be dosed starting on Treatment Day 1 (Visit 2). Patients will be instructed to take two 5mg tablets of RAD001 or matching placebo orally once daily at the same time each day immediately after a meal. RAD or matching placebo tablets should be swallowed whole with a glass of water. The tablets should not be chewed or crushed. However, in cases where tablets can not be swallowed, the tablets should be dissolved in water just prior to administration. Approximately 30 ml of water should be put into a glass. The tablets should then be added and the contents stirred gently (for a maximum of 7 minutes) until the tablets disintegrate. The contents should then be drunk by the patient. Afterwards, the glass should be rinsed with an additional 30 ml of liquid and drunk by the patient. If vomiting occurs, no attempt should be made to replace the vomited dose.

On days when blood will be drawn (scheduled visits), patients should **not** take the daily study drug dose **until after** blood is drawn so that an accurate trough level of RAD001 can be obtained. **On days of scheduled visits, patients should bring their daily dose of study drug into the clinic with them for administration after blood is drawn.**

Patients should be requested to bring their unused study drug, including the empty blister packs, to the clinic at each visit. Compliance should be verified by the investigator's staff through counting the number of tablets consumed between visits. The investigator (or his/her designee) will document dosage administration and all dose changes during the study on the Dosage Administration Record CRF. The site must maintain an overall drug accountability log for the study, as well as individual accountability records for each patient. The dose, amount dispensed, amount received, and amount unused must be recorded in the source document. Drug accountability will be noted by the field monitor during site visits and at the completion of the study. The patient will be asked to return all unused RAD001 or matching placebo at each dispensing visit and at the end of the study.

Patients will receive treatment with study drug until angiomyolipoma progression (see definition in Section 7.5.2), the occurrence of unacceptable toxicity, or until the investigator or patient decides that continuation is not in the best interest of the patient. Interruption for toxicity should follow the instructions in Table 6-1, Table 6-2 and Table 6-3.

6.7.2 Permitted study drug adjustments

For patients who are unable to tolerate the protocol-specified dosing schedule, dose adjustments are permitted in order to keep the patient on study drug. The guidelines set forth in Table 6-1, Table 6-2 and Table 6-3 should be followed. In addition, if any surgery is planned, trial therapy should be interrupted one week prior to surgery and should be re-started as soon as possible after wound healing.

If treatment is interrupted due to toxicity, study drug should not be resumed unless recovery to grade ≤1 is achieved in less than 6 weeks. Then it could be reintroduced at the initial dose or a lower dose level depending on the toxicity type and grade (see Table 6-1 and Table 6-2).

These changes must be recorded on the Dosage Administration Record CRF. If treatment is interrupted for 6 weeks or more, the patient should be discontinued from the study.

Avoid the use of strong CYP3A4 inducers. If patient requires co-administration of strong CYP3A4 inducers (i.e., phenytoin, carbamazepine, rifampin, rifabutin, Phenobarbital, St. John's wort), an increase in the dose of RAD001 up to twice the currently used daily dose should be considered, using 5 mg increments. Enzyme induction usually occurs within 7-10 days, therefore RAD001 dose should be increased by only one increment, 7 days after the start of the inducer therapy. If no safety concerns are seen within the next 7 days, the dose can be increased again one additional increment up to the maximum of twice the daily dose used prior to initiation of the strong CYP3A4 inducer.

This dose adjustment of RAD001 is intended to achieve similar AUC to the range observed without inducers. However, there are no clinical data with this dose adjustment in patients receiving strong CYP3A4 inducers. If the strong inducer is discontinued the RAD001 dose should be returned to the dose used prior to initiation of the strong CYP3A4/PgP inducer.

Table 6-4 RAD001 dose levels for dose adjustment

Dose level	Dose and schedule
Starting Dose	10 mg daily
-1 (-50% of starting dose)	5 mg daily
-2 (-50% of dose level -1)	5 mg every other day

If a patient has already decreased 2 dose levels, no further dose reduction is permitted. Patients requiring a further dose reduction from 5 mg every other day will be required to discontinue study treatment.

6.7.3 Other concomitant medications

Patients must be instructed not to take any additional medications (over-the-counter, herbal or other products) during the study without prior consultation with the investigator. All medications taken within 30 days of starting study treatment should be reported on the Concomitant Medications/Significant Non-drug Therapies CRF. **Enzyme-inducing antiepileptic drugs are allowed on this study.**

The following guidelines must be adhered to during the study:

Investigational or commercial anti-proliferative agents other than study drug (including other mTOR inhibitors, e.g., sirolimus, temsirolimus) are prohibited.

Inhibitors of CYP3A4 and/or PgP

- Co-administration with strong inhibitors of CYP3A4 (e.g., ketoconazole, itraconazole, ritonavir) or P-glycoprotein (PgP) should be avoided.
- Co-administration with moderate CYP3A4 inhibitors (e.g., erythromycin, fluconazole) or PgP inhibitors should be used with caution. If patient requires co-administration of moderate CYP3A4 inhibitors or PgP inhibitors, reduce the dose of everolimus to half the currently used dose. Additional dose reductions to every other day may be required to manage toxicities. If the inhibitor is discontinued the everolimus dose should be returned to the dose used prior to initiation of the moderate CYP3A4/PgP inhibitor.
- Seville orange, star fruit, grapefruit and their juices affect P450 and PgP activity. Concomitant use should be avoided.

Inducers of CYP3A4 and/or PgP

- Avoid the use of strong CYP3A4 inducers. If patient requires co-administration of strong CYP3A4 inducers (i.e., phenytoin, carbamazepine, rifampin, rifabutin, phenobarbital, St. John’s wort), an increase in the dose of everolimus up to twice the currently used daily dose should be considered, 5mg increments. Enzyme induction usually occurs within 7-10 days, therefore everolimus dose should be increased by one increment 7 days after the start of the inducer therapy. If no safety concerns are seen within the next 7 days, the dose can be increased again one additional increment up to a maximum of twice the daily dose used prior to initiation of the strong CYP3A4 inducer.
- This dose adjustment of everolimus is intended to achieve similar AUC to the range observed without inducers. However, there are no clinical data with this dose adjustment in patients receiving strong CYP3A4 inducers. If the strong inducer is discontinued the everolimus dose should be returned to the dose used prior to initiation of the strong CYP3A4/PgP inducer.

Table 6-5 lists clinically relevant CYP3A inhibitors inducers and the definition of strong and moderate inhibitors/inducers.

RAD001 may affect a patient’s response to vaccinations making the response less effective. Live vaccines and close contact with those who have received live vaccines should be avoided while a patient is treated with RAD001.

Otherwise, the use of other concomitant medication/therapy deemed necessary for the care of the patient is allowed. The investigator should instruct the patient to notify the study site about any new medications he/she takes after the start of the study drug. All medications (other than study drug) and significant non-drug therapies (including physical therapy and blood transfusions) administered after the patient starts study drug and for up to 8 weeks (56 days) after study drug discontinuation must be listed on the Concomitant Medications/Significant Non-drug Therapies CRF.

Table 6-5 Clinically relevant drug interaction: inducers and inhibitors of isoenzyme CYP3A

INDUCERS
**Strong inducers**:<br>avasimibe, carbamazepine, mitotane, phenobarbital, phenytoin, rifabutin, rifampin (rifampicin), St. John's wort (hypericum perforatum)<br>**Moderate inducers**:<br>bosentan, efavirenz, etravirine, genistein, modafinil, nafcillin, ritonavir, [talviraline], thioridazine, tipranavir
**INHIBITORS**
**Strong inhibitors:**<br>boceprevir, clarithromycin, cobicistat, conivaptan, elvitegravir, indinavir, itraconazole, ketoconazole, lopinavir, mibefradil, nefazodone, nelfinavir, posaconazole (Krishna et al 2009), ritonavir, saquinavir, telaprevir, telithromycin, tipranavir, troleandamycin, voriconazole<br>**Moderate inhibitors:**<br>Amprenavir, aprepitant, atazanavir, casopitant, cimetidine, ciprofloxacin, cyclosporine, darunavir, diltiazem, dronedarone, erythromycin, fluconazole, fosamprenavir, grapefruit juice (citrus parasidi fruit juice), imatinib, schisandra sphenanthera, tofisopam, verapamil

Table 6-6 Clinically relevant drug interactions mediated by PgP

SUBSTRATES
colchicine, digoxin, fexofenadine, indinavir, paclitaxel, talinolol, topotecan, vincristine
**INDUCERS**
rifampin, St John’s wort
**PgP INHIBITORS and PgP/CYP3A DUAL INHIBITORS**
amiodarone, azithromycin, captopril, carvedilol, clarithromycin, conivaptan, diltiazem, dronedarone, elacridar, erythromycin, felodipine, fexofenadine, fluvoxamine, ginkgo (ginkgo biloba), indinavir, itraconazole, lopinavir, mibefradil, milk thistle (silybum marianum), nelfinavir, nifedipine, nitrendipine, paroxetine, quercetin, quinidine, ranolazine, rifampin, ritonavir, saquinavir, Schisandra chinensis, St John’s wort (hypericum perforatum), talinolol, Telaprevir, telmisartan, ticagrelor, tipranavir, tolvaptan, valspodar, verapamil
Reference: Internal Clinical Pharmacology Drug-drug interaction (DDI) memo, updated Oct. 2, 2011, which summarizes DDI data from three sources including the FDA’s “Guidance for Industry, Drug Interaction Studies”, the University of Washington’s Drug Interaction Database, and Indiana University School of Medicine's Drug Interaction Table.

6.7.4 Study drug interruption or discontinuation

The term “interruption” refers to a patient stopping the study medication during the course of the study, but then re-starting it at a later time in the study.

The term “discontinuation” refers to a patient’s premature withdrawal from the study treatment. The reason for discontinuation from treatment will be recorded on the End of Treatment CRF. The patient may discontinue participation in the study for any of the following reasons:

1. adverse event(s)
2. abnormal laboratory value(s)
3. abnormal test procedure result(s)
4. angiomyolipoma progression (defined in Section 7.5.2)
5. protocol deviation
6. subject withdrew consent
7. lost to follow-up

8. administrative problems
9. death
10. new treatment for indication under study
11. treatment duration completed as per protocol (only to be used at end of extension phase)
12. final primary analysis (only to be used when results of final analysis are known and the decision is made on whether to launch the extension phase)

If a patient has discontinued the study drug due to an unacceptable adverse event or an abnormal laboratory value, he/she should not have withdrawal of consent recorded as the reason for discontinuation. Instead, the reason for discontinuation must be recorded as due to an AE or abnormal laboratory value.

Patients who discontinue the study drug regardless of the reason must have end of treatment evaluations (Refer to Table 7-1, and Table 7-2, End of Treatment) on the day of study treatment discontinuation or within 1 week of study treatment discontinuation. The investigator or his/her designee will proceed as follows:

- Update IWRS immediately with any patient discontinuations.
- Complete the end of treatment evaluations (additional details are provided in Table 7-1, and Table 7-2) and the End of Treatment CRF indicating the date and reason for stopping the study drug.
- All patients will have a follow-up visit 4 weeks (28 days) after the last dose of the study treatment. During this visit, AE and SAE information will be collected and recorded on the appropriate CRFs. Patients will also be followed for an additional 8 weeks (56 days) for collection and recording of any SAEs that are suspected to be related to study drug. In addition, any medication/therapy taken during these 8 weeks (56 days) will be recorded on the **Concomitant medications/Significant non-drug therapies CRF page**. If the patient is unable to return to the clinic, the investigator or his/her designee will contact the patient or caregiver via telephone to collect this information.
- All patients who are discontinued from study treatment for any reason other than angiomyolipoma progression will continue to have kidney CT/MRIs performed annually until eventual angiomyolipoma progression, the start of any non-study systemic anti-angiomyolipoma therapy, withdrawal of consent, or until end of the study, whichever occurs first. Radiological studies will be sent for central review during this follow-up period. The investigator or his/her designee will collect information on the initiation of additional anti-angiomyolipoma therapies every month. This information may be obtained during a telephone call and will be recorded in the source documents as well as on the Non-Study Systemic Anti-angiomyolipoma Therapy CRF page.

All patients must have evaluations for 4 weeks (28 days) after the last dose of study treatment (and an additional 8 weeks (56 days) for collection and recording of any SAEs that are suspected to be related to study drug). Patients lost to follow up should be recorded as such on the End of Treatment CRF. Patients who require angiomyolipoma-related surgery should discontinue the study drug before the surgery. Patients who discontinue study drug before completing the study should be scheduled for a visit as soon as possible, at which time all of the assessments listed for End of Treatment visit will be performed. At a minimum, all

patients who discontinue study treatment, including those who refuse to return for a final visit, will be contacted for safety evaluations at 12 weeks (84 days) after the last dose of study drug.

6.7.5 Withdrawal from the study and study evaluation completion

Patients **may** voluntarily withdraw from the study or be dropped from it at the discretion of the investigator at any time.

As a general rule, if a patient discontinues study drug and later is withdrawn from the study, the reasons for study evaluation completion may include the following:

- Protocol deviation
- Subject withdrew consent
- Lost to follow-up
- Administrative problems
- Follow-up phase completed as per protocol

  This reason would be selected in any of the following scenarios:
  - A patient has not yet progressed at the time of final results, and if the study is negative and no extension phase will be launched.
  - A patient who has not progressed at the end of the extension phase.
  - A patient who progressed on treatment and would be completed at the 28-day safety visit.
- Death
- New treatment for indication under study
- Angiomyolipoma progression (defined in Section 7.5.2)

For patients who are lost to follow-up, the investigator should show due diligence by recording in the source documents steps taken to contact the patient, e.g., dates of telephone calls, registered letters, etc.

6.7.6 Emergency unblinding of treatment assignment

In general, circumstances that might lead to emergency unblinding are rare. Most often, study drug discontinuation and knowledge of the possible treatment assignments are sufficient to treat a study patient who presents with an emergency condition. One unusual circumstance in which unblinding might be necessary is when a patient requires emergency surgery and the anesthesiologist needs to know all medications that the patient has been exposed to in order to make proper decisions about treatment and support during the surgery.

Emergency unblinding should only be done when necessary in order to treat the patient. Emergency code breaks are performed using IWRS. When the investigator telephones the system to unblind a patient, he/she must provide the requested patient identifying information. The investigator will then receive details of the drug treatment for the specified patient and a fax confirming this information. The system will automatically inform the Novartis monitor for the site and the Clinical Trial Head that the code has been broken.

It is the investigator’s responsibility to ensure that there is a procedure in place to allow access to IWRS in case of emergency. The investigator will inform the patient how to contact his/her

backup in cases of emergency when he/she is unavailable. Study drug must be discontinued after emergency unblinding. The investigator is not allowed to place emergency unblinded patients into open-label RAD001 therapy.

6.7.7 Treatment compliance

Compliance will be assessed by the investigator or his/her designee at each visit using pill counts. This information should be captured in the source document at each visit.

- Patients will be requested to bring their unused medication including empty packaging to the clinic at each visit.
- All doses taken by the patient and all dose changes during the study must be recorded on the Dosage Administration Record CRF.
- The investigator or his/her designee must keep documentation (overall drug accountability log for the study as well as individual study drug accountability records for each patient) of tablets administered, tablets used, dose changes, dates dispensed and intervals between visits.
- Drug accountability will be monitored by the field monitor during site visits and at the completion of the study.

6.7.8 Non interventional follow-up phase after the end of the extension phase

Patient who discontinue everolimus for reasons other than angiomyolipoma progression, and who do not plan to receive treatment with a systemic mTOR inhibitor, will be asked to participate in this long-term follow-up which aims to determine the angiomyolipoma characterizations for up to one-year after permanently discontinuing study medication.

7 Visit schedule and assessments

Table 7-1, Table 7-2, and Table 7-3 list all of the assessments and indicates with an “X” the visits when they are to be performed. All data obtained from these assessments must be supported in the patient’s source documentation. The table indicates which data are entered into the database (D) or remain in source documents only (S).

Tests, procedures and visits should occur on schedule whenever possible. However, tests, procedures, and visits that occur within the prescribed allowable windows indicated in Table 7-1, Table 7-2, and Table 7-3 will not constitute protocol deviations. Please note that labs done at screening/baseline do not need to be repeated on Treatment Day 1, unless Treatment Day 1 occurs more than 14 days after the Screening visit, in which case all Screening/Baseline labs will need to be repeated.

Table 7-1 Blinded phase visit evaluation schedule

Note: To be followed for all patients at the beginning of the study until angiomyolipoma progression or discontinuation

Assessment	Screening/ Baseline	Treatment Day 1	2 weeks (±2 Days)	4 weeks (±2 Days)	6 weeks[†] (±2 Days)	8 weeks (±2 Days)	12 weeks (± 7 Days)	18 weeks (± 7 Days)	24 weeks (± 7 Days)	Every 4 weeks thereafter (± 7 Days)	Every 12 weeks thereafter (± 7 Days)	Every 24 weeks thereafter (± 7 Days)	End of Treatment*(28 days after last dose)	Follow-Up	Study Evaluation Completion
**Time point (days)**	**-21 to -1**	**1**	**14**	**28**	**42**	**56**	**84**	**126**	**168**						**Last**
**Visit no.**	**1**	**2**	**3**	**4**	**5**	**6**	**7**	**8**	**9**				**777 or 779**	**501**	**780[y]**
Written Informed Consent (S)	X														
Inclusion/Exclusion Criteria (D)	X	X													
IWRS Registration [a] (S)	X														
Demography (D)	X														
Medical History/Current Medical Conditions (D)	X	X													
CT/MRI of Kidney[b] (D)	X						X		X**				X[α]	X	
HIV history (D)	X	X													
HBV-DNA, HBsAg, HBs Ab, HBc Ab, HCV-RNA-PCR[∞] (D)	X														
HCV RNA-PCR[§] (D)						X		X			X				

**Assessment**	**Screening/ Baseline**	**Treatment Day 1**	**2 weeks (±2 Days)**	**4 weeks (±2 Days)**	**6 weeks[†] (±2 Days)**	**8 weeks (±2 Days)**	**12 weeks (± 7 Days)**	**18 weeks (± 7 Days)**	**24 weeks (± 7 Days)**	**Every 4 weeks thereafter (± 7 Days)**	**Every 12 weeks thereafter (± 7 Days)**	**Every 24 weeks thereafter (± 7 Days)**	**End of Treatment*(28 days after last dose)**	**Follow-Up**	**Study Evaluation Completion**
**Time point (days)**	**-21 to -1**	**1**	**14**	**28**	**42**	**56**	**84**	**126**	**168**						**Last**
**Visit no.**	**1**	**2**	**3**	**4**	**5**	**6**	**7**	**8**	**9**				**777 or 779**	**501**	**780[y]**
Serum Pregnancy test[d] (D)	X												X[d]		X[d]
Urine pregnancy test[d] (D)		X		X		X	X		X	X	X				
IWRS Randomization[e] (S)		X													
Vital Signs (D)	X	X		X		X	X	X	X		X		X		
Physical exam (including Neurological Exam)[f] (S)	X	X		X		X	X	X	X		X		X		
WHO Performance Status (D)	X	X		X		X	X	X	X		X		X		
ECG[g] (D)	X												X		
Hematology[h] (D)	X	X***	X	X	X	X	X	X	X		X		X		
Endocrine Testing[i] (D)	X								X		X		X		
Fasting Coagulation Studies (PTT/INR) (D)	X	X***					X		X		X				
Fasting Serum Chemistry[j] (D)	X	X***	X	X	X	X	X	X	X		X		X		
Fasting Serum Lipid Profile [k] (D)	X	X***					X		X		X				
Urinalysis[l] (D)	X	X***		X		X	X	X	X		X		X		

Assessment	Screening/ Baseline	Treatment Day 1	2 weeks (±2 Days)	4 weeks (±2 Days)	6 weeks[†] (±2 Days)	8 weeks (±2 Days)	12 weeks (± 7 Days)	18 weeks (± 7 Days)	24 weeks (± 7 Days)	Every 4 weeks thereafter (± 7 Days)	Every 12 weeks thereafter (± 7 Days)	Every 24 weeks thereafter (± 7 Days)	End of Treatment*(28 days after last dose)	Follow-Up	Study Evaluation Completion
**Time point (days)**	**-21 to -1**	**1**	**14**	**28**	**42**	**56**	**84**	**126**	**168**						**Last**
**Visit no.**	**1**	**2**	**3**	**4**	**5**	**6**	**7**	**8**	**9**				**777 or 779**	**501**	**780[y]**
Prior/Current concomitant medications[m] (D)	X	X	X	X	X	X	X	X	X		X		X	X	X
Adverse events[n] (D)	X	X	X	X	X	X	X	X	X		X		X		
Serious Adverse Events[n] (D)	X	X	X	X	X	X	X	X	X		X		X	X	X
Dispense Study Drug[o] (S)		X		X		X	X	X	X		X				
Study Drug Compliance[p] (S)			X	X	X	X	X	X	X		X		X		
Biomarkers Blood Sampling[q] (D)	X			X			X		X		X[q]		X		
TSC1 and *TSC2* Genetic Analysis (D)	X														
PK blood Sampling[r] (D)			X	X			X		X[r]						
Pulmonary Function Tests for LAM Patients Only[s] (D)	X														
Digital Photographs of Skin Lesions[u] (D)	X						X		X		X		X		

Assessment	Screening/ Baseline	Treatment Day 1	2 weeks (±2 Days)	4 weeks (±2 Days)	6 weeks[†] (±2 Days)	8 weeks (±2 Days)	12 weeks (± 7 Days)	18 weeks (± 7 Days)	24 weeks (± 7 Days)	Every 4 weeks thereafter (± 7 Days)	Every 12 weeks thereafter (± 7 Days)	Every 24 weeks thereafter (± 7 Days)	End of Treatment*(28 days after last dose)	Follow-Up	Study Evaluation Completion
**Time point (days)**	**-21 to -1**	**1**	**14**	**28**	**42**	**56**	**84**	**126**	**168**						**Last**
**Visit no.**	**1**	**2**	**3**	**4**	**5**	**6**	**7**	**8**	**9**				**777 or 779**	**501**	**780[y]**
Physician's Global Assessment of Skin Lesions (D)							X		X		X		X		
Pregnancy History, Menstrual History and Monitoring[w] (D)										X	X				
Chest CT[x] (D)		Only to be done if clinically indicated													

Visits 3, 4, 5 and 6 must be completed within ± 2 days of the scheduled visit. All other on-treatment visits with the exception of Visit 2, must be completed within ± 7 days of scheduled visit. The screening/baseline visit must be conducted within 21 days prior to Treatment Day 1. All tests and procedures (i.e., CT/MRI's, hematology labs, biochemistry labs) that occur within the allotted windows will not constitute a protocol deviation.

*****Patients without angiomyolipoma progression at time of discontinuation of study treatment will be followed with CT/MRI tumor assessments annually until eventual angiomyolipoma progression, start of any non-study systemic anti-angiomyolipoma therapy, or until end of the study, whichever comes first.

**CT/MRI of the kidneys will be completed for all patients at baseline, at 12, 24 and 48 weeks after the start of study treatment, and annually thereafter. For each patient, the same imaging modality must be used throughout the trial.

***Screening safety laboratories (biochemistry, hematology, lipid panel, urinalysis and coagulation) only need to be repeated if they were collected more than 14 days prior toTreatment Day 1.

∞Only patients who meet the specified criteria specified in Section 4.1 for Hepatitis B and C testing will require these tests. Any patients who tests positive must be excluded with the exception of Hepatitis B antibody positives caused by prior vaccination.

§ Patients with positive to Hepatitis C antibody or a history of past infection, even if treated and considered 'cured' and does not meet the criteria for reactivation according to Table 4-3, should be followed by HCV-RNA PCR according to visit schedule above.

α A CT/MRI of the kidney would be repeated at the End of Treatment visit if the patient has discontinued for reasons other than radiological progression and it has been more than 8 weeks since their most recent scan during the first year of treatment of more than 6 months since their most recent scan thereafter.

† For Visit 5 (6 week visit), patients residing in the United States for whom travel is difficult, who have tolerated study medication and have no adverse events may, at the discretion of the investigator, attend a local laboratory affiliated with EXAM **One** for blood draws (if such facility is available) and have a telephone consultation with the investigator rather than a clinic visit.

a. Patients should be registered through IWRS after informed consent is signed.

b. CT/MRI of kidney should be performed at screening for all patients. CT/MRI of the kidney should be repeated at 12, 24 and 48 weeks, and annually thereafter, unless observation of response warrants a confirmation with a second scan approximately 12 weeks later (and no sooner than 8 weeks later). For patients who discontinue

Assessment	Screening/ Baseline	Treatment Day 1	2 weeks (±2 Days)	4 weeks (±2 Days)	6 weeks† (±2 Days)	8 weeks (±2 Days)	12 weeks (± 7 Days)	18 weeks (± 7 Days)	24 weeks (± 7 Days)	Every 4 weeks thereafter (± 7 Days)	Every 12 weeks thereafter (± 7 Days)	Every 24 weeks thereafter (± 7 Days)	End of Treatment*(28 days after last dose)	Follow-Up	Study Evaluation Completion
**Time point (days)**	**-21 to -1**	**1**	**14**	**28**	**42**	**56**	**84**	**126**	**168**						**Last**
**Visit no.**	**1**	**2**	**3**	**4**	**5**	**6**	**7**	**8**	**9**				**777 or 779**	**501**	**780[y]**

due to angiomyolipoma progression, as verified by the central radiologist, an additional CT/MRI does not need to be completed at the End of Treatment visit. **CT/MRI of kidney is not required at 18 weeks. For each patient**, the same imaging modality must be used throughout the trial.

c.

d. Serum Pregnancy test only to be performed in females of childbearing potential at central lab. A serum pregnancy test will be conducted at baseline and at either end of treatment or end of study (depending upon the reason for discontinuing study medication) in all females of child-bearing potential. After Protocol Amendment 3 implementation, a urine pregnancy test will be repeated every 4 weeks at patient's home according to study visit schedule until study drug is discontinued. Urine pregnancy test will be conducted at clinical site in place of patient's home when a visit is scheduled. Results of at home urine pregnancy test will be recorded in patient diaries for source documentation only.

e. Patients should be randomized through IWRS, after all eligibility criteria have been confirmed.

f. Significant findings from physical and neurological exam will be noted on the Relevant Medical History and/or Adverse Events CRF pages.

g. ECG may be repeated at the investigator's discretion if there are signs or symptoms of cardiotoxicity. Significant findings will be noted on the Relevant Medical History CRF pages (if present before treatment) or Adverse Events CRF pages (if after treatment has started).

h. Hematology must include: hemoglobin, hematocrit, platelets, red blood cell count (RBC), total white blood cell count (WBC), absolute & differential including neutrophils, lymphocytes, monocytes, eosinophils and basophils. Absolute Neutrophil Count (ANC) will be calculated by the laboratory.

i. Assessment of hormones (FSH, LH and testosterone in all patients, estradiol in all women) at baseline and every 12 weeks after the start of study drug.

j. Fasting serum chemistry must include: total LDH, fasting glucose, sodium, magnesium, phosphate, potassium, chloride, bicarbonate, creatinine, BUN, albumin, total protein, SGOT (AST), SGPT (ALT), total bilirubin, alkaline phosphatase, uric acid, calcium.

k. Fasting serum lipid profile must include: total cholesterol, triglycerides, LDL, and HDL.

l. Standard urinalysis dipstick assessment must include: pH, protein, glucose, blood, ketones, and leukocytes.

m. Concomitant medications taken within the 30 days prior to starting treatment and up to 4 weeks (28 days) after last dose should be documented on the appropriate CRF.

n. AEs should be recorded on the Adverse Event CRF page from the time of starting study treatment and up to 4 weeks (28 days) after last dose (until the follow-up visit). All SAEs occurring within 28 days of study treatment discontinuation (until the follow-up visit), regardless of causality, should be captured on the Adverse Event CRF page. SAEs with suspected causality to study drug should be captured for an additional 8 weeks (56 days) after follow-up visit (for a total of 12 weeks (84 days) after treatment discontinuation).

o. Study drug will be dispensed at the indicated visits. At each dispensing visit, site personnel will log into IWRS to obtain the patient's study drug assignment to last until the next dispensing visit including sufficient overage.

p. At each study visit, site personnel will review the patient's returned study drug, foil packs (used and unused) to ensure the patient is compliant.

q. During the blinded treatment phase, a biomarker sample will be collected at screening/baseline, at 4, 12, 24, 36, and 48 weeks and at end of treatment. **No additional**

Assessment	Screening/ Baseline	Treatment Day 1	2 weeks (±2 Days)	4 weeks (±2 Days)	6 weeks[†] (±2 Days)	8 weeks (±2 Days)	12 weeks (± 7 Days)	18 weeks (± 7 Days)	24 weeks (± 7 Days)	Every 4 weeks thereafter (± 7 Days)	Every 12 weeks thereafter (± 7 Days)	Every 24 weeks thereafter (± 7 Days)	End of Treatment*(28 days after last dose)	Follow-Up	Study Evaluation Completion
Time point (days)	-21 to -1	1	14	28	42	56	84	126	168						Last
Visit no.	1	2	3	4	5	6	7	8	9				777 or 779	501	780[y]

biomarker samples will be collected.

r. During the blinded treatment phase, blood samples for RAD001 levels will be collected from all patients pre-dose, and at 2.0 hours (± 30 mins) after dosing at 2, 4, 12, 24 and 48 weeks.

s. **For patients with skin lesions at screening/baseline**: Skin lesions will be photographed using a digital camera and measured using a disposable ruler. Photographs will be taken at baseline, at 12 and 24 weeks after start of study treatment, and **every 12 weeks thereafter and end of treatment**. A confirmatory skin lesion assessment should be performed approximately 12 weeks later (and no sooner than 8 week later) after the first assessment of response.

t. Additional menstrual history (age at menarche, previous cases of amenorrhea or menstrual disorders, biological mother's age at menopause) and pregnancy history (Pregnancies, full-term gestations, abortions, live births, miscarriages) will be collected one time after implementation of protocol amendment 3. Following implementation of amendment 3, menstrual status will be collected monthly via patient diary for source documentation. Every 12 weeks, data from patient diary will be collected at clinical site for CRF. It is recommended that study coordinators contact patients monthly for the first 3 months to remind patients to document menstrual status in patient diary.

u. Chest CT should be performed as clinically indicated.

NOTE: Crossover (Open-label phase only): Assessments for safety and efficacy are to occur at the same intervals as for the blinded treatment phase of trial. However, PK and biomarker pharmacodynamics studies will NOT be performed. Radiological evaluations will continue to be sent for central review during the open-label phase.

v. The total amount of blood to be taken at each visit is outlined in the central [Laboratory Manual] for instructions on collecting blood samples. CRF for Study Evaluation Completion will only be completed at the end of all study-related activity, including any long-term follow-up assessments when the patient is not taking study drug.

Table 7-2 Open label and extension phase visit evaluation schedule

Note: To be followed for all patients who are initiating RAD001 for the first time:

Assessment	Baseline	Treatment Day 1	2 weeks (±2 Days)	4 weeks (±2 Days)	6 weeks[†] (±2 Days)	8 weeks (±2 Days)	12 weeks (± 7 Days)	18 weeks (± 7 Days)	24 weeks (± 7 Days)	Every 4 weeks thereafter (± 7 Days)	Every 12 weeks thereafter (± 7 Days)	Every 24 weeks thereafter (± 7 Days)	End of Treatment*(28 days after last dose)	Follow-Up	Study Evaluation Completion
Time point (days)	-21 to -1	1	14	28	42	56	84	126	168						Last

**Visit no.**	**101**	**102**	**103**	**104**	**105**	**106**	**107**	**108**	**109**				**778 or 779**	**501**	**780**[t]
CT/MRI of Kidney[a] (D)	X						X		X**				X[α]		
Vital Signs (D)	X	X		X		X	X	X	X		X		X		
Physical exam (including Neurological Exam)[c] (S)	X	X		X		X	X	X	X		X		X		
WHO Performance Status (D)	X	X		X		X	X	X	X		X		X		
ECG[d] (D)	X												X		
HCV RNA-PCR[§] (D)						X		X			X				
Hematology[e] (D)	X	X***	X	X	X	X	X	X	X		X		X		
Endocrine Testing[f] (D)	X								X		X		X		
Fasting Coagulation Studies (PTT/INR) (D)	X	X***					X		X		X				
Fasting Serum Chemistry[g] (D)	X	X***	X	X	X	X	X	X	X		X		X		
Fasting Serum Lipid Profile [h] (D)	X	X***					X		X		X				
Urinalysis[i] (D)	X	X***		X		X	X	X	X		X		X		
Urine pregnancy test[€] (D)		X		X		X	X		X	X					
Serum Pregnancy Test[€] (D)													X[€]		X[€]
Prior/Current concomitant medications[j] (D)	X	X	X	X	X	X	X	X	X		X		X	X	X

Assessment	Baseline	Treatment Day 1	2 weeks (±2 Days)	4 weeks (±2 Days)	6 weeks† (±2 Days)	8 weeks (±2 Days)	12 weeks (± 7 Days)	18 weeks (± 7 Days)	24 weeks (± 7 Days)	Every 4 weeks thereafter (± 7 Days)	Every 12 weeks thereafter (± 7 Days)	Every 24 weeks thereafter (± 7 Days)	End of Treatment*(28 days after last dose)	Follow-Up	Study Evaluation Completion
**Time point (days)**	**-21 to -1**	**1**	**14**	**28**	**42**	**56**	**84**	**126**	**168**						**Last**
**Visit no.**	**101**	**102**	**103**	**104**	**105**	**106**	**107**	**108**	**109**				**778 or 779**	**501**	**780[t]**
Adverse events[k] (D)	X	X	X	X	X	X	X	X	X		X		X		
Serious Adverse Events[k] (D)	X	X	X	X	X	X	X	X	X		X		X	X	X
Dispense Study Drug[l] (S)		X		X		X	X	X	X		X				
Study Drug Compliance[m] (S)			X	X	X	X	X	X	X		X		X		
Digital Photographs of Skin Lesions[p] (D)	X						X		X		X		X		
Physician's Global Assessment of Skin Lesions (D)							X		X		X		X		
Pregnancy History, Menstrual History and Monitoring[r] (D)										X	X				
Chest CT Scan[s] (D)		Only to be done if clinically indicated													

Visits 103, 104, 105 and 106 must be completed within ± 2 days of the scheduled visit. All other visits with the exception of Visit 102, must be completed within ± 7 days of scheduled visit. The screening/baseline visit is taken as the last visit from the blinded treatment phase and must be conducted prior to the Open Label Treatment Day 1. All tests and procedures (i.e., CT/MRI's, hematology labs, biochemistry labs) that occur within the allotted windows will not constitute a protocol deviation.

*Patients without angiomyolipoma progression at time of discontinuation of open label RAD001 will be followed with CT/MRI tumor assessments annually until eventual angiomyolipoma progression, start of any non-study systemic anti-angiomyolipoma therapy, or until end of the study, whichever comes first.

Assessment	Baseline	Treatment Day 1	2 weeks (±2 Days)	4 weeks (±2 Days)	6 weeks† (±2 Days)	8 weeks (±2 Days)	12 weeks (± 7 Days)	18 weeks (± 7 Days)	24 weeks (± 7 Days)	Every 4 weeks thereafter (± 7 Days)	Every 12 weeks thereafter (± 7 Days)	Every 24 weeks thereafter (± 7 Days)	End of Treatment*(28 days after last dose)	Follow-Up	Study Evaluation Completion
**Time point (days)**	**-21 to -1**	**1**	**14**	**28**	**42**	**56**	**84**	**126**	**168**						**Last**
**Visit no.**	**101**	**102**	**103**	**104**	**105**	**106**	**107**	**108**	**109**				**778 or 779**	**501**	**780**[t]

**CT/MRI of the kidneys will be completed for all patients at baseline, at 12, 24 and 48 weeks after the start of open label RAD001and annually thereafter.

***All open label baseline laboratories must be collected within 14 days of Open Label Treatment Day 1.

§ Patients with positive to Hepatitis C antibody or a history of past infection, even if treated and considered ‘cured’ and does not meet the criteria for reactivation according to Table 4-3, should be followed by HCV-RNA PCR according to visit schedule above.

€ A urine pregnancy test will be conducted locally at baseline in all females of child-bearing potential. After Protocol Amendment 3 implementation, a urine pregnancy test will be repeated every 4 weeks at patient’s home according to study visit schedule until study drug is discontinued. Urine pregnancy test will be conducted at clinical site in place of patient’s home when a visit is scheduled. A serum pregnancy test will be conducted at either end of treatment or end of study (depending upon the reason for discontinuing study medication). Results of at home urine pregnancy test will be recorded in patient diaries for source documentation only.

† For Visit 105 (6 week visit), patients residing in the United States for whom travel is difficult, who have tolerated study medication and have no adverse events may, at the discretion of the investigator, attend a local laboratory affiliated with EXAM **One** for blood draws (if such facility is available) and have a telephone consultation with the investigator rather than a clinic visit.

α A CT/MRI of the kidney would be repeated at the End of Treatment visit if the patient has discontinued for reasons other than angiomyolipoma progression and it has been more than 8 weeks since their most recent scan during the first year of treatment or more than 6 months since their most recent scan thereafter.

a. For all patients, CT/MRI of kidneys should be repeated at 12, 24 and 48 weeks after the start of open label treatment, and annually thereafter unless observation of angiomyolipoma response warrants a confirmation approximately 12 weeks later (and no sooner than 8 weeks later). CT/MRI of the kidneys is not required at 18 weeks. For each patient, the same imaging modality must be used throughout the trial.

b. Significant changes from last Physical and Neurological exam conducted in the blinded phase will be on the Relevant Medical History and/or Adverse Events CRF pages.

c. ECG may be repeated at the investigator’s discretion if there are signs or symptoms of cardiotoxicity. Significant findings will be noted on the Relevant Medical History CRF pages (if present before open-label RAD001) or Adverse Events CRF pages (if after open-label RAD001 has started).

d. Hematology must include: hemoglobin, hematocrit, platelets, red blood cell count (RBC), total white blood cell count (WBC), absolute & differential including neutrophils, lymphocytes, monocytes, eosinophils and basophils. Absolute Neutrophil Count (ANC) will be calculated by the laboratory.

e. Assessment of hormones (FSH, LH and testosterone in all patients; estradiol in all women) at baseline (using the latest assessment from the blinded treatment phase) and every 12 weeks after the start of open-label RAD001.

f. Fasting serum chemistry must include: total LDH, fasting glucose, sodium, magnesium, phosphate, potassium, chloride, bicarbonate, creatinine, BUN, albumin, total protein, SGOT (AST), SGPT (ALT), total bilirubin, alkaline phosphatase, uric acid, calcium.

g. Fasting serum lipid profile must include: total cholesterol, triglycerides, LDL, and HDL. Assessment should be repeated every 12 weeks.

h. Standard urinalysis dipstick assessment must include: pH, protein, glucose, blood, ketones, and leukocytes.

i. Concomitant medications taken within the 30 days prior to starting open-label RAD001 and up to 4 weeks (28 days) after the last dose of open-label RAD001 should be documented on the appropriate CRF.

Assessment	Baseline	Treatment Day 1	2 weeks (±2 Days)	4 weeks (±2 Days)	6 weeks[†] (±2 Days)	8 weeks (±2 Days)	12 weeks (± 7 Days)	18 weeks (± 7 Days)	24 weeks (± 7 Days)	Every 4 weeks thereafter (± 7 Days)	Every 12 weeks thereafter (± 7 Days)	Every 24 weeks thereafter (± 7 Days)	End of Treatment*(28 days after last dose)	Follow-Up	Study Evaluation Completion
**Time point (days)**	**-21 to -1**	**1**	**14**	**28**	**42**	**56**	**84**	**126**	**168**						**Last**
**Visit no.**	**101**	**102**	**103**	**104**	**105**	**106**	**107**	**108**	**109**				**778 or 779**	**501**	**780[t]**

j. AEs will continue to be recorded on the Adverse Event CRF page up to 4 weeks (28 days) after the last dose of study drug (until the follow-up visit). All SAEs occurring within 28 days of study treatment discontinuation (until the follow-up visit), regardless of causality, should be captured on the Adverse Event CRF page. SAEs with suspected causality to study drug should be captured for an additional 8 weeks (56 days) after follow-up visit (for a total of 12 weeks (84 days) after treatment discontinuation).

k. Study drug will be dispensed at the indicated visits. At each dispensing visit, site personnel will log into IWRS to obtain the patient's study drug assignment to last until the next dispensing visit including sufficient overage.

l. At each study visit, site personnel will review the patient's returned study drug, foil packs (used and unused) to ensure the patient is compliant.

m. **For patients with skin lesions at screening/baseline of the blinded treatment phase**: Skin lesions will be photographed using a digital camera and measured using a disposable ruler. Photographs will be taken at baseline before the first dose of open-label RAD001, at 12 and 24 weeks after start of open label RAD001, and **every 12 weeks thereafter and end of treatment**. A confirmatory skin lesion response assessment should be performed approximately 12 weeks later (and no sooner than 8 weeks later).

n. Additional menstrual history (age at menarche, previous cases of amenorrhea or menstrual disorders, biological mother's age at menopause) and pregnancy history (Pregnancies, full-term gestations, abortions, live births, miscarriages) will be collected one time after implementation of protocol amendment 3. Following implementation of amendment 3, menstrual status will be collected monthly via patient diary for source documentation. Every 12 weeks, data from patient diary will be collected at clinical site for CRF. It is recommended that study coordinators contact patients monthly for the first 3 months to remind patients to document menstrual status in patient diary.

o. If an investigator suspects a patient may be developing pneumonitis, investigations such as pulmonary function tests, CT chest and referral to a pulmonologist should be considered. Chest CT scan should be performed as clinically indicated.

p. CRF for Study Evaluation Completion will only be completed at the end of all study-related activity, including any long-term follow-up assessments when the patient is not taking study drug

Table 7-3 Non-interventional follow-up phase visit evaluation schedule

Assessment	End of extension phase	Entry into Non-interventional follow-up phase	Non-interventional follow-up phase	Exit from non-interventional follow-up phase/Study Evaluation Completion
**Time point (duration)**			**≤ 1-Year**	
**Visit number**	**779[c]**	**201**	**551**	**780[d]**
Written Informed Consent (S)	X	X		
Inclusion/exclusion criteria (D)		X		
CT/MRI (D)	X[a]		X[b]	
Adverse events[e] (including angiomyolipoma-related bleeding) (D)			as reported	
Concomitant medications[e] (D)			as reported	

a. For all patients participating in this non-interventional follow-up phase, kidney CT/MRI should be performed at the beginning of this period, if not done within the past 2 months. For each patient same imaging modality must be used.

b. The kidney CT/MRI scan at the end of the non-interventional follow-up phase should be obtained prior to the initiation of an intervention or within 3 weeks of the 1-year anniversary of the last dose of study treatment, whichever comes first. Interventions include commercial everolimus, off label rapamycin, embolization or nephrectomy at any time during this 1-year period.

c. The EOT for the extension phase is 779.

d. CRF for Study Evaluation Completion will only be completed at the end of all study-related activity, including any long-term follow-up assessments when the patient is not taking study drug

e. Angiomyolipoma-related bleeding, diagnosis, and concomitant medications and non-drug therapies given in treatment of angiomyolipoma or angiomyolipoma-related disease progression should be captured on the CRF. Other adverse events and other concomitant medications will only be captured as reported by the patient for the DS&E database.

7.1 Information to be collected on screening failures

Patients who complete the informed consent process and do **not** meet all entry criteria and therefore do not receive RAD001 or matching placebo will be considered screen failures. Screen failures should be entered into the Screen Failure Log. The screening failure data will be entered in the clinical database.

7.2 Inclusion/exclusion criteria

Information regarding eligibility criteria will be collected on the Inclusion/Exclusion CRF. Patients who do not meet all entry criteria should not be entered into the study.

7.3 Patient demographics/other baseline assessments

Data will be collected on patient characteristics including demographic information (age, sex, race, weight) and other background or relevant medical history (disease history, family history of disease, prior anti-angiomyolipoma therapies), hepatitis and HIV history and any other assessments that are done for the purpose of determining eligibility for inclusion in the study (i.e., WHO Performance Status, complete physical examination, vital signs, hematology, blood chemistries including coagulation studies and a serum lipid profile, urinalysis, pregnancy test (only required for women of child-bearing potential),CT/MRI, ECG, chest X-ray). Medical history will include family history of disease. Serum pregnancy test is required at screening/baseline to be followed by a urine pregnancy test on Treatment day 1 (prior to dosing) to confirm eligibility. Urine pregnancy testing will be repeated every 4 weeks while receiving study drug until study drug discontinuation, and a serum pregnancy test will be repeated at either the end of treatment or the end of the study (depending upon the reason why the patient discontinued study medication).

Baseline assessments of target lesions

A CT/MRI assessment of the kidneys will be performed for all patients at baseline to identify any angiomyolipoma with longest diameter ≥ 1.0 cm and any SEGAs with longest diameter ≥ 1.0 cm, respectively. For information regarding the scan acquisition protocol, please refer to the Independent Radiology Review Charter. These images will be sent to the independent review committee within 2 days of the scan for estimation of tumor volume at each time point.

7.3.1 Special laboratory tests

7.3.1.1 Pregnancy test

Women of child-bearing potential, defined as all women physiologically capable of becoming pregnant, must use highly effective contraception during the study and for 8 weeks after stopping treatment. Highly effective contraception is defined as either:

- Total abstinence: When this is in line with the preferred and usual lifestyle of the subject. (Periodic abstinence (e.g., calendar, ovulation, symptothermal, post-ovulation methods) and withdrawal are not acceptable methods of contraception)

- Sterilization: have had surgical bilateral oophorectomy (with or without hysterectomy) or tubal ligation at least six weeks before taking study treatment. In case of oophorectomy alone, only when the reproductive status of the woman has been confirmed by follow up hormone level assessment
- Male partner sterilization (with the appropriate post-vasectomy documentation of the absence of sperm in the ejaculate). (For female subjects on the study, the vasectomised male partner should be the sole partner for that subject)
- Use of a combination of any two of the following (a+b or a+c or b+c):
  a. Use of oral, injected, implanted or other hormonal methods of contraception
  b. Placement of an intrauterine device (IUD) or intrauterine system (IUS)
  c. Barrier methods of contraception: Condom or Occlusive cap (diaphragm or cervical/vault caps) with spermicidal foam/gel/film/cream/vaginal suppository
- In case of use of oral contraception women should have been stabile on the oral agent before taking study treatment.

Sexually active males must use a condom during intercourse while taking the drug and for 8 weeks after stopping treatment and should not father a child in this period.

A condom is required to be used also by vasectomised men in order to prevent delivery of the drug via seminal fluid.

Female partners of male patients must also be advised to use one of the following contraception methods: Use of (1) oral, injected, implanted or other hormonal methods of contraception, or (2) intrauterine device (IUD) or intrauterine system (IUS), or (3) prior male/female sterilization.

A serum pregnancy test must be completed within 14 days of randomization and a urine pregnancy test follow-up completed prior to randomization on Treatment Day 1. Female patients who are able to become pregnant will have a repeat urine pregnancy test every 4 weeks while receiving study drug until study drug discontinuation. Pregnancy testing is required at screening and monthly until the end of the study (core and extension). Serum pregnancy testing should be performed at screening and at either the end of treatment or the end of the study (depending upon the reason why the patient discontinued study medication). Urine pregnancy testing will be performed every 4 weeks at the patient’s home or at clinical site when a visit is scheduled. Urine pregnancy test will be conducted at clinical site in place of patient’s home when a visit is scheduled.

Patient should be instructed to inform site of a positive urine pregnancy result. Repeat serum pregnancy testing will be performed for confirmation of a positive urine pregnancy test.

7.3.1.2 HBV testing

Prior to randomization, the categories of patients listed in Section 4.1 should be tested for hepatitis B serologic markers and viral load: HBV-DNA, HBsAg, HBcAb, and HBs Ab. If a patient tests positive, he/she will be considered ineligible for the study according to Exclusion Criterion 8. Please note that patients who test negative for HBV-DNA, HBsAg, and HBcAb but positive for HBs Ab, due to prior history of vaccination against Hepatitis B will be

eligible. The fact that the patient had been vaccinated should be entered into the patient's Medical History CRF.

For patients who have been randomized prior to the approval of Amendment 1, the above screening tests for hepatitis B should be completed at the patient's next study visit. Should the patient test positive, the investigator should follow the guidelines provided in Table 4-1 and Table 4-2.

7.3.1.3 HCV testing

Patients with hepatitis C risk factors and additional patients at the direction of the investigator should be tested for HCV RNA-PCR test at baseline. For a list of hepatitis C risk factors, refer to Section 4.1. Patient with a positive result must be excluded from the study.

For patients who have already been randomized and received study drug prior to the approval of Amendment 1, the same screening process should be followed at the patient's next visit.

Please refer to Table 7-1 and Table 7-2 for HCV RNA-PCR monitoring schedule for those patients with positive HCV RNA-PCR baseline tests who do not meet the reactivation criteria outlined in Table 4-3. If the patient tests positive for hepatitis C and the criteria for reactivation according to Table 4-3 are observed, trial therapy should be discontinued and further treatment is up to the investigators discretion.

7.3.1.4 Endocrine testing

A blood sample for analysis of total testosterone, FSH and LH (all patients) and estradiol (female patients) will be obtained at the screening/baseline visit and every 12 weeks after the start of study drug. In the event that amenorrhea is reported in between scheduled assessments, hormone evaluations should be completed at that time.

For patients who have already been randomized and did not have the endocrine parameters tested, there will be no retrospective analysis of blood samples.

7.4 Treatments

Patients will start study treatment at Visit 2 (Treatment Day 1) and continue to be treated per protocol until documentation of angiomyolipoma progression, unacceptable toxicity, withdrawal of consent or investigator decision to stop the study. However, study treatment may prematurely be discontinued for other reasons as well; please refer to Section 6.7.5.

Compliance will be assessed by the investigator and/or study personnel at each visit using pill counts and information provided by the caregiver. This information should be captured in the source document at each visit.

7.5 Efficacy

7.5.1 Radiological evaluation

For instruction regarding baseline assessments of angiomyolipomata, please refer to Table 7-1 and Section 7.3.

The same method of assessment and the same technique should be used to characterize each identified and reported angiomyolipoma at baseline, at 12, 24 and 48 weeks, and annually thereafter.

Each center must have a designated radiologist or other physician who is responsible for the interpretation of CT scan or multiphase MRI. The same radiologist/physician should perform the evaluation for the entire duration of the study. All radiology evaluations will be performed initially by the local radiologist, but designation of response and progression will be based only on the evaluations made by the Independent Central Radiology Review. CT/MRIs must be digitized and sent to central radiology within 2 days of the scan date. Following receipt of each scan, the Independent Central Radiology Review will be completed and the results will be communicated back to the participating center within 3 weeks.

The presence or absence of polycystic kidney disease will be documented at baseline by the central reviewer.

If an initial observation of angiomyolipoma response is made, a confirmation scan should be obtained approximately 12 weeks after the initial observation (and no sooner than 8 weeks later).

All patients being discontinued from the study for angiomyolipoma progression must have their progression documented using the criteria specified in Section 7.5.2. In particular, a discontinuation reason of "angiomyolipoma progression" will not be sufficient to establish that angiomyolipoma progression actually occurred.

All patients who are participating in the non-interventional follow-up phase, a kidney CT/MRI scan should be obtained after 1-year of the last dose of study treatment or prior to the initiation of an intervention. Please refer to Table 7-3 and Section 4.6.

7.5.2 Angiomyolipoma response evaluation

Angiomyolipoma response and progression evaluation will be performed according to the criteria outlined below.

Screening/baseline requirement:

At baseline, all measurable angiomyolipomata with longest diameter ≥ 1.0 cm should be identified from each kidney. Only patients with at least one angiomyolipoma ≥ 3.0 cm in longest diameter will be eligible to be randomized into the trial. All baseline evaluations should be performed as close as possible to the beginning of treatment and never more than 21 days before the beginning of treatment. The same imaging modality must be used throughout the trial.

Target lesions:

Up to five of the largest measurable lesions on each kidney seen at baseline, where measurable means at least 1.0 cm in longest diameter, should be identified as target angiomyolipomata. The volume of these lesions will be measured at each kidney CT/MRI assessment during the trial. The same imaging modality must be used throughout the trial. Angiomyolipoma volume is defined as the sum of the volumes of the individual target

angiomyolipomata, and it is angiomyolipoma volume that is used directly in the definition of angiomyolipoma response and angiomyolipoma progression.

Kidney volume:

All other angiomyolipomata (i.e., lesions other than the target angiomyolipomata as defined above) present at baseline are non-target angiomyolipomata. In some cases there may be many non-target angiomyolipomata (e.g., >20), including non-measurable lesions (i.e., with longest diameter < 1.0 cm). Instead of attempting to individually assess each non-target angiomyolipoma during the trial, the volume of each kidney will be measured. Increases in the volume of either kidney will then be taken as evidence of worsening angiomyolipoma. This is expected to be particularly useful when target angiomyolipomata are relatively stable, but non-target angiomyolipomata are clearly progressing.

Angiomyolipoma response assessment:

Angiomyolipoma response will be defined as:

- a reduction in angiomyolipoma volume of at least 50% relative to baseline, where angiomyolipoma volume is the sum of the volumes of all target angiomyolipomata identified at baseline, and confirmed with a second scan performed approximately 12 weeks later (and no sooner than 8 weeks later).

In addition, angiomyolipoma response requires satisfying all of the following criteria:

- no new angiomyolipomata ≥ 1.0 cm in longest diameter are identified
- neither kidney increases in volume by more than 20% from nadir (where nadir is the lowest kidney volume obtained for the patient, separately for each kidney, previously in the trial including baseline)
- the patient does not have any angiomyolipoma-related bleeding of grade ≥2 (as defined by NCI CTCAE, version 3.0).

Angiomyolipoma progression will be defined as one or more of the following:

- an increase from nadir of 25% or more in angiomyolipoma volume to a value greater than baseline (where angiomyolipoma volume is the sum of the volumes of all target angiomyolipomata identified at baseline and where nadir is the lowest angiomyolipoma volume achieved by the patient previously in the trial (including baseline))
- the appearance of a new angiomyolipoma ≥ 1.0 cm in longest diameter
- an increase from nadir of 20% or more in the volume of either kidney to a value greater than baseline, where nadir is the lowest kidney volume obtained for the patient, separately for each kidney, previously in the trial (including baseline)
- angiomyolipoma-related bleeding grade ≥2 as defined by NCI CTCAE, version 3.0.

Note: In some instances, disease that is measurable as a target lesion at baseline and appears to be one mass can split to become two or more smaller sub-lesions. When this occurs, the image review system allows the central reviewer to identify the separate sub-lesions as unique and non-overlapping so that the volume of each sub-lesion can be determined. The combined volumes of the sub-lesions will be reported as the volume of the lesion that has split. The individual split lesions will not be considered as new lesions, and will not automatically trigger an angiomyolipoma progression.

Conversely, it is also possible that two or more lesions which were distinctly separate at baseline become confluent at subsequent visits. The central reviewer will be required to separate the confluent mass into regions relating to the original several distinct lesions. These regions will continue to be measured as separate lesions. Although this raises the possibility of increased variability in the measurements of the individual lesions due to the lack of distinct borders within the confluent mass, the primary endpoint (sum of lesion volumes) will still be accurately measured so long as the confluent mass itself has distinguishable borders.

7.5.3

7.5.4 Skin lesions

Skin lesions resulting from TSC include hypomelanotic macules, the shagreen patch, periungual or subungual fibromas, facial angiofibromas and/or forehead plaques. Descriptions of each are given below.

Hypomelanotic macules are flat areas of skin that appear lighter than the surrounding skin. They can be any size or shape or may be the classic "ash-leaf" shape. Skin cells in this areacontain less pigment, so the area appears lighter than the surrounding skin.

The shagreen patch is a patch of skin that is similar in color to surrounding skin, but may be tough and dimpled like an orange peel. The shagreen patch is usually found on the lower back and nape of the neck, but they may also be seen on other parts of the body.

Periungual or subungual fibromas are small fibrous growths that appear around the fingernails or toenails and are usually not seen until adult life.

Facial angiofibromas are benign tumors of the face that often appear across the cheeks and nose and on the chin. They are initially small reddish spots or bumps that may increase in size with age.

Lastly, a forehead plaque is similar to the angiofibroma but is found on the forehead and scalp. These flesh-colored plaques are soft or compressible of doughy to hard lesions.

Digital photographs of all skin lesions will be taken at screening/baseline and then every 12 weeks throughout the study and at End of treatment.

7.5.4.1 Skin lesion response evaluation

7.5.4.1.1 Physician's Global Assessment of Clinical Condition (PGA)

The Physician's Global Assessment of Clinical Condition (PGA) is a 7-point grading scale that allows the investigator to evaluate the overall extent of improvement or worsening of the patient's skin disease as compared to baseline (see Table 7-4). This scale has been previously used in assessing skin lesions in other phase III trials (Duvic 2001, Heald 2003). **Whenever possible, the same investigator should perform all skin evaluations on a patient in order to avoid inter-assessor variability.** This assessment is designed to consider skin lesions as a whole. Responses must be confirmed by at least two assessments separated in time by approximately 12 weeks (and no sooner than 8 weeks). A complete clinical response (CCR) requires a grading of 0 indicating the absence of disease (histological confirmation is not

required). Grades 1, 2, and 3 constitute partial response, indicating improvement of at least 50 percent, but less than 100 percent improvement.

Once a patient begins laser treatment or receives local surgery to treat their skin lesions, any data obtained from that point onward will be excluded from the skin lesion response analysis.

Table 7-4 Physician's Global Assessment of Clinical Condition (PGA)

Grade	Description	Response
0 Completely clear	No evidence of disease; 100% improvement	CCR
1 Almost clear	Very significant clearance (≥90%-<100%); only traces of disease remains	PR
2 Marked improvement	Significant improvement (≥75%-<90%); some evidence of disease remains	PR
3 Moderate improvement	Intermediate between slight and marked improvement; (≥50 %-< 75%)	PR
4 Slight improvement	Some improvement (≥25%-<50%);however, significant evidence of disease remains	SD
5 No change	Disease has not changed from baseline condition (±<25%)	SD
6 Worse	Disease is worse than at baseline evaluation by ≥25% or more	PD

CCR, Clinical complete response; PR, Partial response; SD, Stable disease; PD, Progressive disease.

7.5.4.2 Digital photographs

Digital photographs of all skin lesions should be taken at baseline, using a high resolution digital camera (≥3 megapixels), and a caliper or ruler should be included in the photo whenever possible. The photographs will be repeated every 12 weeks after the start of study treatment and at End of treatment. If a patient demonstrates a CCR or PR evaluated by PGA assessment, further photographs should be taken approximately 12 weeks later (and no sooner than 8 weeks later) to confirm the response. These digital photographs will be used solely to document the response of these skin lesions to study treatment and should be sent to the central review facility for archiving.

7.6 Safety

Safety assessments will consist of monitoring and recording all adverse events, including SAE's and the regular monitoring of hematology and blood chemistry, vital signs and physical condition.

These assessments should be performed within 7 days of the scheduled day of assessment (Table 7-1 and Table 7-2) except for AE's and concomitant medications that will be evaluated and recorded continuously throughout the study.

During the non-interventional follow-up phase, except for angiomyolipoma-related bleeding, adverse event data and concomitant medication data will only be collected, as reported by the patient for the DS&E database.

7.6.1 Adverse events

An adverse event for the purposes of this protocol is the appearance of (or worsening of any pre-existing) undesirable sign(s), symptom(s), or medical condition(s) occurring after signing the informed consent even if the event is not considered to be related to the study drug(s). Please refer to Section 6.1 for the protocol-specific definitions of study drug and study treatment.

Adverse events (but not serious adverse events) occurring before starting study treatment but after signing the informed consent form are recorded on the Relevant Medical History/Current Medical Conditions CRF. Abnormal laboratory values or test results constitute adverse events only if they induce clinical signs or symptoms, or are considered clinically significant or require therapy (e.g., any hematological abnormality that requires transfusion or cytokine treatment), and should be recorded on the Adverse Events CRF under the signs, symptoms or diagnosis associated with them. In addition, isolated abnormal laboratory values that are considered clinically significant (e.g., cause study drug discontinuation or constitutes in and of itself a Serious Adverse Event) should be recorded on the Adverse Events CRF. SAEs occurring after signing the Informed Consent and prior to starting study treatment are recorded on the Medical History CRF if the patient continues on with the study or the Adverse Events CRF if the patient withdraws from the study prior to starting study treatment.

Adverse events will be assessed according to the NCI Common Terminology Criteria for Adverse Events (CTCAE) version 3.0. If CTCAE grading does not exist for an adverse event, the severity of mild, moderate, severe, and life-threatening, corresponding to grades 1 - 4, will be used. CTCAE grade 5 (death) will not be used in this study; rather, this information will be collected on the End of Treatment or Study Evaluation Completion CRF pages. Adverse event monitoring should be continued for at least 28 days following the last dose of study treatment.

The occurrence of adverse events should be sought by non-directive questioning of the patient at each visit during the study. Adverse events also may be detected when they are volunteered by the patient during or between visits or through physical examination, laboratory test, or other assessments. As far as possible, each adverse event should be evaluated to determine:

1. The severity grade, CTCAE grade 1-4
2. Its relationship to study drug (suspected/not suspected)
3. Its duration (start and end dates or if continuing at final exam)
4. Action taken (no action taken; study drug dosage adjusted/temporarily interrupted; study drug permanently discontinued due to this adverse event; concomitant medication taken; non-drug therapy given; hospitalization/prolonged hospitalization)
5. Whether it is serious, where a serious adverse event (SAE) is defined as one which:
   - Is fatal or life-threatening
   - Results in persistent or significant disability/incapacity
   - Constitutes a congenital anomaly/birth defect
   - Requires inpatient hospitalization or prolongation of existing hospitalization, unless hospitalization is for:
     - Routine treatment or monitoring of the studied indication, not associated with any deterioration in condition
     - Elective or pre-planned treatment for a pre-existing condition that is unrelated to the indication under study and has not worsened since signing the informed consent
     - Treatment on an emergency outpatient basis for an event not fulfilling any of the definitions of an SAE given above and not resulting in hospital admission
     - Social reasons and respite care in the absence of any deterioration in the patient's general condition

- Is medically significant, i.e., defined as an event that jeopardizes the patient or may require medical or surgical intervention to prevent one of the outcomes listed above.

Unlike routine safety assessments, SAEs are monitored continuously and have special reporting requirements; see Section 8.1.

All adverse events should be treated appropriately. Such treatment may include changes in study drug treatment including possible interruption or discontinuation, starting or stopping concomitant treatments, changes in the frequency or nature of assessments, hospitalization, or any other medically required intervention. Once an adverse event is detected, it should be followed until its resolution, and an assessment should be made at each visit (or more frequently, if necessary) of any changes in its severity, its suspected relationship to the study drug(s), any of the interventions required to treat it, and its outcome.

Information about common side effects already known about the investigational drug can be found in the most recent version of the [Investigator's Brochure] (IB) and in addition will be communicated between IB updates in the form of Investigator Notifications. This information will be included in the patient informed consent and should be discussed with the patient during the study as needed.

During the non-interventional follow-up phase, all patients must report any angiomyolipoma-related bleeding events. Diagnostic procedure(s) pertaining to angiomyolipoma-related bleeding should also be reported. All other adverse events occuring within the one-year period will only be collected, as reported by the patient for the DS&E database.

7.6.2 Physical examination

Physical examination must include a total body examination (general appearance, skin, neck, including thyroid, eyes, ears, nose, throat, lungs, heart, abdomen, back, lymph nodes, extremities and a basic nervous system neurological exam). Significant findings must be recorded either as Relevant Medical History / Current Medical Conditions (if present before treatment) or as Adverse Events (if newly occurring or worsening since starting treatment).

7.6.3 Vital signs

Pulse, respiratory rate, blood pressure and temperature, and height (screening visit only) and weight will be measured as indicated in the evaluation schedule (Table 7-1 and Table 7-2) and will be recorded on source documents and entered on the Vital Signs CRF pages. Blood pressure, pulse and respiratory rate should be measured on patients after at least 3 minutes in the sitting position.

7.6.4 WHO performance status and scale

WHO performance status will be measured as indicated in the assessment schedules (Table 7-1 and Table 7-2).

Performance Status WHO grade:

Grade 0: Fully active, able to carry out all normal activity without restriction.

Grade 1: Restricted in physically strenuous activity but ambulatory and able to carry out work of a light or sedentary nature, e.g., light house work, office work.

Grade 2: Ambulatory and capable of all self-care but unable to carry out any work activities. Up and about more than 50% of waking hours.

Grade 3: Capable of only limited selfcare, confined to bed or chair more than 50% of waking hours.

Grade 4: Completely disabled. Cannot carry on any self-care. Totally confined to bed or chair.

Grade 5: Dead

7.6.5 Laboratory evaluations

All standard clinical laboratory analyses described below are to be performed by the central laboratory, according to the Visit Schedule outlined in Table 7-1. The name of the central laboratory can be found in the investigator binder supplied to the site. Details about all central laboratory procedures including collection, shipment of samples, reporting of results and alerting of extreme values are given in the manual provided by the central laboratory. Notable values will be provided in the [Laboratory Manual].

For clinically relevant laboratory values from local labs, the actual laboratory value should be recorded on the Local Lab CRFs and local lab ranges should be provided on the Lab Normal Range sheet as appropriate. Unscheduled abnormal laboratory evaluations which are clinically relevant (e.g., require dose modification and/or interruption of study drug, indicate changes in previously abnormal values) must be recorded on the Adverse Events CRF.

Screening safety laboratory tests (hematology, biochemistry, lipid profile, urinalysis and coagulation) will need to be repeated at Treatment Day 1 if they were collected more than 14 days prior to Treatment Day 1. For the week 6 visit (visit 5 in the blinded portion and visit 105 in the open label/extension portion), patients residing in the United States for whom travel is difficult, who have tolerated study medication and have no adverse events may, at the discretion of the investigator, attend a local laboratory affiliated with EXAM **One** for blood draws (if such facility is available) and have a telephone consultation with the investigator rather than a clinic visit (detailed instructions are provided in the [Quest Diagnostic Laboratory Manuals]).

Patients will be advised to fast on the days of scheduled visits prior to blood sampling period (note: patients are allowed to consume water during this time). A light breakfast may be taken after this point, prior to trial therapy administration, however, patients should be advised to avoid fatty meals.

7.6.5.1 Hepatitis screen

Patients who have a positive test for Hepatitis B or C at screening must be excluded from the study. Please note that patients who test negative for HBV-DNA, HBsAg, and HBcAb but positive for HBs Ab, due to prior history of vaccination against Hepatitis B will be eligible. The fact that the patient had been vaccinated should be entered into the patient's Medical History CRF.Hepatitis testing will be done only at screening (see Table 7-1) for patients that

meet the criteria outlined in Section 4.1. Guidelines for those patients that have already been randomized prior to the approval of Amendment 1.5.1 are detailed in Table 4-1 and Table 4-2.

If an already randomized patient (at the time of Amendment 1 approval) tests positive for Hepatitis C, and the criteria for reactivation according to Table 4-3 are observed, the patient should discontinue study treatment. Please refer to Table 7-1 and Table 7-2 for HCV RNA-PCR monitoring schedule for those patients with positive HCV RNA-PCR, baseline tests who have not yet met the reactivation criteria outlined in Table 4-3.

7.6.5.2 Hematology

Hematology tests will be performed at each scheduled visit as indicated in Table 7-1 and Table 7-2. The hematology profile must include: hemoglobin, hematocrit, platelets, red blood cell count (RBC), total white blood cell count (WBC) absolute & differential (including neutrophils, lymphocytes, monocytes, eosinophils, basophils). Absolute neutrophil count (ANC) will be calculated by the central laboratory.

7.6.5.3 Coagulation

Prothrombin time (PT) will be determined at screening and every 12 weeks after the start of study treatment; it will be reported as international normalized ratio (INR). In addition, fibrinogen and partial thromboplastin time (PTT) will be determined at screening and every 12 weeks after the start of study treatment. The amount of blood to be drawn for these assessments can be found in the central [Laboratory Manual].

7.6.5.4 Biochemistry and lipid profile

Biochemistry tests including serum lipid profile will be performed at each scheduled visit as indicated in Table 7-1 and Table 7-2: sodium, potassium, chloride, bicarbonate, creatinine, albumin, total protein, SGOT (AST), SGPT (ALT), total bilirubin, alkaline phosphatase, uric acid, BUN, calcium, magnesium, phosphate, total LDH, and fasting glucose. Please refer to the central [Laboratory Manual] for the amount of blood to be drawn for these assessments at each visit.

In order to assess renal function during the trial, the Cockcroft-Gault formula will be used. The Cockcroft-Gault formula is shown below, where creatinine clearance is “*x*” (in mL/min), age is measured in years, weight in kg, creatinine in µmol/L, and the constant is 1.23 for men and 1.04 for women (Cockcroft et al 1976):

$$x = \frac{(140 - age) \times weight \times constant}{creatinine}$$

A lipid profile (cholesterol, triglycerides, LDL, HDL) will be determined at screening and repeated every 12 weeks while receiving study drug (at 12 and 24 weeks and every 12 weeks thereafter). The amount of blood to be drawn for this assessment is listed in. The patient must be in a fasting state at the time of blood sampling for this evaluation.

7.6.5.5 Urinalysis

During screening, a standard urinalysis (pH, protein, glucose, blood, ketones, and leukocytes) should be performed and submitted to the central laboratory. Urine dipstick will be performed

routinely thereafter at each study visit (excluding visits 2, 3 and 5). This must be supplemented with central laboratory quantification of any potentially relevant abnormalities.

7.6.5.6 Pregnancy test

All females of child-bearing potential must have a negative serum pregnancy test at screening/baseline as well as a negative urine pregnancy test (performed locally) prior to treatment on Treatment Day 1. Urine pregnancy tests will then be repeated every 4 weeks until discontinuation of study drug. A serum pregnancy test will be performed at either the end of treatment or end of study visit (depending upon the reason why the patient has discontinued study medication). Patient should be instructed to inform site of a positive urine pregnancy result. Repeat serum pregnancy testing will be performed for confirmation of a positive urine pregnancy test. It is recommended that postmenopausal women be amenorrheic for at least 12 months or have a serum follicle-stimulating hormone (FSH) of >40 mIU/ml to be considered “of non-childbearing potential” or 6 weeks post surgical bilateral oophorectomy with or without hysterectomy.

Highly effective contraception must be used on-study and for up to 8 weeks after ending treatment (definition of highly effective contraception is detailed in Section 7.3.1.1).

7.6.5.7 Reproductive history and endocrine testing

In order to provide additional follow-up on cases of amenorrhea, increased hormonal evaluation will be performed and supplemental medical history will be collected.

Additional menstrual history (age at menarche, previous cases of amenorrhea or menstrual disorders, biological mother’s age at menopause) and pregnancy history (pregnancies, full-term gestations, abortions, live births, miscarriages) will be collected one time after implementation of protocol amendment 3. Following implementation of amendment 3, menstrual status will be collected monthly via patient diary. Every 12 weeks, data from patient diary will be collected at clinical site for CRF. It is recommended that study coordinators contact patients monthly for the first 3 months to remind patients to document mentrual status in patient diary.

A blood sample for analysis of total testosterone, FSH and LH (all patients) and estradiol (female patients) will be obtained at the screening/baseline visit and every 12 weeks after the start of study drug. In the event that amenorrhea is reported in between scheduled assessments, hormone evaluations should be completed at that time.

For patients who did not have the endocrine parameters tested, there will be no retrospective analysis of blood samples.

7.6.6

7.6.7 Electrocardiogram (ECG)

A standard 12-lead ECG is to be performed at screening/baseline and at end of treatment. Tracings must be dated and signed by the investigator (or his/her designee) and filed with the subject’s source documentation. Results from 12-lead ECG should be captured on the ECG Evaluation CRF. Significant findings must be recorded as Relevant Medical History / Current

Medical Conditions (if present before treatment). ECG may be repeated at the discretion of the investigator at any time during the study and as clinically indicated; any clinically relevant findings should be recorded on the Adverse Events CRF.

7.7 Pharmacokinetics

Biofluid concentrations will be expressed in mass per volume units. All concentrations below the limit of quantification or missing data will be labeled as such in the concentration data listings. Concentrations below the limit of quantification will be treated as zero in summary statistics.

Patients will be advised to fast overnight on the days of blood sampling for RAD001 prior to the sampling period (note: patients are allowed to consume water during this time). A meal may be taken afterthe pre-dose sample has been collected, but prior to ingestion of the daily RAD001 dose; patients should be advised to avoid fatty meals.

7.7.1 Pharmacokinetics blood sample collection and handling

During the blinded treatment phase of the study, pre-dose trough blood samples for determination of RAD001 concentration (C_{min}) will be collected immediately prior to dosing at weeks 2, 4, 12, 24 and 48. **A trough blood sample is defined as a sample of blood collected 22-26 hours for daily dosing (or 46-50 hours for every other day dosing) after the patient's last dose of study drug, following 5 days of consistent (daily dose and timing of dose) dosing.** In addition to trough blood samples, a PK blood sample (2 mL) will be taken 2 h (± 30 mins, C_{2h}) after trial therapy dose administration at weeks 2, 4, 12, 24 and 48. Pre-dose trough (C_{min}) and 2 h (± 30 mins, C_{2h}) blood samples will be collected in both treatment arms by either direct venipuncture or an inserted indwelling cannula. Please refer to Table 7-5 for amount of blood to be drawn for each trough (C_{min}) and 2 h (± 30 mins, C_{2h}) PK assessment and the PK sample number that should be assigned to each sample.

For the 6 week visit (visit 5 in the blinded portion and visit 105 in the open label/extension portion), patients residing in the United States for whom travel is difficult, who have tolerated study medication and have no adverse events may, at the discretion of the investigator, attend a local laboratory affiliated with EXAM One for blood draws (if such facility is available) and have a telephone consultation with the investigatory rather than a clinic visit (detailed instructions are provided in the [Quest Diagnostics Laboratory Manual]).

Table 7-5 PK sample log table

Study Day	Study Week	Time	PK Collection Number	PK Sample Number	Sample Volume (mL)
Day 14	Week 2	Pre-dose	101	101	2 mL
Day 14	Week 2	2 h (± 30 min)	1	201	2 mL
Day 28	Week 4	Pre-dose	102	102	2 mL
Day 28	Week 4	2 h (± 30 min)	2	202	2 mL
Day 84	Week 12	Pre-dose	103	103	2 mL
Day 84	Week 12	2 h (± 30 min)	3	203	2 mL
Day 168	Week 24	Pre-dose	104	104	2 mL
Day 168	Week 24	2 h (± 30 min)	4	204	2 mL
Day 336	Week 48	Pre-dose	105	105	2 mL

Day 336	Week 48	2 h (± 30 min)	5	205	2 mL

Blood sample collection

A 2 mL sample of venous blood will be drawn for RAD001 blood concentration determination from a forearm vein into tubes containing EDTA. The tube will be inverted several times to mix contents (e.g., anti-coagulant) immediately after collection of the blood sample. Prolonged contact must be avoided with rubber stopper. The whole blood sample will be transferred to a labeled polypropylene screw cap tube and frozen at - 20°C or below within 60 minutes of venipuncture. An example of the label that should be attached to the tube is shown below. The respective PK sample number shown in Table 7-5 should be entered on each label next to "sample number".

PK labels will be designed as follows:

Study number	CRAD001M2302
Subject initials and number	_ _ _ / _ _ _ _ - _ _ _ _ _
Analyte	Everolimus
Sample number	_ _ _

Sample collection handling

The actual collection time of all samples must be documented on the PK Blood Collection CRF pages. **The date and actual time of the last dose of study drug and date and time of the dose of study drug taken on the day of the sampling must be recorded on the PK Blood Collection CRF. In addition, dates and times of blood samplings must be entered on the PK Blood Collection CRF.** Any sampling problems (i.e., patient took study drug before a trough [pre-dose]) must be noted in the comments section of the CRF.

In order to assure compliance with sampling procedures, **on days of drug level and PK assessment, drug administration should be supervised by study center personnel**.

If the patient's dose has been interrupted for more than 48 hours prior to when a PK blood collection is required, it is not necessary to collect the PK sample. If the patient vomits within the first 4 hours following study-drug administration on the day of PK blood sampling, the time (using the 24-hour clock) of vomiting should be recorded on the PK Blood Collection CRFs. No additional study drug should be taken that day to replace the dose that was vomited.

7.7.2 Pharmacokinetic sample shipment

All blood samples must be carefully packed in suitable packing material containing sufficient dry ice to keep them frozen during shipment.

A list of all samples, including the date, subject number, and time of sampling should be sent with the shipment. Any missing samples should be noted on the list.

Samples will be sent to Quest Diagnostics Clinical Trials Labs 27027 Tourney Road, Ste. 2E; Valencia, CA 91355. Shipments should all be sent on Monday, Tuesday, or Wednesday, using a carrier guaranteeing overnight delivery (e.g., World Courier).

Please refer to the [Laboratory Manual] for specific sample shipment information.

7.7.3

7.8 Biomarkers

Biomarker studies are proposed using patient's plasma samples. These studies will focus on measuring the effect of RAD001 on soluble markers of angiogenesis. mTOR inhibitors have been shown to have an inhibitory effect on tumor growth and angiogenesis both *in vitro* and *in vivo*. VEGF and its family members are essential mediators of tumor angiogenesis. We plan to examine the effects of RAD001 on tumor vascularization through the measurement of these angiogenic growth factors and their corresponding soluble receptors. Data from these studies will be used to formulate hypotheses for future studies of RAD001 as an anti-angiogenic agent.

Patients will be asked to donate 3 mL of blood for plasma analysis at the following time points (also refer to Table 7-1) unless local or national regulations do not permit:

- screening/baseline
- 4 weeks
- 12 weeks
- 24 weeks
- 36 weeks
- 48 weeks
- End of Treatment

The blood samples for biomarker assessments should be collected immediately prior to study drug administration. On-treatment samples will be compared to baseline samples for RAD001 effects on plasma angiogenic molecules, e.g., basic FGF, VEGF, PLGF, soluble VEGF receptor1 and soluble VEGF receptor2. Exploratory assessments

7.8.1

8 Safety monitoring

8.1 Serious adverse event reporting

To ensure patient safety, every SAE, **regardless of suspected causality**, occurring after the patient has provided informed consent and until 4 weeks after the patient has stopped study treatment/participation must be reported to Novartis within 24 hours of learning of its occurrence. Any SAEs experienced after this 4-week period **(for up to 12 weeks (84 days))** should only be reported to Novartis if the investigator suspects a causal relationship to the study drug. Recurrent episodes, complications, or progression of the initial SAE must be reported as follow-up to the original episode within 24 hours of the investigator receiving the follow-up information. An SAE occurring at a different time interval or otherwise considered completely unrelated to a previously reported one should be reported separately as a new event.

Information about all SAEs is collected and recorded on the Serious Adverse Event Report Form. The investigator must assess and record the relationship of each SAE to each specific

study drug (if there is more than one study drug), complete the SAE Report Form in English, and send the completed and signed form by fax within 24 hours to the local Novartis Integrated Medical Safety.

The telephone and fax number of the contact persons in the local department of Pharmacovigilence Operations, specific to the site, are listed in the investigator folder provided to each site. The original copy of the SAE Report Form and the fax confirmation sheet must be kept with the CRF documentation at the study site.

Follow-up information is sent to the same person to whom the original SAE Report Form was sent, using a new SAE Report Form stating that this is a follow-up to the previously reported SAE and giving the date of the original report. Each recurrence, complication, or progression of the original event should be reported as a follow-up to that event regardless of when it occurs. The follow-up information should describe whether the event has resolved or continues, if and how it was treated, whether the blind was broken or not, and whether the patient continued or withdrew from study participation.

If the SAE is not previously documented in the current version of the [Investigator's Brochure], or Package Insert (new occurrence) and is thought to be related to the Novartis study drug, a Novartis Pharmacovigilance Operations or Integrated Medical Safety associate may urgently require further information from the investigator for Health Authority reporting. Novartis may need to issue an Investigator Notification (IN), to inform all investigators involved in any study with the same drug that this SAE has been reported. Suspected Unexpected Serious Adverse Events will be collected and reported to the competent authorities and relevant ethics committees in accordance with Directive 2001/20/EC or as per national regulatory requirements in participating countries.

8.2 Pregnancies

To ensure patient safety, each pregnancy in a patient on study drug must be reported to Novartis within 24 hours of learning of its occurrence. The pregnancy should be followed up to determine outcome, including spontaneous or voluntary termination, details of the birth, and the presence or absence of any birth defects, congenital abnormalities, or maternal and/or newborn complications.

Pregnancy should be recorded on a Clinical Trial Pregnancy Form and reported by the investigator to the local Novartis Integrated Medical Safety Department. Pregnancy follow-up should be recorded on the same form and should include an assessment of the possible relationship to the Novartis study drug of any pregnancy outcome. Any SAE experienced during pregnancy must be reported on the SAE Report Form.

Pregnancy outcomes must be collected for the female partners of any males who took study drug in this study. Consent to report information regarding these pregnancy outcomes should be obtained from the mother.

Preclinical data regarding reproductive toxicity is described in the most recent [Investigator Brochure]. The potential reproductive risk for humans is unknown.

Women of childbearing potential should be advised to use highly effective contraception methods while they are receiving everolimus and up to 8 weeks after treatment has been stopped.

If a pregnancy occurs while on study treatment, the newborn will be followed for at least 12 months.

8.3 Data Monitoring Committee

A Data Monitoring Committee (DMC) will be established prior to the randomization of the first patient; the same DMC will be used in any other Novartis-sponsored trials evaluating RAD001 in TSC-related diseases. The DMC is an external independent group including at least two physicians with expertise in TSC and one statistician. The DMC will perform the first safety review approximately 6 months after randomization of the first patient in any of the Novartis-sponsored RAD001 trials in TSC, and every 6 months thereafter, unless otherwise requested by the Chairman of the DMC. The DMC will also receive reports on a regular basis on all SAEs reported for this trial. No interim analysis is planned. Recruitment will not be interrupted unless otherwise requested by the Chairman of the DMC.

The responsibilities of the DMC include:

- Minimizing the exposure of patients to an unsafe therapy or dose
- making recommendations for changes in study processes where appropriate
- advising on the need for dose adjustments because of safety issues
- endorsing continuation of the study.

It will be the responsibility of the DMC to review the results of safety data 6 months after randomization of the first patient and every 6 months thereafter and to make recommendations to continue, modify or stop the study based on these results. The DMC will also receive detailed safety reports at regular intervals.

Details on the membership, responsibilities and working procedures of the DMC are described in the Data Monitoring Committee charter.

8.4 Steering Committee

The general role of the steering committee is to provide guidance on study conduct, to help ensure delivery of study data and to develop study-related publications in accordance with the Novartis publication and authorship policy. The steering committee will support the Novartis clinical team on a continuous basis when questions arise in the trial. The steering committee will monitor and supervise the progress of the trial towards its objectives. The committee will be appointed by Novartis and will include two principal investigators from this trial, Novartis staff and possibly other clinical experts. The committee will be chaired by one of the two principal investigators.

The steering committee chair will play a specific role in controlling the flow of trial information, in being the primary contact to receive recommendations from the DMC at any safety review and in further communicating this information appropriately, as described in the DMC charter.

9 Data review and data management

9.1 Site monitoring

Before study initiation, at a site initiation visit or at an investigator's meeting, a Novartis representative will review the protocol and CRFs with the investigators and their staff. During the study, the field monitor will visit the site regularly to check the completeness of patient records, the accuracy of entries on the CRFs, the adherence to the protocol and to Good Clinical Practice, the progress of enrollment, and to ensure that study drug is being stored, dispensed, and accounted for according to specifications. Key study personnel must be available to assist the field monitor during these visits.

The investigator must maintain source documents for each patient in the study, consisting of case and visit notes (hospital or clinic medical records) containing demographic and medical information, laboratory data, electrocardiograms, and the results of any other tests or assessments. All information on CRFs must be traceable to these source documents in the patient's file. The investigator must also keep the original informed consent form signed by patient (a copy is given to the patient).

The investigator must give the monitor access to all relevant source documents to confirm their consistency with the CRF entries. Novartis monitoring standards require full verification of the presence of informed consent, adherence to the inclusion/exclusion criteria, documentation of SAEs, and the recording of data that will be used for all primary efficacy and safety analyses. Additional checks of the consistency of the source data with the CRFs are performed according to the study-specific monitoring plan. No information in source documents about the identity of the patients will be disclosed.

9.2 Data collection

Designated investigator staff must enter the information required by the protocol onto the Novartis CRFs that are printed on 3-part, non-carbon-required paper. The PK summary pages of the CRF will also include an additional non-carbon required page, which will be sent to the bioanalytics pharmacokinetics group by the field monitor. All other summary pages will also include an additional non-carbon page that will be sent to Novartis by the field monitor. Field monitors will review the CRFs for completeness and accuracy and instruct site personnel to make any required corrections or additions. The CRFs are forwarded to the Contract Research Organization (CRO) by field monitors or by the investigational site, with one copy being retained at the investigational site. Once the CRFs are received by the CRO, their receipt is recorded, the original copy is placed in Central Files, and the non-carbon-required copy is forwarded to the responsible data management staff for processing.

The investigator must certify that the data are complete and accurate by signing a memo that will be sent to him/her by the CRO after the last transfer of the data prior to analysis. After database lock, the investigator will receive a memo detailing the obvious corrections that were made to the data.

Blood samples for laboratory data and biomarkers will be collected by sites and sent to a central laboratory for processing. The laboratory results will be sent electronically to the designated CRO.

Results from at home pregnancy test will be recorded in patient diaries for source documentation only.

Radiological imaging will be transmitted by the sites to the CRO designated by Novartis to undergo central radiological review.

Following implementation of amendment 3, menstrual status will be collected monthly via patient diary for source documentation. Every 12 weeks, data from patient diary will be collected at clinical site for CRF. It is recommended that study coordinators contact patients monthly for the first 3 months to remind patients to document menstrual status in patient diary.

9.3 Database management and quality control

The designated CRO staff will review the CRFs entered by investigational staff for completeness and accuracy and instruct the site personnel to make any required corrections or additions. Obvious errors will be corrected by the designated CRO personnel. Queries will be sent (faxed) to the investigational site using a paper Data Query Form. Designated investigator site staff should respond to the query and make any necessary changes to the data. Site personnel will complete and sign the faxed copy and fax it back to the CRO staff who will make the correction to the database. The signed copy of the Data Query Form is kept at the investigator site.

Concomitant medications entered into the database will be coded using the WHO Drug Reference List, which employs the Anatomical Therapeutic Chemical (ATC) classification system. Medical history/current medical conditions and adverse events will be coded using the Medical Dictionary for Regulatory Activities (MedDRA) terminology.

Laboratory samples will be processed centrally and the results will be sent electronically to the designated CRO.

Randomization codes and data about all study drug dispensed to the patient will be tracked using IWRS. The system will be supplied by a vendor, who will also manage the database. The data will be sent electronically to the designated CRO.

PK blood samples collected by sites will be shipped to Novartis for processing. The PK blood collection times will be entered by sites onto the CRFs. The PK blood sample results will be merged with the CRF PK blood collection times and analyzed by Novartis in accordance with internal Novartis procedures.

Blood samples for biomarkers will be collected and sent to a central laboratory for processing. The results will be sent electronically to the designated CRO.

At the conclusion of the study, the occurrence of any emergency code breaks will be determined after return of all code break reports and unused drug supplies to Novartis. The occurrence of any protocol deviations will be determined. After these actions have been completed and the database has been declared to be complete and accurate, it will be locked and the treatment codes will be unblinded and made available for data analysis. Any changes to the database after that time can only be made by joint written agreement between the Global Head of Biostatistics and Statistical Reporting and the Global Therapeutic Area Head.

10 Statistical methods and data analysis

The data will be analyzed by Novartis. Any data analysis carried out independently by the investigator should be submitted to Novartis before publication or presentation.

It is planned that the data from all centers that participate in this study will be used so that an adequate number of patients will be available for analysis.

10.1 Populations for analysis

The **Full Analysis Set (FAS)** consists of all randomized patients. Following the intent-to-treat principle, patients are analyzed according to the treatment and stratum that they were assigned to at randomization. The FAS will be the primary population in the assessment of efficacy.

The **Per Protocol Set (PPS)** will consist of all patients from the FAS without any major protocol deviation, who are evaluable for efficacy and who have completed a minimum exposure requirement. However, if a patient progressed, discontinued for adverse event or died before the minimum exposure requirement could be met, or before he/she was evaluated for efficacy, that patient will still be included in the PPS. Patients will be evaluable for efficacy if they have a known angiomyolipoma response status. The minimum exposure requirement is defined as having a relative dose intensity over the first 12 weeks of treatment of at least 50%. The PPS will be used for a supportive analysis of the primary endpoint.

The **Safety Population** will consist of all patients who received at least one dose of study treatment and had at least one post-baseline safety assessment (where the statement that a patient had no adverse event (on the Adverse Events CRF) constitutes a safety assessment). Patients will be analyzed according to treatment received.

10.2 Patient demographics/other baseline characteristics

Demographic and other baseline characteristics will be listed and summarized by treatment group using the FAS.

Qualitative data (e.g., gender, race, WHO performance status) will be summarized by means of contingency tables for each treatment group, and quantitative data (e.g., age, body weight) will be summarized by appropriate descriptive statistics (mean, standard deviation, median, minimum and maximum) for each treatment group.

10.3 Treatments (study drug, concomitant therapies, compliance)

10.3.1 Study medication

Duration of study treatment exposure, cumulative dose, dose intensity and relative dose intensity will be summarized by treatment group. The number of patients with dose changes/interruptions will be presented by treatment group, along with reasons for the dose change.

10.3.2 Concomitant therapies

Concomitant medications and significant non-drug therapies taken concurrently with the study drugs will be listed and summarized by ATC class, preferred term and treatment arm by

means of frequency counts and percentages. These summaries will include medications starting on or after the start of study treatment or medications starting prior to the start of study treatment and continuing after the start of study treatment.

Any prior concomitant medications or significant non-drug therapies starting and ending prior to the start of study treatment will be listed.

The safety population will be used for all above mentioned concomitant medication tables and listings.

10.4 Primary objective

The primary objective is to compare the angiomyolipoma response rate on RAD001 versus placebo in patients with angiomyolipomata associated with either TSC or sporadic LAM.

10.4.1 Variable

The primary endpoint of this study, angiomyolipoma response rate, is defined as the proportion of patients with an angiomyolipoma response, as defined in Section 7.5.2, using data from the Independent Central Radiological Review of CT/MRIs and the Adverse Events CRF page (to identify angiomyolipoma-related bleeding of grade 2 or worse as defined by NCI CTCAE version 3.0).

10.4.2 Statistical hypothesis, model, and method of analysis

The primary analysis will be a comparison of the angiomyolipoma response rates in the RAD001 and placebo arms using an exact Cochran-Mantel-Haenszel (CMH) test at the one-sided 2.5% level, analyzed in the Full Analysis Set. The test will be stratified by the protocol stratification factor, which has three categories as follows: (1) TSC as underlying disease and patient using enzyme-inducing anti-epileptic drugs (EIAED) at randomization, (2) TSC as underlying disease and patient not using EIAED at randomization, and (3) sporadic LAM as underlying disease.

The statistical hypotheses are

$$H_0: RR_{RAD} \leq RR_{PLB} \quad \text{versus} \quad H_1: RR_{RAD} > RR_{PLB},$$

where RR_{RAD} is the probability of angiomyolipoma response on RAD001 and RR_{PLB} is the probability of angiomyolipoma response on placebo.

Angiomyolipoma response rates will be provided with exact 95% confidence intervals (Clopper and Pearson 1934). The analysis will be performed using data up to the data cut-off date of the trial, which will be 6 months after the last patient is randomized.

10.4.3 Handling of missing values/censoring/discontinuations

Patients with unknown angiomyolipoma response status will be treated as non-responders in the calculation of the angiomyolipoma response rate in the FAS at the end of the trial.

Other missing data will simply be noted as missing on appropriate tables/listings.

10.4.4 Supportive analyses

Potential effect of covariates (prognostic factors) will be investigated using exact logistic regression. The objective of this analysis will be to explore the sensitivity of the statistical significance of treatment effect on angiomyolipoma response rate after adjusting for main prognostic factors. Odds ratios will be used as a measure of association between treatment and response, presented with exact 95% confidence limits. These analyses are considered as supportive.

The first model will include treatment group and the protocol stratification factor as covariates (refer to Section 10.4.2). In a second model, the consistency of the treatment effect will be examined in the presence of possible prognostic factors, as described in Table 10-1, in addition to the stratification factor.

Table 10-1 Definition of potential prognostic factors

Prognostic Factor	Definition	Model covariate definition
Polycystic kidney disease	Presence of polycystic kidney disease at baseline	POLYC (1=present, 0=absent)
Angiomyolipoma volume*	Sum of volumes of target angiomyolipomata at baseline	VOLUME (continuous)

* As determined by Independent Central Radiological Review using the baseline CT/MRI.

The best percentage change from baseline angiomyolipoma volume (defined as the sum of the volumes of the target angiomyolipomata) will be presented graphically by means of a waterfall plot, shown separately for each treatment group.

The primary analysis of angiomyolipoma response rate will be repeated in the Per Protocol Set.

10.5 Secondary objectives

The secondary efficacy objectives were to compare RAD001 against placebo with respect to time to angiomyolipoma progression and skin lesion response rate. In order to be able to make a claim on either of these two endpoints, a multiplicity adjustment will be implemented (see Section 10.5.3).

10.5.1 Time to angiomyolipoma progression

Time to angiomyolipoma progression (TTAP) is defined as the time from the date of randomization to the date of the first documented angiomyolipoma progression, as defined in Section 7.5.2. TTAP will be censored if angiomyolipoma progression is not observed before (i) the cut-off date for the final analysis, or (ii) the date when a non-study systemic anti-angiomyolipoma therapy is started, or (iii) the date of death. The censoring date will be the date of the most recent CT/MRI assessment before the first of any of these three events occurred.

If angiomyolipoma progression is observed after two or more missing or non-evaluable CT/MRI assessments, then the date of angiomyolipoma progression will be censored at the latest occurring CT/MRI. For angiomyolipoma progression observed after a single missing or non-evaluable CT/MRI, the actual date of angiomyolipoma progression will be used.

TTAP will be compared between the RAD001 and placebo arms in the FAS using a one-sided logrank test at the 2.5% level stratified by the protocol stratification factor (TSC and use of EIAED versus TSC and non-use of EIAED versus sporadic LAM).

The TTAP distributions will be presented descriptively in the FAS using Kaplan-Meier curves. Summary statistics from the Kaplan-Meier distributions will be determined, including the median TTAP and the proportions of patients remaining progression-free at 6 and 12 months. These statistics will be given as point estimates with 95% confidence intervals.

The hazard ratio with a 95% confidence interval will be derived from the Cox proportional hazards model, stratified by the protocol stratification factor (TSC and use of EIAED versus TSC and non-use of EIAED versus sporadic LAM).

10.5.2 Skin lesion response rate

Skin lesion response rate is determined only among patients with at least one skin lesion at baseline, and is the proportion of this group of patients with a response (complete clinical response or partial response) on the Physician's Global Assessment of Clinical Condition (PGA), as described in Section 7.5.4.1. Once a patient begins laser treatment or receives local surgery to treat their skin lesions, any data obtained from that point onward will be excluded from the skin lesion response analysis.

Skin lesion response rate will be compared between the RAD001 and placebo arms in patients from the FAS with at least one skin lesion at baseline, using a one-sided exact Cochran-Mantel-Haenszel (CMH) test with a Type I error rate of 2.5%. The test will be stratified by the protocol stratification factor (TSC and use of EIAED versus TSC and non-use of EIAED versus sporadic LAM). The skin response rate will be presented for each treatment arm, along with exact 95% confidence intervals.

10.5.3 Multiplicity adjustment for analysis of main secondary endpoints

Multiplicity will be controlled via a closed testing procedure. Thus, while the hypothesis tests for the two secondary endpoints described in Section 10.5.1 and Section 10.5.2 will both be carried out, the interpretation of the p-values will depend on the hierarchy used in the closed testing strategy. Based on clinical judgment, it was decided to rank the secondary efficacy endpoints in order of importance: time to angiomyolipoma progression, followed by skin lesion response rate. Thus, the closed testing procedure is as follows:

1. Conduct primary analysis to compare RAD001 versus placebo on angiomyolipoma response rate using one-sided exact CMH test. If $p>0.025$ then STOP - otherwise, declare statistically significant benefit of RAD001 on angiomyolipoma response rate, and continue to next step.
2. Compare time to angiomyolipoma progression between RAD001 and placebo using a one-sided, stratified logrank test. If $p>0.025$ then STOP - otherwise, declare statistically significant benefit of RAD001 on time to angiomyolipoma progression, and continue to next step.
3. Compare skin lesion response rate between RAD001 and placebo using one-sided exact CMH test. If $p>0.025$ then STOP - otherwise, declare statistically significant benefit of RAD001 on skin lesion response rate.

For example, the first secondary endpoint, namely time to angiomyolipoma progression, can only be formally declared statistically significant if the p-value is less than or equal to 0.025 and the primary endpoint was statistically significant. Similarly, skin lesion response rate can only be formally tested if both the primary endpoint and the time to angiomyolipoma progression were statistically significant. This approach ensures that the overall Type I error rate of the trial is maintained at 2.5% (one-sided).

10.5.4 Additional secondary efficacy analyses

Duration of angiomyolipoma response

Duration of angiomyolipoma response is defined as the time from the date of the first angiomyolipoma response until the date of the first angiomyolipoma progression, where angiomyolipoma response and angiomyolipoma progression are as defined in Section 7.5.2. Duration of angiomyolipoma response applies only to patients who achieve an angiomyolipoma response. Duration of angiomyolipoma response will be censored if angiomyolipoma progression is not observed before the first to occur out of (i) the cut-off date for the final analysis, or (ii) the date when a non-study systemic anti-angiomyolipoma therapy is started, or (iii) the date of death. The censoring date will be the date of the most recent CT/MRI assessment before the first of any of these three events occurred.

Duration of angiomyolipoma response will be summarized for patients in the RAD001 treatment arm only. A Kaplan-Meier curve will be constructed, and the median response duration will be presented along with 95% confidence intervals. In addition, the Kaplan-Meier estimates with 95% confidence intervals at 3, 6 and 12 months will be summarized.

Time to angiomyolipoma response

Time to angiomyolipoma response is defined as the time from the date of randomization until the date of the first angiomyolipoma response, where angiomyolipoma response is as defined in Section 7.5.2. Time to angiomyolipoma response applies only to patients who achieve an angiomyolipoma response, i.e., patients in the analysis will have known times to response and there will be no censored times. Time to angiomyolipoma response will be summarized only in the RAD001 treatment arm. The median time to response will be presented along with a 95% confidence interval, and the proportions of angiomyolipoma responders who respond by 3 and 6 months will be provided.

Duration of skin lesion response

Duration of skin lesion response is defined as the time from the date of the first skin lesion response until the date of the first skin lesion progression, according to the Physician's Global Assessment (PGA), defined in Section 7.5.4.1. Duration of skin lesion response applies only to patients who achieve a skin lesion response. Duration of response will be censored if skin lesion progression is not observed before (i) the cut-off date for the final analysis, or (ii) the date when a non-study systemic anti-angiomyolipoma therapy is started, or (iii) the date when laser treatment or local surgery to treat skin lesions is started, or (iv) the date of death. The censoring date will be the date of the most recent skin lesion assessment before the first of any of these four events occurred.

Duration of skin lesion response will be summarized for patients in the RAD001 treatment arm only. A Kaplan-Meier curve will be constructed, and the median response duration will be presented along with 95% confidence intervals. In addition, the Kaplan-Meier estimates with 95% confidence intervals at 3, 6 and 12 months will be summarized.

10.5.5 Safety

The assessment of safety will be based mainly on the frequency of adverse events (AEs) and on the number of laboratory values that fall outside of pre-determined ranges. Other safety data (e.g., electrocardiogram, vital signs) will be considered as appropriate. All safety data will be listed.

For all safety analyses, the safety population will be used.

10.5.5.1 Adverse events

All (AEs) recorded during the study will be summarized. The incidence of adverse events will be summarized by body system, severity (based on CTCAE grades), type of adverse event, and relation to the study treatment. Deaths and SAEs will be listed by patient and type of adverse event.

Adverse events will be summarized by presenting the number and percentage of patients having any adverse event in each body system and having each individual adverse event. Any other information collected (e.g., severity or relatedness to study medication) will be listed as appropriate.

In addition, adverse events of related nature may be analyzed by categories regrouping the relevant preferred terms, as appropriate.

10.5.5.2 Laboratory abnormalities

All laboratory values will be converted into SI units and the severity grade calculated using appropriate common terminology criteria for adverse events (CTCAE, version 3.0) unless otherwise indicated.

Renal function will be assessed using the calculated creatinine clearance (CrCl) from the Cockcroft-Gault formula (Cockcroft et al 1976). The proportions of patients in each treatment group with severe renal impairment (defined as calculated CrCl < 30 mL/min) will be compared numerically. In addition, the proportion of patients with NCI CTCAE grade 3/4 serum creatinine will be determined for each treatment group and compared.

A listing of laboratory values will be provided by laboratory parameter and by patient. The frequency of notable lab abnormalities will be displayed by parameter.

Similarly, the frequency of all laboratory abnormalities will be displayed by parameter and worst CTCAE grade experienced.

10.5.5.3 Other safety data

Safety data from other tests (e.g., electrocardiogram or vital signs) will be listed, notable values flagged, and any other information collected will be listed as appropriate. Any

statistical tests performed to explore the data will be used only to highlight any interesting comparisons that may warrant further consideration.

10.5.6 Tolerability

Not applicable for this study.

10.5.7 Resource utilization

Not applicable for this study.

10.5.8 Patient-reported outcomes

Not applicable for this study.

10.5.9 Pharmacokinetics

Pre-dose trough concentrations of RAD001 (C_{min}) and concentration at 2 hours post-dose (± 30 mins, C_{2h}) will be summarized with descriptive statistics and presented graphically over time. Dose proportionality will be explored with a power model using the actual doses to fit log-trough level data. The potential relationships between C_{min} and efficacy/safety endpoints and between C_{2h} and efficacy/safety endpoints will be explored. A logistic regression model will be used for angiomyolipoma response with C_{min} or C_{2h}, with or without log-transformation, as an independent variable and stratified by the protocol stratification factor. The relationship between RAD001 concentration and safety will be explored by modeling events of CTC grade 3 or above with a generalized estimating equation with C_{min} or C_{2h}, with or without log-transformation, as an independent variable and patients as random effects. Appropriate stratification and/or covariates may be applied in the model.

The measured RAD001 PK levels will be used in future analyses, along with RAD001 PK data from other studies (including, but not restricted to: [CRAD001M2301] (SEGA), [CRAD001C2239] (pancreatic neuroendocrine tumors), [CRAD001C2240] (renal cell carcinoma), [CRAD001C2244] (pediatric medulloblastoma), phase 1 studies [CRAD001C2101], [2102], [2104], [2106], [2107], [2108]). These analyses may use non-compartmental and population PK methods with the goal of comparing the results from this protocol with appropriate reference populations (in other tumor types), and thus to characterize PK in the target population with respect to exposure. The population PK analyses across studies will be reported separately, i.e., not as part of the CSR for study M2302.

10.5.10 Biomarkers

The effect of RAD001 on biochemical tumor markers and on angiogenesis markers (e.g., VEGF, basic FGF, PLGF, soluble VEGF receptor1 and soluble VEGF receptor2) will be analyzed using summary statistics for raw data and changes from baseline and also using longitudinal models. Relationships between ligands and corresponding soluble receptors will also be examined both relative to baseline and longitudinally.

Mutational analysis of *TSC1* and *TSC2* genes will be correlated with angiomyolipoma response rate and time to angiomyolipoma progression.

10.5.11 Evaluation after everolimus discontinuation

Patient who discontinue everolimus for reasons other than angiomyolipoma progression, and who subsequently do not receive systemic treatment with an mTOR inhibitor, will be analysed specifically. Data included in this analysis will be data from the follow-up phase (until the end of the extension phase) and/or from the non-interventional follow-up phase.

Reasons for everolimus discontinuation and for non-inclusion in the non-interventional follow-up phase will be presented for this population. A table of demographic characteristics at start of everolimus and duration of everolimus treatment will be produced in order to appreciate the selection biais.

Sum of volumes of target angiomyolipoma lesions (the ones identified before the start of everolimus) will be described at the time of everolimus discontinuation (end of treatment visit or non-interventional follow-up entry visit) and at the time of the one-year follow-up scan (i.e. scan performed one year after everolimus discontinuation), or an earlier scan which was performed following an angiomyolipoma-related bleeding event (≥ grade 2), or prior to any intervention such as the start of systemic mTOR treatment, embolization or nephrectomy.

The effect of stopping everolimus on tumor growth will be described in terms of change and percentage change in the sum of the angiomyolipoma target lesions volume from the start of everolimus and from the time of everolimus discontinuation (scan performed at the end of everolimus treatment). Individual plots of sum of volumes of target angiomyolipoma lesions by time will also be performed to illustrate the tumor evolution after stopping everolimus. Similar analyses will be conducted to describe the changes in kidney volume.

The number of patients with at least one new angiomyolipoma lesion ≥ 1.0 cm in longest diameter and one angiomyolipoma-related bleeding of grade ≥ 2 assessed at the one-year follow-up scan will be tabulated.

If the number of patients included in this analysis is sufficient, the angiomyolipoma progression rate one year after everolimus discontinuation will be provided with exact 95% confidence intervals (Clopper and Pearson 1934). The angiomyolipoma progression will be defined as in Section 7.5.2 of the protocol, i.e. one or more of the following criteria should be observed at the one-year follow-up scan:

- an increase from nadir of 25% or more in angiomyolipoma volume to a value greater than value just prior to everolimus starting date (where angiomyolipoma volume is the sum of the volumes of all target angiomyolipomata identified prior to everolimus start and where nadir is the lowest angiomyolipoma volume achieved by the patient previously in the everolimus period (including the value just prior to everolimus starting date)
- an increase from nadir of 20% or more in the volume of either kidney to a value greater than value just prior to everolimus starting date, where nadir is the lowest kidney volume obtained for the patient, separately for each kidney, previously in the everolimus period (including the value just prior to everolimus starting date)
- the appearance of a new angiomyolipoma ≥ 1.0 cm in longest diameter
- angiomyolipoma-related bleeding grade ≥ 2 as defined by NCI CTCAE, version 3.0.

In addition, if most patients have small AML lesions (less than 1 cm) at the end of everolimus treatment, the sum of the angiomyolipoma target lesions volume identified at the end of

everolimus treatment with a diameter greater than 1 cm will be described at the time of everolimus discontinuation and at the one-year follow-up scan. The change and percentage change in volume from the time of everolimus discontinuation will also be presented.

If the number of patients included in this analysis is sufficient, the angiomyolipoma progression rate 1-year following everolimus discontinuation will be provided with exact 95% confidence intervals (Clopper and Pearson 1934). The angiomyolipoma progression following everolimus discontinuation will be defined as one or more of the following:

- an increase of 25% or more in angiomyolipoma volume from the end of everolimus treatment (where angiomyolipoma volume is the sum of the volumes of target angiomyolipomata identified as greater than 1 cm at the time of everolimus discontinuation)
- an increase of 20% or more in the volume of either kidney from the end of everolimus treatment
- the appearance of a new angiomyolipoma ≥ 1.0 cm in longest diameter
- angiomyolipoma-related bleeding grade ≥ 2 as defined by NCI CTCAE, version 3.0.

10.6 Interim analysis

No interim analysis is planned for this study.

10.7 Sample size calculation

The primary analysis compares angiomyolipoma response rate between the two treatment arms using an exact Cochran-Mantel-Haenszel test (Agresti 2002) in the Full Analysis Set. The randomization is unbalanced, with two patients allocated to RAD001 for every one patient allocated to placebo. In addition, the randomization is stratified by one factor with three categories: (1) TSC as underlying disease and patient using EIAED at randomization, (2) TSC as underlying disease and patient not using EIAED at randomization, and (3) sporadic LAM as underlying disease. It is anticipated that about 75% of patients will have TSC (of the 25 enrolled patients in the recent trial reported by Bissler et al (2008), a total of 19 (76%) had TSC and 6 (24%) had sporadic LAM) and, of the TSC patients, about half are expected to be using EIAED at the time of randomization. That is, the relative prevalence of the categories (1) to (3) of the stratification factor is expected to be 3:3:2. It is planned to use a one-sided test and a 2.5% significance level. The angiomyolipoma response rate in the placebo arm is expected to be close to 0%, since there are no reported cases of spontaneous tumor regression in patients with angiomyolipoma. The angiomyolipoma response rate on RAD001 is anticipated to be at least 20%.

Sample size was determined using simulation (note that software providing sample sizes for exact CMH tests with unbalanced randomization is not readily available). The simulation approach involves randomly generating data according to the study assumptions for a large number of simulated trials, and then analyzing each trial using the exact CMH test. The proportion of times that the test is significant (i.e., has a one-sided p-value ≤ 0.025) gives the study power. Different sample sizes can be assessed, and by trial and error a sample size that guarantees a study power of at least 90% can be chosen. As a starting value, NQuery (V4.0) indicates that for analysis using Fisher's exact test (i.e., a different exact test, and one that

does not take into account the stratification), a total of 99 patients would provide 93% power (2:1 randomization).

The power of the exact CMH test with 99 patients is shown in Table 10-2 below according to the study assumptions, notably a fixed overall angiomyolipoma response rate on RAD001 of 20%, and assuming a 3:3:2 ratio in the number of patients in Stratum 1 (TSC and use of EIAED) versus Stratum 2 (TSC and non-use of EIAED) versus Stratum 3 (sporadic LAM). The row in the table in bold assumes no treatment by stratum interaction; other rows show differing levels of interaction, where at the extreme the angiomyolipoma response rate is assumed to be 0% in one of the strata. As can be seen, the power of the stratified test is highly robust to even the most extreme treatment by strata interaction. The sample size of 99 patients provides at least 93% power in all cases considered.

Table 10-2 Sensitivity of study power to treatment by stratum interaction assuming 3:3:2 ratio of patients across strata 1-3

Ratio of patients across strata 1-3	Angiomyolipoma response rate in RAD001 arm				Power*
	Stratum 1 (TSC with EIAED)	Stratum 2 (TSC without EIAED)	Stratum 3 (sporadic LAM)	Overall	
3:3:2	26.67%	26.67%	0%	20%	93.20%
	20%	33.33%	0%	20%	93.53%
	23.33%	23.33%	10%	20%	93.56%
	16.67%	30%	10%	20%	93.48%
	**20%**	**20%**	**20%**	**20%**	**93.30%**
	10%	30%	20%	20%	93.37%
	13.33%	13.33%	40%	20%	94.09%
	6.67%	20%	40%	20%	93.68%
	0%	0%	80%	20%	98.50%

*Based on 10000 runs, and assumes 66/33 patients on RAD001/Placebo, Placebo response=0%, one-sided exact CMH test at 2.5% level.

The power of the exact CMH test is also highly robust to the assumption of a 3:3:2 ratio in the number of patients across the strata 1-3. For example, consider the case where only 10% of the randomized patients had sporadic LAM: then the relative prevalence of categories 1-3 of the stratification factor would be 4.5:4.5:1. In addition, if only a third of the randomized patients with TSC were taking EIAED at randomization, the relative prevalence of the stratification categories 1-3 would become 3:6:1. Simulations have been run with these (and other) ratios of patients to strata 1-3, and in all cases the study power was maintained at 93% or higher. The power was maintained for varying degrees of treatment by stratum interaction.

Table 10-3 shows the relationship between study power and the size of the treatment effect. The power is rather sensitive to even small changes in the RAD001 response rate; however, with 99 patients the power is at least 86% if the true angiomyolipoma response rate is greater than 18%, and at least 97% if it is greater than 22%. The required increase in sample size to provide sufficient power to detect smaller treatment effects was considered unnecessary (e.g., if the true response rate on RAD001 was 14%, sample size would need to increase by about 35% in order to ensure 90% power).

Table 10-3 Sensitivity of study power to size of treatment effect

Angiomyolipoma response rate		Power* with 99 patients	Number of patients to ensure ≥90% power*
RAD001	Placebo		
14%	0%	58.76%	135
16%	0%	75.29%	117
18%	0%	86.52%	105
20%	0%	93.30%	96
22%	0%	97.02%	87
24%	0%	98.76%	78
26%	0%	99.50%	72

*Based on 10000 runs, and assumes 2:1 ratio for RAD001:Placebo, 3:2:2 ratio for Stratum 1:Stratum 2:Stratum 3, one-sided exact CMH test at 2.5% level.

Taking all these simulation results into account, the total sample size was chosen to be 99 patients, with 66 randomized to RAD001 and 33 randomized to placebo.

11 Administrative procedures

Regulatory and ethical compliance

This clinical study was designed and shall be implemented and reported in accordance with the protocol, the ICH Harmonized Tripartite Guidelines for Good Clinical Practice, with applicable local regulations (including European Directive 2001/20/EC and US Code of Federal Regulations Title 21), and with the ethical principles laid down in the Declaration of Helsinki.

Responsibilities of the investigator and IRB/IEC/REB

The protocol and the proposed informed consent form must be reviewed and approved by a properly constituted Institutional Review Board/Independent Ethics Committee/Research Ethics Board (IRB/IEC/REB) before study start. A signed and dated statement that the protocol and informed consent have been approved by the IRB/IEC/REB must be given to Novartis before study initiation. Prior to study start, the investigator is required to sign a protocol signature page confirming his/her agreement to conduct the study in accordance with these documents and all of the instructions and procedures found in this protocol and to give access to all relevant data and records to Novartis monitors, auditors, Novartis Clinical Quality Assurance representatives, designated agents of Novartis, IRBs/IECs/REBs and regulatory authorities as required.

Informed consent

Eligible patients may only be included in the study after providing written (witnessed, where required by law or regulation), IRB/IEC/REB-approved informed consent, or, if incapable of doing so, after such consent has been provided by a legally acceptable representative of the patient. Informed consent must be obtained before conducting any study-specific procedures (i.e., all of the procedures described in the protocol). In cases where the subject's legally acceptable representative gives consent, the subject (e.g., minors, patients with severe dementia), should be informed about the trial to the extent compatible with the subject's

understanding and if capable, the subject should assent, sign and personally date the written informed consent. The process of obtaining informed consent should be documented in the patient source documents. In emergency situations when prior consent of the subject is not possible and the subject's legally acceptable representative is not available, enrollment of the subject should require measures described in the protocol with documented favorable opinion of the IRB/IEC/REB. The subject or the subject's legally appointed representative should be informed about the trial as soon as possible and consent to continue and other consent as appropriate should be requested.

A proposed informed consent form that complies with the ICH GCP guideline and regulatory requirements and is considered appropriate for this study is provided to each site. Any changes to the proposed consent form suggested by the investigator must be agreed to by Novartis before submission to the IRB/IEC/REB, and a copy of the approved version must be provided to the Novartis monitor after IRB/IEC/REB approval.

Amendments to the protocol

Any change or addition to the protocol can only be made in a written protocol amendment that must be approved by Novartis, Health Authorities where required, and the IRB/IEC/REB. Only amendments that are required for patient safety may be implemented prior to IRB/IEC/REB approval. Notwithstanding the need for approval of formal protocol amendments, the investigator is expected to take any immediate action required for the safety of any patient included in this study, even if this action represents a deviation from the protocol. In such cases, Novartis should be notified of this action and the IRB/IEC/REB at the study site should be informed within 10 working days.

Discontinuation of the study

Novartis reserves the right to discontinue this study under the conditions specified in the clinical trial agreement.

Study drug supply and resupply, storage, and tracking/drug accountability

Study drugs must be received by a designated person at the study site, handled and stored safely and properly, and kept in a secured location to which only the investigator and designated assistants have access. Upon receipt, the RAD001 should be stored according to the instructions specified on the drug labels. Clinical supplies are to be dispensed only in accordance with the protocol.

Medication labels will be in the local language and comply with the legal requirements of each country. They will include storage conditions for the drug and the medication number but no information about the patient.

The investigator must maintain an accurate record of the shipment and dispensing of study drug in a drug accountability ledger. Drug accountability will be noted by the field monitor during site visits and at the completion of the trial. Patients will be asked to return all unused study drug and packaging at the end of the study or at the time of study drug discontinuation.

At the conclusion of the study, and, as appropriate during the course of the study, the investigator will return all used and unused study drug, packaging, drug labels, and a copy of the completed drug accountability ledger to the Novartis monitor or to the Novartis address provided in the investigator folder at each site.

12 Protocol adherence

Investigators ascertain they will apply due diligence to avoid protocol deviations. Under no circumstances should the investigator contact Novartis or its agents, if any, monitoring the trial to request approval of a protocol deviation, as no authorized deviations are permitted. If the investigator feels a protocol deviation would improve the conduct of the study this must be considered a protocol amendment, and unless such an amendment is agreed upon by Novartis and approved by the IRB/IEC/REB it cannot be implemented. All significant protocol deviations will be recorded and reported in the CSR.

13 References (available upon request)

Adler J, Greweldinger J, Litzky G (1984) Macroaneurysm in renal angiomyolipoma: Two cases, with therapeutic embolization in one patient. Urol Radiol; 6: 201-3.

Agresti A (2002) Categorical Data Analysis (Second Edition), Wiley, New York.

Astrinidis A, Henske EP (2005) Tuberous sclerosis complex: linking growth and energy signaling pathways in human disease. Oncogene; 24: 7475-7481.

Astrinidis A, Khare L, Carsillo T, et al (2000) Mutational analysis of the tuberous sclerosis gene *TSC2* in patients with pulmonary lymphangioleiomyo-matosis. J Med Genet. ; 37: 55-7.

Atkins MB, Hildalgo M, Stadler WM, et al (2004) Randomized phase II study of multiple dose levels of CCI-779, a novel mammalian target of rapamycin kinase inhibitor, in patients with advanced refractory renal cell carcinoma. J Clin Oncology; 22(5): 909-918.

Bernstein J, Robbins TO (1991) Renal involvement in tuberous sclerosis. Ann New York Acad Sci; 615: 36-49.

Bissler JJ, Kingswood JC (2004) Renal angiomyolipomata Kidney Int; 66: 924-34.

Bissler JJ, McCormack FX, Young LR, et al (2008) Sirolimus for Angiomyolipoma in Tuberous Sclerosis Complex or Lymphangioleiomyomatosis. New England Journal of Medicine; 358: 140-51.

Bissler JJ, Racadio J, Donnelly LF, et al (2002) Reduction of postembolization syndrome after ablation of renal angiomyolipoma. Am J Kidney Dis; 39: 966-71.

Bittmann I, Rolf B, Amann G, et al (2003) Recurrence of lymphangioleiomyomatosis after single lung transplantation: new insights into pathogenesis. Hum Pathol; 34: 95-8.

Bjornsti MA, Houghton PJ (2004) The TOR pathway. A target for cancer chemotherapy. Nature Reviews Cancer; 4:335-338.

Boulay, Rudloff J, Ye J, et al (2005) Dual inhibition of mTOR and estrogen receptor signaling *in vitro* induces cell death in models of breast cancer. Clin Cancer Res; 11: 5319-28.

Brusasco V, Crapo R, Viegi G (2005) Coming together: the ATS/ERS consensus on clinical pulmonary function testing. Eur Respir J; 26: 1-2.

Capron F, Ameille J, Leclerc P, et al (1983) Pulmonary lymphangioleiomyomatosis and Bourneville's tuberous sclerosis with pulmonary involvement: the same disease? Cancer; 52: 851-5.

Carsillo T, Astrinidis A, Henske EP (2000) Mutations in the tuberous sclerosis complex gene *TSC2* are a cause of sporadic pulmonary lymphangioleiomyomatosis. Proc Natl Acad Sci USA.; 97: 6085-90.

Casper KA, Lane F, Donnelly LF, et al (2002) Tuberous Sclerosis Complex: Renal Imaging Findings. Radiology; 225: 451-456.

Chan JA, Zhang H, Roberts PS (2004) Pathogenesis of tuberous sclerosis subependymal giant cell astrocytomas: biallelic inactivation of *TSC1* or *TSC2* leads to mTOR activation. J Neuropathol Exp Neurol, 63(12): 1236-42.

Chan JK, Tsang WY, Pau MY, et al (1993) Lymphangiomyomatosis and angiomyolipoma: Closely related entities characterized by hamartomatous proliferation of HMB-45-positive smooth muscle. Histopathology; 22: 445-455.

Chesa Ponce N, Artiles Hernandez JL, Ponce Socorro JM, et al (1995) Wunderlich's syndrome as the first manifestation of a renal angiomyolipoma. Arch Esp Urol; 48: 305-308.

Clarke A, Hancock E, Kingswood C, et al (1999) End-stage renal failure in adults with the tuberous sclerosis complex. Nephrol Dial Transplant; 14: 988-91.

Clopper C, Pearson (1934) The use of confidence or fiducial limits illustrated in the case of the binomal. Biometrika 26: 404-413.

Cockcroft DW, Gault MH, et al (1976) Prediction of creatinine clearance from serum creatinine. Nephron; 16: 31-41.

Cohen HT, McGovern FJ, et al (2005) Renal-cell carcinoma. N Engl J Med, 353:2477-90.

Cook JA, Oliver K, Mueller RF, et al (1996) A cross sectional study of renal involvement in tuberous sclerosis. J Med Genet; 33: 480-484.

Cornog JL Jr, Enterline HT (1966) Lymphangiomyoma, a benign lesion of chyliferous lymphatics synonymous with lymphangiopericytoma. Cancer; 19: 1909-30.

Costello LC, Hartman TE, Ryu JH (2000) High frequency of pulmonary lymphangioleiomyomatosis in women with tuberous sclerosis complex. Mayo Clin Proc; 75: 591-4.

Dickinson M, Ruckle H, Beaghler M, et al (1998) Renal angiomyolipoma: Optimal treatment based on size and symptoms. Clin Nephrol; 49: 281-286.

Duvic M, Martin AG, Kim Y, et al (2001) Phase 2 and 3 clinical trial or oral bexarotene (Targretin capsules) for the treatment of refractory or persistent early-stage cutaneous T-cell lymphoma. Arch Dermatol; 137: 581-593.

El-Hashemite, et al (2003) Mutation in TSC2 and activation of mammalian target of rapamycin signaling pathway in renal angiomyolipoma. Lancet; 368:1348-49.

Ewalt DE, Sheffield E, Sparagana SP, et al (1998) Renal lesion growth in children with tuberous sclerosis complex. J Urol; 160: 141-145.

Franz DN, Brody A, Meyer C, et al (2001) Mutational and radiographic analysis of pulmonary disease consistent with lymphangioleiomyomatosis and micronodular pneumocyte hyperplasia in women with tuberous sclerosis. Am J Respir Crit Care Med; 164: 661-8.

Gomez MR, Sampson JR, Whittemore VH (eds) (1999) Tuberous Sclerosis Complex. Oxford: Oxford University Press.

Hancock E, Tomkins S, Sampson J, et al (2002) Lymphangioleiomyomatosis and tuberous sclerosis. Respir Med; 96: 7- 13.

Heald P, Mehlmauer M, Martin AG, et al (2003) Topical bexarotene therapy for patients with refractory or persistent early-stage cutaneous T-cell lymphoma: results of the phase III clinical trial. J Am Acad Dermatol; 49: 801-815.

Henske EP (2003) Metastasis of benign tumor cells in tuberous sclerosis complex. Genes Chromosomes Cancer; 38: 376-381.

Hyman MH; Whittemore VH (2000) National Institutes of Health consensus conference: tuberous sclerosis complex. Arch Neurol; 57: 662-5.

Inoki K, Corradetti MN, Guan K-L (2005) Dysregulation of the TSC-mTOR pathway in human disease. Nature Genetics; 37: 19-24.

Johnson SR (2006) Lymphangioleiomyomatosis. Eur Respir J; 27: 1056-65.

Juvet SC, McCormack FX, Kwiatkowski DJ, et al (2007) Molecular Pathogenesis of Lymphangioleiomyomatosis: Lessons Learned from Orphans. Am J Respir Cell Mol Biol; 36: 398-408.

Karbowniczek M, Astrinidis A, Balsara BR, et al (2003) Recurrent lymphangioleiomyomatosis after transplantation: genetic analyses reveal a metastatic mechanism. Am J Respir Crit Care Med; 167: 976-82.

Krishna G, Moton A, Lei Ma L, et al (2009) Effects of oral posaconazole on the pharmacokinetic properties of oral and intravenous midazolam: A phase I, randomized, open-label, crossover study in healthy volunteers. Clinical Therapeutics; 31: 286-98.

Kwiatkowski DJ, Manning BD (2005) Tuberous Sclerosis: a GAP at the crossroads of multiple signaling pathways. Human Molecular Genetics; 14: R1-R8.

Loomba R, Rowley A, Wesley R, et al (2008) Systematic review: the effect of preventive lamivudine on hepatitis B reactivation during chemotherapy. Ann Intern Med 148(7): 519-28.

Maruyama H, Seyama K, Sobajima J, et al (2001) Multifocal micronodular pneumocyte hyperplasia and lymphangioleiomyomatosis in tuberous sclerosis with a TSC2 gene. Mod Pathol; 14: 609-14.

Mayr NA, Yuh WT, Taoka T, et al (2006) Serial therapy-induced changes in tumor shape in cervical cancer and their impact on assessing tumor volume and treatment response. Am J Roentgenol, 187:65-72.

Miller AB, Hoogstraten B, Staquet M, et al (1981) Reporting results of cancer treatment, Cancer, 47:207-214.

Moss J, Avila NA, Barnes PM, et al (2001) Prevalence and clinical characteristics of lymphangioleiomyomatosis (LAM) in patients with tuberous sclerosis complex. Am J Respir Crit Care Med; 164: 669-71.

Nelson CP, Sanda MG (2002) Contemporary diagnosis and management of renal angiomyolipoma. J Urol; 168: 1315-25.

O’Reilly TM, Wood JM, Littlewood-Evans A, et al (2005) Differential anti-vascular effects of mTOR or VEGFR pathway inhibition: a rational basis for combining RAD001 and PTK787/ZK222584. Proc Am Assoc Cancer Res; 46:715. Abstract 3038.

Oesterling JE, Fishman EK, Goldman SM, et al (1986) The management of renal angiomyolipoma. J Urol; 135: 1121-24.

Partridge SC, Gibbs JE, Lu Y, et al (2005) MRI measurements of breast tumor volume predict response to neoadjuvant chemotherapy and recurrence-free survival. Am J Roentgen; 184: 1774-81.

Pode D, Meretik S, Shapiro A, et al (1985) Diagnosis and management of renal angiomyolipoma. Urol; 25: 461-7.

Rakowski SK, Winterkorn EB, Paul E, et al (2006) Renal manifestations of tuberous sclerosis complex: Incidence, prognosis, and predictive factors. Kidney Int; 70: 1777-82.

Roach ES, Gomez MR, Northrup H (1998) Tuberous sclerosis complex consensus conference: revised clinical diagnostic criteria. J Child Neurol; 13: 624-8.

Sampson JR, Maheshwar MM, Aspinwall R, et al (1997) Renal cystic disease in tuberous sclerosis: role of the polycystic kidney disease 1 gene. Am J Hum Genet; 61: 843- 851.

Sato T, Seyama K, Fujii H, et al (2002) Mutation analysis of the *TSC1* and *TSC2* genes in Japanese patients with pulmonary lymphangioleiomyomatosis. J Hum Genet; 47: 20-28.

Schillinger F, Montagnac R (1996) Chronic renal failure and its treatment in tuberous sclerosis. Nephrol Dial Transplant; 11: 481-5.

Shepherd CW, Gomez MR, Lie JT, et al (1991) Causes of death in patients with tuberous sclerosis. Mayo Clin Proc; 66: 792-6.

Smolarek TA, Wessner LL, McCormack FX, et al (1998) Evidence that Lymphangiomyomatosis is caused by *TSC2* mutations: chromosome 16p13 loss of heterozygosity in angiomyolipomata and lymph nodes from women with lymphangiomyomatosis. Am J Hum Genet; 62: 810-5.

Steiner MS, Goldman SM, Fishman EK, et al (1993) The natural history of renal angiomyolipoma. J Urol; 150: 1782-6.

Strizhefa GD, Carsillo T, Kruger WD, et al (2001) The spectrum of mutations in *TSC1* and *TSC2* in women with tuberous sclerosis and lymphangioleiomyomatosis. Am J Respir Crit Care Med; 163: 253-8.

Tabernero J, Rojo F, Casado E, et al (2005) A Phase 1 study with tumor molecular pharmacodynamic evaluation of dose and schedule of the oral mTOR inhibitor everolimus (RAD001) in patients with advanced solid tumors. [abstract]. J Clin Oncol 2005: 23 16S: 3007.

Therasse P, Arbuck SG, Eisenhauer EA, et al (2000) New guidelines to evaluate the response to treatment in solid tumors. J Natl Cancer Inst; 92: 205-16.

Twombly R (2006) Criticism of Tumor Response Criteria Raises Trial Design Questions, JNCI, 98: 232.

Urban T, Lazor R, Lacronique J, et al (1999) Pulmonary lymphangioleiomyomatosis: a study of 69 patients. Groupe d'Etudes et de Recherche sur les Maladies "Orphelines" Pulmonaires (GERM"O"P). Medicine (Baltimore); 78: 321-37.

Van Baal JG, Smits NJ, Keeman JN, et al (1994) The evolution of renal angiomyolipomata in patients with tuberous sclerosis. J Urol; 152: 35-38.

Webb DW, Kabala J, Osborne JP (1994) A population study of renal disease in patients with tuberous sclerosis. Br J Urol; 74: 151-4.

Yates JRW (2006) Tuberous sclerosis. Europ J Hum Gen; 14: 1065-73.

Young LR, Elwing J, Bissler JJ, et al (2006) Improved airflow and reduction in gas trapping and serum VEGF-D levels in LAM patients treated with sirolimus. American J Resp Critical Med; 173: A241.
